# Phosphoproteomic analysis of dengue virus infected U937 cells and identification of pyruvate kinase M2 as a differentially phosphorylated phosphoprotein

**DOI:** 10.1038/s41598-020-71407-x

**Published:** 2020-09-02

**Authors:** Jeerang Wongtrakul, Thananya Thongtan, Supitcha Pannengpetch, Nitwara Wikan, Doungnapa Kantamala, Benjawan Kumrapich, Warissara Suwan, Duncan R. Smith

**Affiliations:** 1 Research Institute for Health Sciences, Chiang Mai University, 110 Intavaroros Road, Sriphum, Muang District, Chiang Mai, 50200 Thailand; 2 Department of Biochemistry, Faculty of Medicine, Chulalongkorn University, 1873 Rama 4 Road, Pathumwan, 10330 Bangkok Thailand; 3 Center for Research and Innovation, Faculty of Medical Technology, Mahidol University, 999 Phutthamonton 4 Rd, Salaya, 73170 Nakhon Pathom Thailand; 4 Molecular Pathology Laboratory, Institute of Molecular Biosciences, Mahidol University, 25/25 Phuttamonthon 4 Rd, Salaya, 73170 Nakorn Pathom Thailand; 5 Department of Genetics, Faculty of Science, Maejo University, Chiang Mai, 50290 Thailand

**Keywords:** Microbiology, Virology, Dengue virus

## Abstract

Dengue virus (DENV) is an arthropod-borne *Flavivirus* that can cause a range of symptomatic disease in humans. There are four dengue viruses (DENV 1 to 4) and infection with one DENV only provides transient protection against a heterotypic virus. Second infections are often more severe as the disease is potentiated by antibodies from the first infection through a process known as antibody dependent enhancement (ADE) of infection. Phosphorylation is a major post-translational modification that can have marked effects on a number of processes. To date there has been little information on the phosphorylation changes induced by DENV infection. This study aimed to determine global phosphoproteome changes induced by DENV 2 in U937 cells infected under an ADE protocol. A 2-dimensional electrophoretic approach coupled with a phosphoprotein-specific dye and mass spectroscopic analysis identified 15 statistically significant differentially phosphorylated proteins upon DENV 2 infection. One protein identified as significantly differentially phosphorylated, pyruvate kinase M2 (PKM2) was validated. Treatment with a PKM2 inhibitor modestly reduced levels of infection and viral output, but no change was seen in cellular viral protein levels, suggesting that PKM2 acts on exocytic virus release. While the effect of inhibition of PKM2 was relatively modest, the results highlight the need for a greater understanding of the role of phosphoproteins in DENV infection.

## Introduction

Dengue virus (DENV; family *Flaviviridae*, genus *Flavivirus*) is an enveloped, single-stranded positive-sense RNA virus that encodes for 10 proteins (three structural and seven non-structural)^1^. Transmitted to humans primarily by *Aedes* genus mosquitoes infection can result in a range of symptoms from mild to severe. Approximately half the world population is at risk of DENV infection^2^, with three-quarters of these residing in the Asia–Pacific region, with 1.3 billion living in ten DENV endemic countries in Southeast Asia^3^.

There are four serotypes of DENV, DENV 1 to DENV 4. In Thailand, only 5.8% and 4.7% of primary infections were found to be caused by infection with DENV 2 or DENV 4, whereas DENV 1 and DENV 3 caused 27.5% and 29.6% of infections, respectively. Interestingly, secondary infections were found in 91.8% and 90.6% of DENV 2 and DENV 4 infections, and in patients with the clinical presentations of DHF/DSS, DENV 2 and DENV 4 were found in 87.2% and 84.4% of cases, respectively^4^. Similarly, another study reported that almost all of DHF cases caused by DENV 2 and DENV 4 were secondary infections, suggesting that DENV 2 and DENV 4 strains circulating in Thailand need enhancement of infection to cause DHF^5^. An additional study reported that increased DENV disease severity correlated with high viremia titer, secondary DENV infection and DENV 2 virus serotype^6^. In addition, secondary infections caused by DENV 2 was associated with more cases of DHF than were DENV 4 secondary infections^7^.

Preexisting heterologous antibodies have an important role in the development of severe DENV disease. Antibody-dependent enhancement (ADE) of DENV infection has been proposed as the mechanism underlying DHF/DSS^8,9^. DENV cross-reactive antibodies raised following a primary infection combine with a secondary infecting virus to form infectious immune complexes that enter Fc-receptor bearing cells such as monocytes and macrophages as well as immature and mature dendritic cells^8^. ADE of infection is believed to be driven by two main elements. Firstly, there is an increased number of infected cells due to increased antibody-mediated cell binding and entry of both mature and (partially) immature DENV particles which is also known as extrinsic ADE. Another form called intrinsic ADE occurs through increased virus production per infected cell due to suppression of the innate antiviral response^10^. Intrinsic ADE of DENV infection is believed to involve suppression of the toll-like receptor (TLR) and retinoic acid inducible protein I/melanoma differentiation-associated gene5 (RIG-I/MDA5) signaling pathways thereby decreasing the production of type I interferon and interferon-activated antiviral molecules^11^.

While there is an approved commercially available tetravalent vaccine to protect against DENV, its introduction has been controversial due to the occurrence of more severe disease in flavivirus naïve individuals who received vaccination^12^. Currently, there is no specific drug to treat DENV infection. Kinase inhibitors are of particular interest to the development of antiviral agents, since DENV infection can directly activate signal transducer and activator proteins in the MAP kinase pathway e.g. JNK, p38, NTRK1, MAPKAPK5 and c-src/FYN kinases^13–18^. Therefore, kinase inhibitors that affect host cell factors required for virus replication but have no effect on host cells could be an alternative therapy for DENV infection. At present, there are many kinase inhibitors available in the market^19^. JNK and p38 kinase inhibitors were reported to significantly reduced DENV protein synthesis and viral yield^14^. Several DENV-induced pro-inflammatory mediators such as TNF-α, IL-8, and RANTES were also suppressed by a p38 MAPK inhibitor tested in human peripheral blood mononuclear cells (PBMCs), monocytic THP-1 cells, and the granulocyte KU812 cell line. In addition, oral treatment of DENV-infected AG129 mice with SB203580 prevented a rising hematocrit, lymphopenia, inflammation development, intestinal leakage and significantly improved survival^17^. Another kinase inhibitor, SFV785, has selective effects on NTRK1 and MAPKAPK5 kinase activity, and shows anti-viral activity towards hepatitis C, DENV and yellow fever viruses by inhibiting the production of infectious virus particles^13^. Two pharmacological inhibitors of host kinases AZD0530 and dasatinib, have been shown to inhibit the DENV 2 infectious cycle at the step of steady-state RNA replication, and Fyn kinase was identified as the cellular target mediating the effect^16^. Another advantage of some kinase inhibitors is reducing drug resistance caused by the lack of proofreading of RNA virus polymerases, as compound 16i was reported to act as a DENV inhibitor through targeting both the DENV NS5 polymerase and the host kinases c-Src/Fyn^18^. The compound was demonstrated to inhibit DENV replication at low micromolar concentrations with no significant toxicity to the host cell^18^. Additional evidence of a kinase pathway activated by dengue infection is Janus kinase/activator of transcription 3 (JAK/STAT3). JAK2 and JAK3 inhibitors reduced DENV-induced cell migration and production of chemokines such as IL-8 and RANTES^20^.

The majority of previous studies have modeled primary infection, and there is little information of kinases and their cellular targets in secondary DENV infection. Therefore, to explore the differential regulation of kinases in a secondary DENV infection model, phosphoproteomics was employed. Identifying differentially phosphorylated proteins may help in the understanding of host cell factors and cell signaling pathways involved in secondary DENV infection. Therefore, this study focused on a secondary infection model of DENV 2 infection and a phosphoproteome analysis of U937 monocytic cells infected with DENV 2 under conditions of ADE was undertaken using 2-DE gel electrophoresis followed by LC–MS/MS for protein identification. The study identified pyruvate kinase M2 as differentially phosphorylated and the role of this protein in DENV 2 infection was analyzed.

## Results

### ADE infection

ADE infection of U937 cells was optimized using varying concentrations of monoclonal antibody HB-114^21^ and comparing between mock and DENV 2 infected cells. The optimization included a direct infection with no antibody using MOI = 20. After 48 h of infection, it was found that in the absence of antibodies, the percentage of infected cells was 16.340 ± 2.286% (Supplementary Fig. 1), while an antibody dilution of 1:200 resulted in the highest percentage of infection of 69.780 ± 0.710% which was significantly different from the direct infection (*P* < 0.05), confirming the cells were infected under a condition of ADE. A higher concentration of the antibody (1:20 dilution), resulted in a reduced level of infection of 30.173 ± 0.418%, which was still significantly higher than direct infection *P* < 0.05. Consistent with our previous study, no neutralization was observed^22^. Therefore, an antibody dilution of 1:200 was selected for further large scale preparation of DENV 2 infected U937 cells.

### Large scale preparation of infected U937 cells

U937 cells were propagated in T-175 cm^2^ tissue culture flask for 3 days after which cells were collected and the cell density was adjusted to 1.82 × 10^6^ cells/ml. For the preparation of the antibody-virus complex, DENV 2 strain 16681 (multiplicity of infection (MOI) of 20) was mixed with monoclonal antibody HB-114^21^ at a final dilution of 1:200 and incubated for 1 h at 4 °C. Then the complex mixture was added to 3 × 10^7^ U937 cells which were incubated at 37 °C, 5% CO_2_ for 2 h. Finally, culture media was added to the cells to give a final cell density of 3 × 10^5^ cells/ml. Fresh culture media was added every 24 h, and the culture was incubated for 2 days. For mock infected cells, the culture media was mixed with only antibody HB-114 and the same procedure as the protocol of virus infection was followed. The morphology of the cells was monitored daily by observation under an Olympus light microscope, and morphology of mock and DENV 2 infected cells were similar, although some rounding was observed in DENV 2 infected cells on day 2 post-infection (p.i.) (Supplementary Fig. 2). On day 2 p.i. the level of infection was determined by flow cytometry. Results (Supplementary Fig. 3A–C) showed that 77.731 ± 1.346% of cells in the infection arm were infected, while the mock infections showed only background signal (0.454 ± 0.027%). The percentage cell survival was determined by a trypan blue dye exclusion assay, and showed 96.000 ± 0.408% and 92.500 ± 1.443% cell survival in mock and infected cells respectively (Supplementary Fig. 3C). All experiments were undertaken as three independent experiments, with duplicate assay of each point.

### Phosphoprotein enrichment and 2-D gel electrophoresis

**Figure 1:**
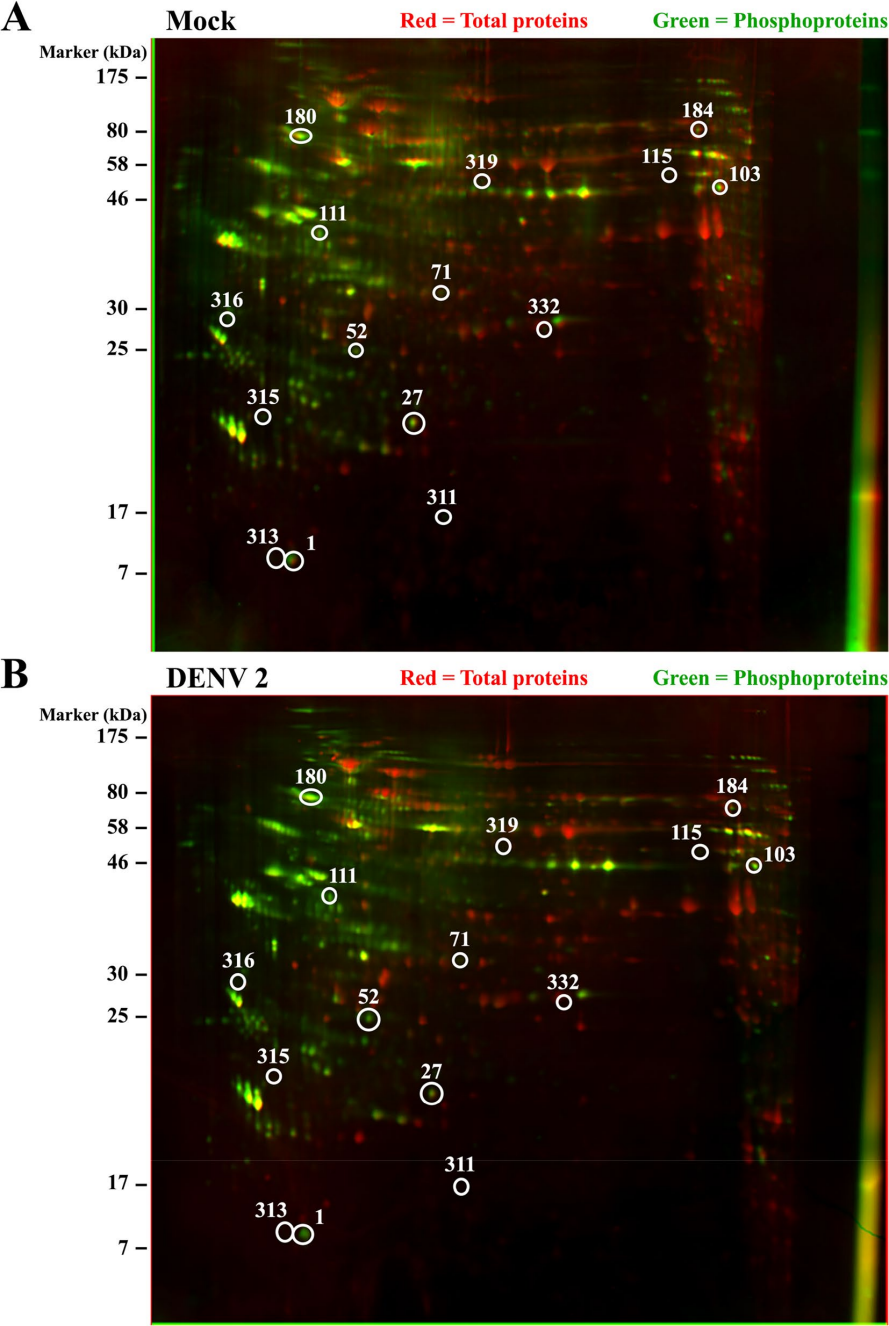
2-DE gels of purified phosphoproteins from U937 Mock and DENV 2 infected cells. Dual view of gels stained with ProQ Diamond (Green) and subsequently with SYPRO Ruby (red) to detect specifically phosphoproteins and total proteins, respectively. Mass spectrometry identified phosphoproteins are labelled by numbers corresponding to their identification demonstrated in Table 1 (**A**) Dual view of ProQ Diamond and SYPRO Ruby staining of Mock protein extracts after phosphoprotein enrichment. (**B**) Dual view of ProQ Diamond and SYPRO Ruby staining of DENV 2 protein extracts after phosphoprotein enrichment.

The total protein obtained from the sonication of approximately 7.5–8.0 × 10^7^ mock or DENV 2 infected cells ranged from 3,700–5,000 μg per sample. For phosphoprotein enrichment, approximately 3,700–4,000 μg protein was loaded onto a pre-equilibrated phosphoprotein affinity column for 30 min at 4 °C. Columns were washed three times to remove non-specific binding proteins, and phosphoproteins were eluted in 5 fractions. Aliquots of each purified fraction were examined by SDS-PAGE (Supplementary Fig. 4). The five elution fractions from each sample were pooled and concentrated using a 10 kDa cut-off concentrator. The yield of phosphoproteins ranged from 9–15 percent of the total amount of protein loaded onto the column, and the concentrated enriched phosphoproteins were also analyzed by SDS-PAGE (Supplementary Fig. 4). All purifications were performed independently in triplicate. Samples were then separated by 2D PAGE using 300 µg enriched phosphoproteins, and gels were stained with ProQ Diamond to detect phosphoproteins and subsequently with SYPRO Ruby to detect total proteins. Representative dual view 2-D gels are shown in Fig. 1, and individual replicate gels are provided in Supplemental materials.

The majority of phosphoproteins from both mock infected and DENV 2 infected cells were focused between pI 4–7 with the protein molecular weight ranging from 17–175 kDa, while the Sypro Ruby stained proteins focused evenly between pI 3–10 (Fig. 1), suggesting that U937 possesses more acidic phosphoproteins than basic phosphoproteins. In the mock and DENV 2 samples there was a mean of 349 and 362 phosphoprotein spots and a mean of 817 and 787 total protein spots respectively, based on the triplicated gels. Analysis of the ProQ Diamond stained gels revealed fifteen phosphoprotein spots that were differentially phosphorylated. Seven phosphoprotein spots showed increased phosphorylation, while eight phosphoprotein spots showed reduced phosphorylation. The mean percent gel volumes of the 15 differentially phosphorylated proteins are shown in Supplementary Fig. 5. In addition, the ratio between total protein and the phosphorylation signal was determined. With this analysis it was found that 14 of the phosphoprotein spots retained statistical significance (Supplementary Fig. 6). The ratio of spot 319 was the only one that showed no significant difference, possibly due to some variation in the mock spot intensity. However, there was no phosphoprotein signal for this spot in the infected samples, suggesting significant downregulation of phosphorylation as seen in the first analysis. Total protein analysis from the SYPRO Ruby stained gels identified 11 total protein spots that were differentially expressed, with seven proteins being up-regulated, two proteins being down-regulated and two spots were found only in DENV 2 infected samples (data not shown). None of the 15 phosphoprotein spots were located at the same position as the 11 differentially expressed total protein spots indicating that the intensity differences noted in the phosphoproteins were due to differential phosphorylation alone, and not due to alterations in expression levels.

### Identification of differentially expressed phosphoproteins

All 15 differentially phosphorylated phosphoprotein spots were identified by LC/MS–MS. Table 1 summarizes the phosphoproteins identified with their accession numbers, molecular weight, pI, MOWSE score, sequence coverage and their biological process. The comparison between DENV 2 infection and mock infection showed that 7 proteins, namely albumin, endoplasmic reticulum resident protein 29, mitochondrial import receptor subunit TOM34, elongation factor 1-delta, glyceraldehyde-3-phosphate dehydrogenase, protein disulfide isomerase A1 and pyruvate kinase M2 showed increased phosphorylation (more than 1.5-fold compared to the mock), while immunoglobulin light chain variable region, 14-3-3 protein gamma, cytochrome c oxidase subunit 5A, UTP-glucose-1-phosphate uridylyltransferase, profilin-2, nascent polypeptide associated complex subunit alpha, nucleophosmin and methylthioadenosine phosphorylase showed significantly reduced levels of phosphorylation in DENV 2 infected samples.

**Table 1:** Phosphoproteins of U937 during ADE infection identified by LC–MS/MS after Pro-Q Diamond staining of 2DE separated enrichment fractions.

Spot no	Accession no	Protein name (phosphorylation*)	MW (kDa)	Calculated pI value	Protein score^a^	Sequence coverage (%)	Biological process^b^
1	gi662564295	Immunoglobulin light chain variable region (down)	11.50	5.09	19	17	Unknown
27	P61981	14–3-3 protein gamma (down)	28.45	4.8	59	13	Regulation of signal transduction
52	gi6650826	Albumin (up)	30.08	6.97	58	5	Transport protein involved in cellular protein metabolic process and cellular response to starvation
71	P30040	Endoplasmic reticulum resident protein 29 (up)	29.03	6.77	49	3	Intracellular protein transport
103	Q15785	Mitochondrial import receptor subunit TOM34 (up)	34.93	9.12	173	5	Protein targeting to mitochondrion
111	P29692	Elongation factor 1-delta (up)	31.21	4.9	61	6	mRNA transcription, regulation of cell death
115	P04406	Glyceraldehyde-3-phosphate dehydrogenase (up)	36.20	8.57	39	17	Canonical glycolysis, glycolytic process and gluconeogenesis
180	P07237	Protein disulfide-isomerase A1 (up)	57.48	4.76	138	12	Cell redox homeostasis, cellular protein metabolic process
184	P14618	Pyruvate kinase M2 (up)	58.47	7.96	196	30	ATP biosynthetic process and glycolytic process
311	P35080	Profilin-2 (down)	15.37	6.55	48	10	Actin cytoskeleton organization
313	P20674	Cytochrome c oxidase subunit 5A (down)	16.92	6.30	45	6	Mitochondrial electron transport
315	Q13765	Nascent polypeptide-associated complex subunit alpha (down)	23.37	4.52	46	3	Protein transport, translation
316	P06748	Nucleophosmin (down)	32.72	4.64	48	5	CENP-A containing nucleosome assembly and negative regulation of cell population proliferation
319	Q16851	UTP-glucose-1-phosphate uridylyltransferase (down)	57.07	8.16	45	5	Glucose 1-phosphate metabolic process, glycogen biosynthetic process
332	Q13126	Methylthioadenosine phosphorylase (down)	31.74	6.75	65	8	L-methionine salvage from methylthioadenosine and methylation

*Up (phosphorylation increased in DENV infection), down (phosphorylation decreased in DENV infection).

^a^Protein score is an ion score obtained from the analysis with Mascot software. Ions score is − 10 * Log(*P*), where *P* is the probability that the observed match is a random event. Protein score indicates the confidence of the protein identification; score value greater than 30 was considered significant (*P* < .05).

^b^Biological process information obtained from UniProt databases (https://www.uniprot.org).

### Analysis of differentially expressed phosphoproteins and validation

**Figure 2:**
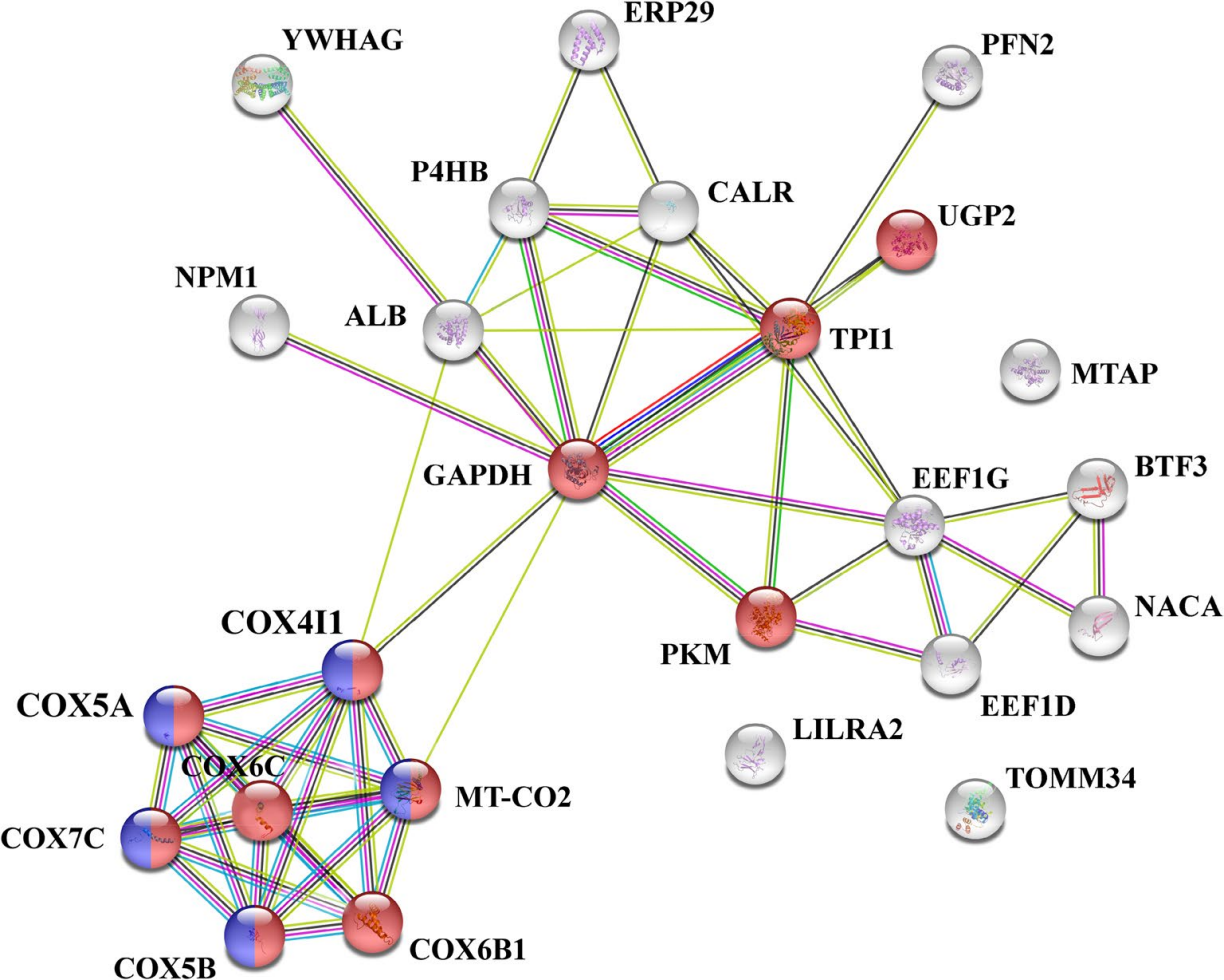
STRING analysis of 15 phosphoproteins altered in response to antibody dependent enhancement of DENV 2 infection. A total of 15 phosphoproteins identified as differentially expressed in response to antibody dependent enhancement of DENV 2 infection were submitted to the STRING database for analysis. Proteins identified as part of the generation of precursor metabolites and energy are shown in red, while proteins identified as part of mitochondria electron transport, cytochrome c to oxygen are shown in blue. Proteins involved in both processes are shown as dual colored.

To determine the association between the differentially phosphorylated phosphoproteins, the 15 identified phosphoproteins from Table 1 were subjected to a pathway analysis using the STRING (Search Tool for the Retrieval of Interacting Genes/Proteins) database (Fig. 2 and Supplementary Table 1). Sixty-two biological process pathways were identified, including generation of precursor metabolites and energy (11 proteins; false discovery rate 8.45e^−10^), mitochondrial electron transport, cytochrome c to oxygen (5 proteins; false discovery rate 1.90e^−08^) and ATP metabolic process (8 proteins; false discovery rate 2.48e^−08^). The overall protein–protein interaction (PPI) enrichment p-value was 1.48e^−06^. In summary STRING identified phosphoproteins involved in glycolysis including mitochondrial processes.

**Figure 3:**
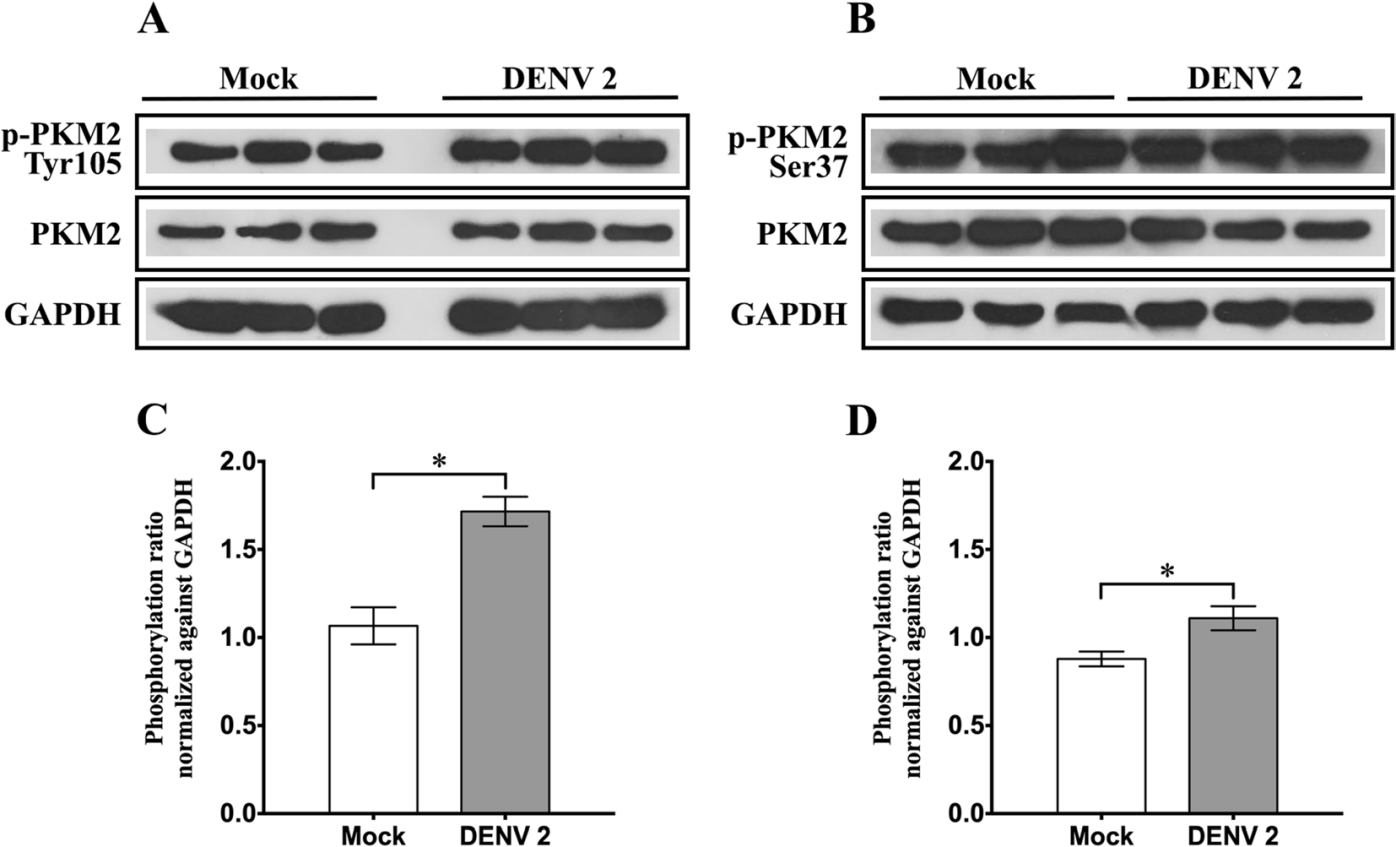
Western blot analysis of phosphorylated pyruvate kinase M2 at amino acid tyrosine 105 (Tyr105) and serine 37 (Ser37). U937 mock infected and DENV 2 infected cells were collected at 48 h. The cell lysates were prepared which were subjected to SDS-PAGE and western blot analysis to detect the expression of (**A**) p-PKM2 Tyr105 and (**B**) p-PKM2 Ser37 as well as (**A**,**B**) total PKM2 and glyceraldehyde-3-phosphate dehydrogenase (GAPDH). The experiment was performed independently in triplicate. (**C**,**D**) Protein band intensities were quantitated using the ImageJ image analysis program and analyzed by GraphPad Prism 5 program and the expression of all proteins were normalized to GAPDH. Error bars show mean +/- SEM of three experiments performed in triplicate. Data were analyzed by Unpaired t-test; *P< 0.05.

To validate the phosphoproteomic analysis, PKM2 was selected for validation. U937 cells were mock infected or DENV 2 infected, and the levels of phosphoryation of PKM2 at Tyr 105 and Ser 37 were determined, together with the expression levels of PKM2 and GAPDH. Results (Fig. 3) showed an increase in PKM2 phosphorylation at both amino acids in DENV 2 infected samples as compared to mock infected samples, consistent with the original phosphoproteomic analysis. Similarly, consistent with the phosphoproteomic analysis, no differences were seen in expression levels of PKM2 when comparing between mock and DENV 2 infected samples.

### PKM2 inhibitor and PKM2 activator cytotoxicity test

To determine the role of PKM2 phosphorylation in DENV 2 infection, we evaluated the effects of a PKM2 inhibitor and activator. First the cytotoxic effects of the PKM2 inhibitor and PKM2 activator on U937 cells were determined using the MTS assay at 24 and 48 h post-treatment. For the PKM2 inhibitor, the concentration range evaluated was from 1–1,000 µM. The calculated IC_50_ value of PKM2 inhibitor was 230.467 ± 13.639 µM and 226.967 ± 20.200 µM at 24 and 48 h post-treatment, respectively (Supplementary Fig. 7). Analysis of cell survival revealed a significant reduction in cell viability following treatment at 200 µM (81.451 ± 5.535%), 400 µM (22.239 ± 4.327%), 600 µM (8.786 ± 2.532%), 800 µM (18.163 ± 4.014%) and 1,000 µM (12.520 ± 6.464%) at 24 h post-treatment. Similarly, reduced viability was observed at 48 h post-treatment at the same five concentrations (200 µM (82.720 ± 4.492%), 400 µM (15.184 ± 1.948%), 600 µM (15.360 ± 4.427%), 800 µM (21.738 ± 3.396%) and 1,000 µM (9.857 ± 3.281%)). For the PKM2 activator, the concentration range evaluated varied between 1 and 200 µM (Supplementary Fig. 8). The calculated IC_50_ values at 24 and 48 h post-treatment were 82.75 ± 11.060 µM and 80.09 ± 4.407 µM, respectively. At 24 h, the PKM2 activator treatment showed significantly decreased cell viability at 40 µM (73.157 ± 3.899%), 60 µM (55.939 ± 3.296%), 80 µM (43.114 ± 2.172%), 100 µM (29.130 ± 2.543%) and 200 µM (6.108 ± 1.704%). Similarly, there were significant reductions in viability at 48 h post-treatment at the same concentrations (40 µM (81.868 ± 6.480%), 60 µM (79.961 ± 6.025%), 80 µM (53.493 ± 8.540%), 100 µM (41.648 ± 6.220%) and 200 µM (4.070 ± 2.237%)). Therefore, concentrations of 100 µM (PKM2 inhibitor) and 20 µM (PKM2 activator) were chosen for further studies to determine the role of PKM2 phosphorylation in DENV 2 infection.

### Virucidal potential of PKM2 inhibitor and PKM2 activator against DENV 2

To further investigate the mechanism of action of PKM2 inhibitor/PKM2 activator, the possibility that both compounds were capable of directly inactivating infectious DENV particles was explored. DENV 2 was incubated with PKM2 inhibitor at 1, 50 and 100 µM and with PKM2 activator at 1, 10 and 20 µM at 37 °C for 1 h. After the incubation, the samples were titrated on LLC-MK2 cells. Results (Supplementary Fig. 9) showed that neither compound had a direct virucidal activity.

### Effect of PKM2 kinase inhibitor/activator on DENV 2 infection and virion production

**Figure 4:**
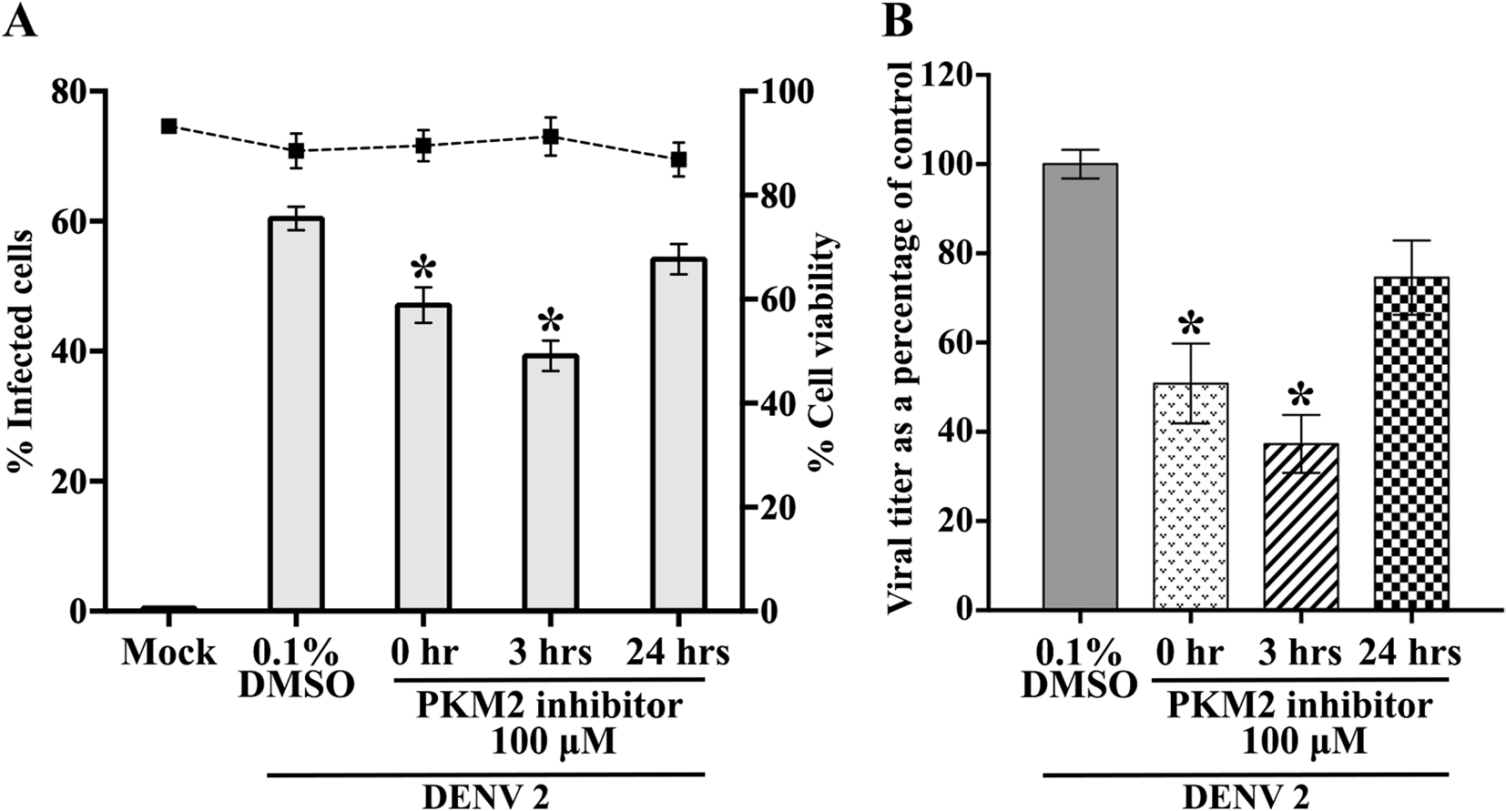
Effect of post-addition of pyruvate kinase M2 inhibitor on DENV 2 infection. U937 cells were incubated with 100 μM PKM2 inhibitor or with vehicle only or not treated at 0, 3 and 24 h after mock infection or infection with DENV 2. (**A**) Cells were collected to determine the cell viability by trypan blue staining and infection level by flow cytometry or (**B**) Supernatants were collected to determine virus titer by standard plaque assay. Experiments were undertaken independently in triplicate with duplicate plaque assay. Bars show mean ± SEM (*P value < 0.05).

A time-of addition analysis was performed using the PKM2 inhibitor or the PKM2 activator to post-treat DENV 2 infected cells at 0-, 3- and 24 h p.i., in parallel with mock and vehicle treated cells. Cells were analysed by flow cytometry to determine the percentage of infection, and additionally cell viability was determined. Results (Fig. 4A) showed that cells treated with PKM2 inhibitor at 0 and 3 h p.i., showed a significant reduction in levels of infection, with a maximal effect of a reduction of 33% being seen with treatment at 3 h p.i. Markedly, no effect was observed upon cell viability (Fig. 4A). Analysis of the supernatants of DENV 2 infected cells by plaque assay showed that the PKM2 inhibitor significantly decreased DENV 2 production by 0.331–0.442 log_10_ at 0 and 3 h (Fig. 4B). In contrast, the same experiment using the PKM2 activator showed no effect on the level of DENV 2 infection or on DENV 2 production (Supplementary Fig. 10).

### Effect of PKM2 kinase inhibitor/activator on DENV 2 protein expression

**Figure 5:**
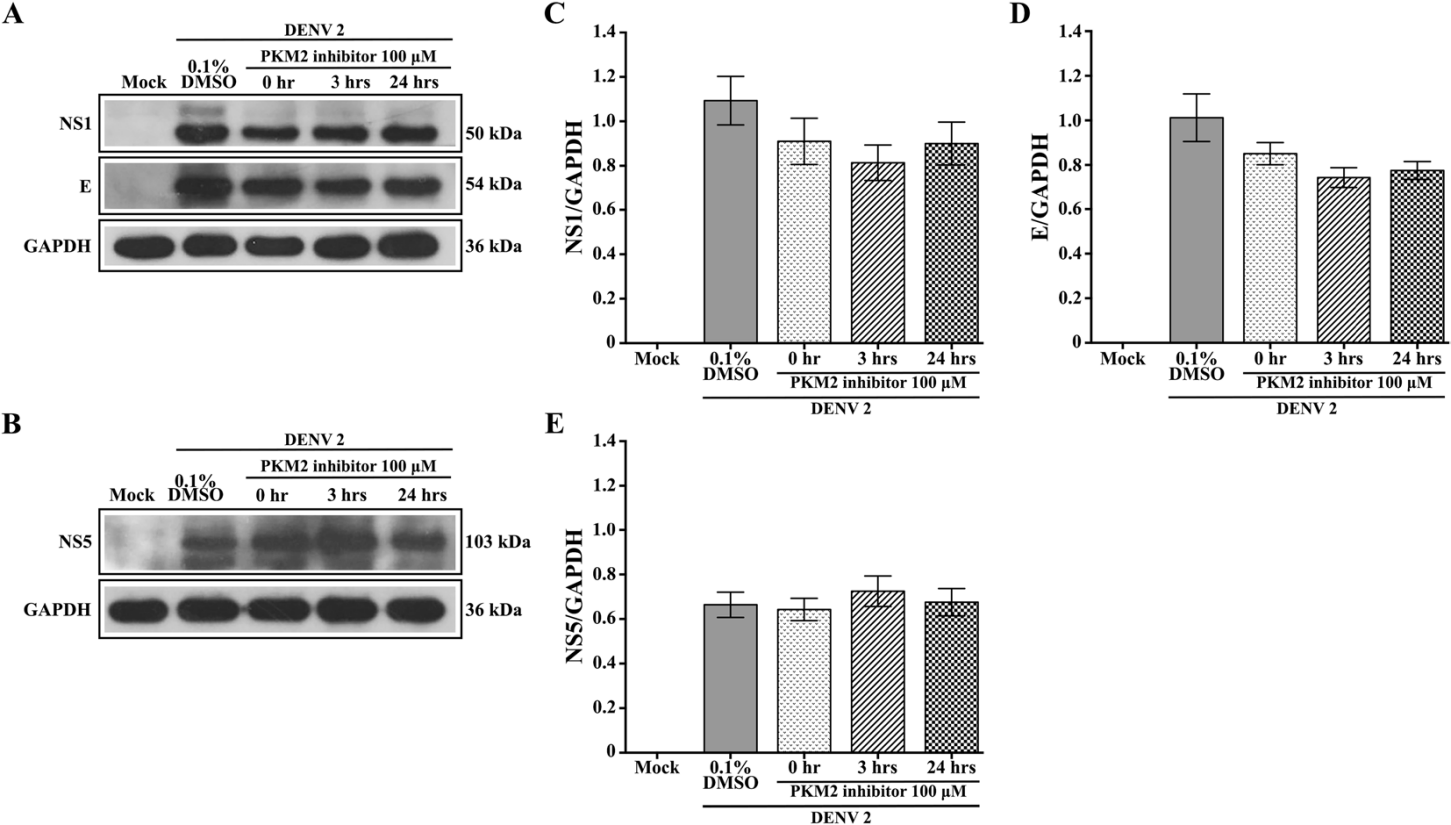
Expression of DV proteins upon treatment with PKM2 inhibitor. U937 cells were infected or mock infected with DENV 2 followed by incubation with 100 μM PKM2 inhibitor or with DMSO vehicle for 0, 3 and 24 h under standard conditions. Cellular lysates were prepared, subjected to western blot analysis and analyzed for the presence of DENV 2 (**A**) NS1 and E and (**B**) NS5 proteins. Protein band intensities from A and B were quantitated for (**C**) NS1, (**D**) E and (**E**) NS5 using ImageJ image analysis software and the expression of all proteins was normalized to GAPDH. Bars represent mean ± SEM of three independent experiments performed in duplicate. Data were analyzed by One-way ANOVA with Bonferroni’s multiple comparison test. No significant difference (*P* > 0.05) in expression profile was observed for NS1, E and NS5 among different time points.

**Figure 6:**
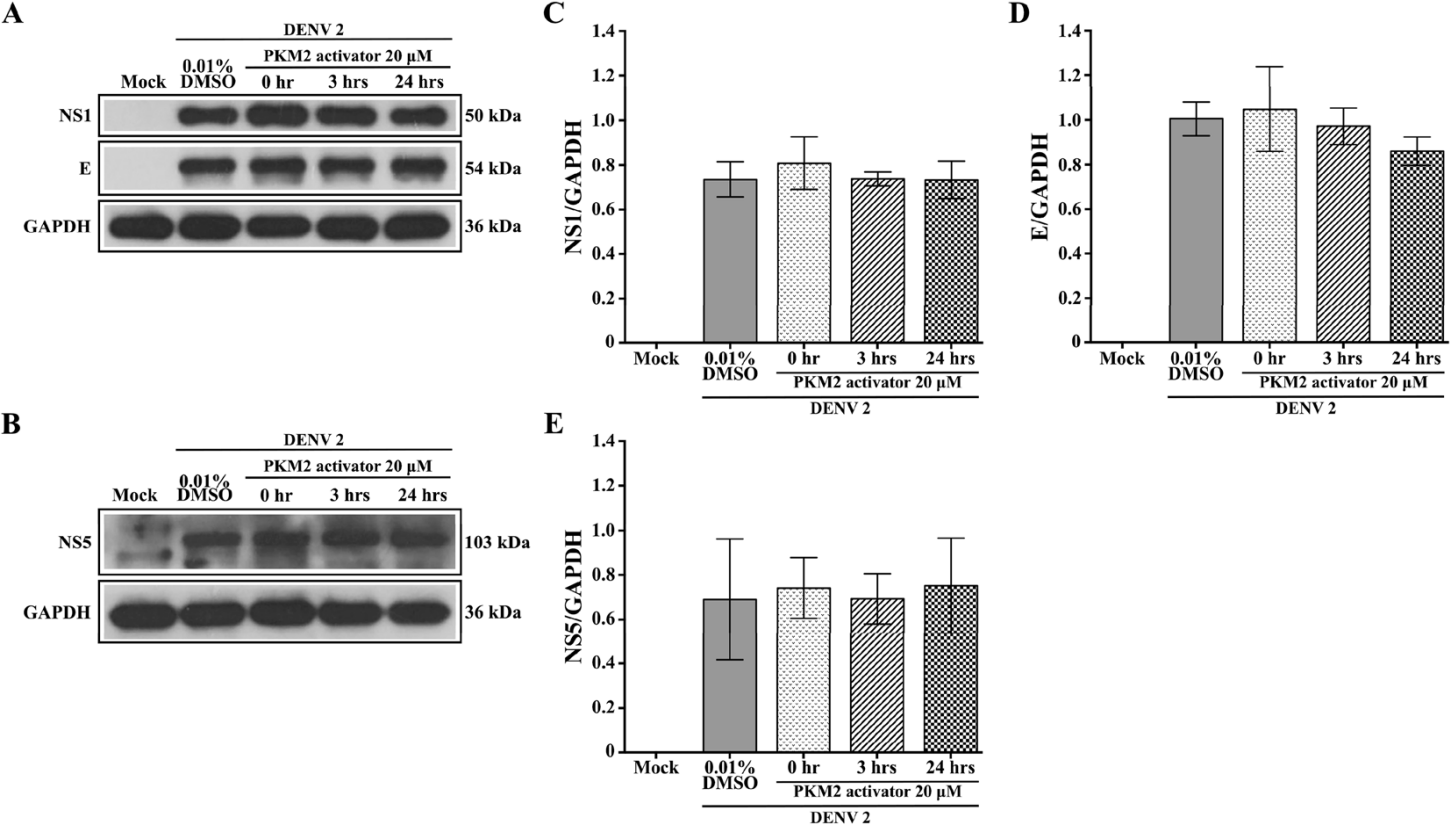
Expression of DV proteins upon treatment with PKM2 activator U937 cells were infected or mock infected with DENV 2 followed by incubation with 20 μM PKM2 activator or with DMSO vehicle for 0, 3 and 24 h under standard conditions. Cellular lysates were prepared, subjected to western blot analysis and analyzed for the presence of DENV 2 (**A**) NS1 and E and (**B**) NS5 proteins. Protein band intensities from A and B were quantitated for (**C**) NS1, (**D**) E and (**E**) NS5 using ImageJ image analysis software and the expression all proteins was normalized to GAPDH. Error bars represent mean +/- SEM of at least three independent experiments performed in duplicate. Data were analyzed by One-way ANOVA with Bonferroni’s multiple comparison test. No significant difference (*P* > 0.05) in expression profile was observed in NS1, E and NS5 among different time points.

To determine the effect of modulation of PKM2 on DENV 2 protein expression, cells were again infected under an ADE-infection protocol and were then treated with the PKM2 inhibitor or PKM2 activator at 0, 3 and 24 h p.i. At 48 h p.i., proteins were extracted and expression of the structural E protein and the non-structural NS1 and NS5 proteins were determined by western blot analysis. The results (Figs. 5 and 6) showed no change in the level of expression of any of the proteins investigated.

## Discussion

DENV infections are a significant public health problem in many parts of the world^23^, and evidence has suggested that severe manifestations are associated with second infections, particularly where the second infection is with DENV 2 or 4^4–7^. There have been several proteomic analysis of DENV infection in cell culture systems^24–32^, and at least one combined proteome and phosphoproteome analysis^33^, but all of these studies have been undertaken modeling primary infections. In this study, U937 cells were infected under conditions of ADE with DENV 2 to model secondary infection, and based on a phosphoproteome analysis 15 phosphoproteins were identified as significantly differentially phosphorylated. Seven proteins showed increased phosphorylation, two showed reduced phosphorylation and six proteins showed no evidence of phosphorylation in DENV 2 infected cells (highly down-regulated phosphorylation).

Four of the 15 identified phosphoproteins (COX5A, UGP2, GAPDH and PKM2) are involved in the generation of precursor metabolites and energy processes, suggesting a significant role for these processes in DENV infection. Two of these proteins (COX5A and UGP2) showed a loss of phosphorylation as compared to mock infected cells, while two (GAPDH and PKM2) showed increased phosphorylation in DENV 2 infected cells as compared to mock infected cells. In terms of protein selection for further verification and evaluation, PKM2 was selected as this had the highest protein score, suggesting that this protein was identified with high confidence and, in addition, commercially available antibodies to both PKM2 and phospho-PKM2 were readily available.

Two previous studies have implicated PKM2 as having a role in DENV infection. Firstly Pando-Robles and colleagues^29^ identified PKM2 as being down-regulated in DENV infected hepatocyte Huh-7 cells, while it was subsequently identified as up-regulated in U937 cells infected with DENV 2 through a direct infection protocol^34^. In this study, the increased phosphorylation of PKM2 detected was not associated with a spot that showed differential expression levels. Thus, it is possible that expression of PKM2 is modulated in DENV infection in a cell type specific manner. Pyruvate kinase (PK) is a rate-limiting glycolytic enzyme that catalyze the transphosphorylation between phosphoenolpyruvate (PEP) and adenosine diphosphate, which produces pyruvate and ATP and plays a role in regulating cell metabolism^35^. There are four mammalian pyruvate kinase isoforms: liver-type PK (PKL); red blood cell PK (PKR); and PK muscle isozyme M1 and M2 (PKM1 and PKM2, respectively). Most adult tissues express PKM2, and expression of the other three isoforms is tissue-specific and regulated by various promoters and alternative splicing^36^. PKM2 has been reported to be modified by phosphorylation at many positions e.g. tyrosine, serine and threonine in response to various stimuli thereby modulating its structure and function properties^37^, and both Tyr105 and Ser37 were phosphorylated at significantly higher levels in DENV 2 infected cells than in mock infected cells. Phosphorylation of PKM2 at Y105 results in inhibition of its catalytic activity, and diversion of glycolytic flux into biosynthetic metabolism promoting the Warburg effect, a mechanism commonly found in tumor cells^38^. In DENV infection, it has been shown that glycolysis is induced and is necessary for efficient DENV replication^39^ and thus it is likely that the effects of PKM2 inhibition seen here are unrelated to the glycolytic functions of PKM2.

Inhibition of PKM2 resulted in a small but significant decrease in DENV infection levels and virus output. The inhibitor used in this study functions through the inhibition of the fructose-1,6-bisphosphate- (FBP-) dependent activation of pyruvate kinase PKM2 which gives rise to an inactive tetramer and inhibits pyruvate kinase activity. This results in decreased aerobic glycolysis and PKM2 phosphorylation^40,41^. As noted, while it is possible that the inhibition of PKM2 results in the decrease of DENV infection and virus production, the fact that cellular protein levels were apparently unaffected would argue against this as a mechanism of action. While the primary function of PKM2 is involved in regulation of glycolysis in the cytosol, it is known that PKM2 can also be found in the mitochondria and nucleus. In the mitochondria it is believed that PKM2 acts to limit ROS-induced apoptosis in cancer cells^42^. DENV infection has been shown to promote increased ROS levels^43,44^, and cellular ROS levels have been shown to control antiviral processes and cell death in DENV infected cells^45^. In the nucleus PKM2 can act as a transcription factor, inducing glycolysis gene expression through c-MYC^46^ as well as acting as a co-activator of the STAT5A transcription factor^47^. However while West Nile Virus and Zika virus have been shown to block STAT5 phosphorylation, DENV and yellow fever virus were shown not to block this phosphorylation^48^, and so it is unlikely that PKM2 is exerting its effect through this pathway.

PKM2 has also been shown to play a role in exosome release through phosphorylation of synaptosomal-associated protein 23 (SNAP-23)^49^. SNAP-23 is known to control the dock and release of secretory granules and exosomes. The release of DENV from host cells remains comparatively under-investigated. Studies using electron microscopy have suggested that virions are released by exocytosis^50,51^ and a more recent study using correlative scanning-transmission electron microscopy suggested that chimeric flavivirus virus particles were released as individual particles in small exocytosis vesicles^52^. The studies using electron microscopy are supported by a study that identified exocyst complex component 7 (EXOC7 or EX070), a part of the exocyst complex that regulates vesicular trafficking and the late stages of exocytosis, as necessary for virus egress^53^. Thus it is possible that inhibition of PKM2 reduces virus egress, resulting in a reduction in the number of infected cells and reduced titer. Given that this does not affect virus translation, this would be consistent with the results of the western blotting in which no reduction of viral protein expression was observed. Although the number of infected cells is reduced as egress is diminished, the level of protein per infected cell would be higher, resulting in no net overall change.

Overall, this project has identified a number of phosphoproteins that are differentially phosphorylated in response to DENV 2 infection. These proteins are involved in a number of processes including generation of precursor metabolites and energy. One protein, PKM2 was validated and inhibition of phosphorylation was shown to affect level of infection and virus titer. It is possible that this effect results from modulation of virus egress. While the effect of inhibition was relatively modest, the results highlight the need for a greater understanding of the role of phosphoproteins in DENV infection, and that studies on the exocytic release of DENV are particularly required.

## Materials and methods

### Cells and viruses

U937 (ATCC CRL-1593), C6/36 (ATCC CRL-1660) and LLC-MK2 (ATCC CCL-7) cells were cultured and DENV 2 (strain16681) propagated and virus titer determined exactly as previously described^22^.

### Standard plaque assay

For quantification of virus titer standard plaque assay was undertaken in, LLC-MK2 cells exactly as previously described^22^.

### ADE infection in U937 cells

To obtain the appropriate antibody dilution, monoclonal antibody HB114^21^ was tenfold serially diluted from 10^–1^–10^–6^ with RPMI 1640 in triplicate. A control tube without the presence of the antibody was also set up. DENV 2 virus was prepared at an MOI of 20 in RPMI and placed on ice. To form virus-antibody complexes, each antibody dilution was incubated with DENV 2 in 0.5 ml tubes at 4 °C for 1 h by gently inverting the tubes every 20 min. The final dilution of antibodies ranged from 1/20 to 1/2,000,000. U937 cells in RPMI medium containing no FBS were seeded as 5 × 10^5^ cells/well in 6-well plates. ADE infection was induced by adding the DENV 2-immune complex to U937 cells and incubating for 2 h at 37 °C with 5% CO_2_ with rocking of the plates every 30 min. After 2 h incubation, complete RPMI 1640 with FBS was added to the cells to give a final cell density of 3.3 × 10^5^ cells/ml. The cells were harvested at 48 h by centrifugation at 2,000 × *g* for 5 min at 25 °C. Cell supernatants were removed and the cell pellets were washed twice with 50 mM HEPES, pH 7.0 then further analyzed for percent infection by flow cytometry. The antibody dilution that produced the highest percentage of DENV 2 infection was chosen for large scale preparation.

To prepare DENV 2 infected cells for phosphoproteomic analysis and western blots, large scale ADE infection was performed with an approximately 30-fold increase in scale as compared to the optimization experiment. DENV 2 immune complexes were set up in triplicate in 60 mm tissue culture dishes by adding 20 MOI of DENV 2 virus and HB114 antibody at a dilution of 1:200 in RPMI 1640. The complex was incubated at 4 °C for 1 h with gentle rocking every 20 min. A total of 3.3 × 10^7^ U937 cells in RPMI with no FBS were seeded into 150 mm cell culture dishes. Then the immune complexes were added to the cells. The culture dishes were incubated for 2 h at 37 °C with 5% CO_2_ with rocking the culture dishes every 30 min. Complete RPMI 1640 was added to the cells to give a final cell density of 3.3 × 10^5^ cells/ml equal to the cell density in the optimization experiment. The cells and the culture supernatant were harvested at 48 h by centrifugation at 2,000 × *g* for 5 min at 25 °C. After washing twice, approximately 3 × 10^6^ cells were transferred into a new 1.5 ml tube for percentage infection cell analysis and percent cell survival by flow cytometry and trypan blue, respectively. The remaining cells were aliquoted into 3 × 10^6^ cells/tube and stored at − 70 °C for further experiments. Cell culture supernatants were aliquoted as 1 ml/tube and also stored at − 70 °C.

### Quantification of DENV 2 infected U937 cells by flow cytometry

Quantification of infection was undertaken by flow cytometry exactly as previously described^22^, except that analysis was undertaken on a Cyan ADP 9-color flow cytometer (Beckman Coulter, Brea, CA) and analysis was performed using Kaluza software (Beckman Coulter, Brea, CA). All experiments were undertaken independently in duplicate. Infected cells were gated as M2.

### Phosphoprotein preparation for 2-D electrophoresis

A Pierce phosphoprotein enrichment kit (Thermo Fisher Scientific Inc., Waltham, MA) was employed for the enrichment of phosphoproteins. Briefly, U937 cell pellets were resuspended in lysis/binding/wash buffer with CHAPS, 1X Halt protease inhibitor EDTA free (Thermo Fisher Scientific Inc., Waltham, MA) and 1X Halt phosphatase inhibitor cocktail (Thermo Fisher Scientific Inc., Waltham, MA). The cell suspensions were sonicated intermittently, centrifuged and the soluble protein fractions were collected. Approximately 3.7–4.0 mg of protein from each U937 cell lysate was applied to the phosphoprotein affinity column. The samples were incubated on the column for 30 min at 4 °C and washed with lysis/binding/wash buffer to remove non-specific binding proteins. Phosphoproteins were eluted with 5 ml of elution buffer (75 mM sodium phosphate, 500 mM sodium chloride; pH 7.5) and concentrated. The protein concentration for each sample was determined using the Bradford Protein assay (Bio-Rad Laboratories, Hercules, CA). Phosphoprotein-enriched samples were stored at − 80 °C until required.

### 2-D electrophoresis

Two-dimensional gel electrophoresis separation of 300 μg of enriched phosphoproteins was performed independently in triplicate as described previously^54^.

### Protein visualization

After separation, the gels were stained with Pro-Q Diamond phosphoprotein gel stain (Molecular Probes, Inc., Eugene, OR). Briefly, the gels were fixed in 50% methanol and 10% acetic acid and then washed with ultrapure water. The gels were stained in Pro-Q Diamond phosphoprotein gel stain with gentle agitation in the dark for 90 min. After staining, the gels were destained in 20% acetronitrile, 50 mM sodium acetate, pH4.0 for 1.5 h at room temperature and protected from light. The fluorescent spot images were acquired using a Typhoon Trio (GE Healthcare, Buckinghamshire, UK) with a 532 nm laser for excitation and a 580 nm filter for emission. Subsequently, the gels were washed and stained with SYPRO Ruby Protein gel stains (Bio-Rad Laboratories, Hercules, CA) overnight in the dark. The gels were washed with 10% methanol and 7% acetic acid. Fluorescence-stained proteins were then visualized using the scanner with a 532 nm laser and a 610 nm band pass filter. To cut out putative phosphoproteins for protein identification, all gels were stained with Coomasie G-250. The resulting spot pattern coincided with that of SYPRO Ruby staining therefore, the information from the Pro-Q Diamond/ SYPRO Ruby superimposed views were used to define the positions of the phosphoproteins in the Coomasie-stained gels.

### Protein identification and liquid chromatography-mass spectrometry analysis

Protein spots showing differential phosphorylation were removed and subjected to in-gel tryptic digestion and mass spectroscopic analysis essentially as described previously^54^. The MS/MS spectrometry data were searched against the NCBI database using the MASCOT search engine, as described elsewhere^54^. Functional analysis of identified phosphoproteins was performed using STRING (Search Tool for the Retrieval of Interacting Genes/Proteins) version 11.0.

### Western blotting for the detection of host phosphoproteins and DENV 2 proteins

U937 cell pellets from mock infection and DENV 2 infection were lysed with Lysis buffer (8 M urea, 2 M thiourea, 4% CHAP, 50 mM DTT and 1 mM PMSF). The cells were then sonicated using a sonicator with at an amplitude level of 6 for 5 s and pulsed twice for 10 s before centrifugation at 12,000 × *g* for 15 min at 4 °C. Then the cell lysates were transferred to new tubes. Proteins in the lysates were concentrated using a Viva-spin 2 ultrafiltration column with a 10 kDa molecular weight cut-off (GE Healthcare, Buckinghamshire, UK) in accordance with the manufacturer’s recommendations. The protein concentration was determined by the Bradford assay using a Bio-Rad protein assay kit (Bio-Rad, Hercules, CA) according to the manufacturers instructions. Approximately 10–20 µg of concentrated proteins were resolved by SDS-PAGE and proteins were transferred to PVDF membranes. Western blot analysis for detection of host phosphoproteins was carried out using the following antibodies: an anti-phospho-PKM2 (Tyr105) rabbit monoclonal antibody (Cell Signaling, USA), an anti-phospho-PKM2 (Ser37) rabbit polyclonal antibody (GeneTex, Irvine, CA), a rabbit polyclonal anti-PKM2 antibody (Abcam, Cambridge, UK) and an anti-GAPDH (14C10) rabbit monoclonal antibody (Cell Signaling Technology, Danvers, MA). For the detection of DENV proteins, antibodies used include an anti-envelope rabbit polyclonal antibody (GeneTex, Irvine, CA), an anti-NS1 mouse monoclonal antibody (R&D systems, Minneapolis, MN) and an anti-NS5 mouse monoclonal antibody (GeneTex, Irvine, CA). The secondary antibodies used were a horseradish peroxidase (HRP) conjugated goat anti-rabbit IgG (Santa Cruz Biotechnology, Dallas, TX) and an HRP conjugated rabbit anti-mouse IgG (Sigma-Aldrich, St. Louis, MO). ECL detection was performed according to the manufacturer’s protocol (Merck KGaA, Darmstadt, Germany). ImageJ software was employed to determine the optical density values of bands for relative comparisons. The experiments were performed independently in triplicate.

### Pyruvate kinase M2 inhibitor/activator treatment and cell viability assays

PKM2 inhibitor and PKM2 activator (Merck KGaA, Darmstadt, Germany) were made up as a 0.216 M and 0.268 M stocks in 100% DMSO, respectively. The PKM2 inhibitor was stored at − 20 °C for 2 weeks, whereas the PKM2 activator could be stored for 6 months. Both compounds were serially diluted to various concentrations using complete RPMI. The final concentration of DMSO was 0.1% for PKM2 inhibitor treatment and 0.01% for PKM2 activator treatment. Vehicle/RPMI used as a control of PKM2 inhibitor or PKM2 activator was 0.1% DMSO and 0.01% DMSO in RPMI, respectively.

To determine the IC_50_ for PKM2 inhibitor and PKM2 activator, U937 cells were incubated with various concentrations of each compound for 24 h and 48 h. before analysis of cell viability using the CellTiter 96 Aqueous One Solution Cell Proliferation assay (MTS, Promega, Madison, WI) according to the manufacturer's recommendations and measured with spectrophotometer (Spectra MR Microplate spectrophotometer, DYNEX Technologies, Chantilly, VA) at an absorbance value of 490 nm. IC_50_ curves were generated using GraphPad Prism version 5.0. Percent survival (Y axis) versus log concentration of inhibitor/activator (X axis) was plotted. The IC_50_ was calculated by the software. Each experiment was done independently in triplicate with duplicate analysis.

### Assay for virucidal activity

Stock DENV 2 was incubated in medium containing PKM2 inhibitor or PKM2 activator in a 37ºC water bath for 1 h prior to infection of LLC-MK2 cells after which the DENV 2 titer was determined by standard plaque assay. At least three independent measurements were collected to determine the mean and SEM values.

### Kinase inhibitor/activator post treatment of infected cells

In p.i. treatment studies, 100 μM PKM2 inhibitor or 20 μM PKM2 activator were added to mock or DENV 2 infected cells at 0, 3 and 24 h after infection. Cells were incubated under standard conditions until analyzed. Supernatant and cells were harvested at appropriate time points, and all experiments were performed independently in triplicate. Control experiments were undertaken using 0.1% and 0.01% DMSO for PKM2 inhibitor and PKM2 activator, respectively.

### Statistical analysis

The data are expressed as mean ± SEM. Proteome data analysis was undertaken using the Perseus software platform (https://www.perseus-framework.org) with One-sample T-test and Two-sample T-test analysis. All IC_50_, standard plaque assay, percent infected cells and western blot data were analyzed using the Graphpad Prism program version 5.0 (GraphPad Software Inc., San Diego, CA). Statistical analysis of significance was undertaken by unpaired t-test or One-Way ANOVA with Dunnett's Multiple Comparison Test including Bonferroni’s multiple comparison test. *P* values less than 0.05 were considered statistically significant.

## Data availability

All data generated or analysed during this study are included in this published article (and its Supplementary materials file).

## Supplementary information

Supplementary file1.
