# Supplementary material for: Phosphoproteomic analysis of dengue virus infected U937 cells and identification of pyruvate kinase M2 as a differentially phosphorylated phosphoprotein
Source: Sci Rep. 2020 Sep 2;10:14493. doi: 10.1038/s41598-020-71407-x (PMC7467932; doi:10.1038/s41598-020-71407-x)

Supplemental materials

Phosphoproteomic analysis of dengue virus infected U937 cells and identification of pyruvate kinase M2 as a differentially phosphorylated phosphoprotein

Jeerang Wongtrakul[1*], Thananya Thongtan[2], Supitcha Pannengpetch[3], Nitwara Wikan[4], Doungnapa Kantamala[1], Benjawan Kumrapich[1], Warissara Suwan[5], Duncan R. Smith[4*]

[1]Research Institute for Health Sciences, Chiang Mai University, 110 Intavaroros Road, Sriphum, Muang District, Chiang Mai 50200, Thailand;
[2]Department of Biochemistry, Faculty of Medicine, Chulalongkorn University, 1873 Rama 4 Road, Pathumwan, Bangkok 10330, Thailand;
[3]Center for Research and Innovation, Faculty of Medical Technology, Mahidol University, Bangkok, Thailand.
[4]Molecular Pathology Laboratory, Institute of Molecular Biosciences, Mahidol University, 25/25 Phuttamonthol Sai 4, Salaya, Nakorn Pathom 73170, Thailand;
[5]Department of Genetics, Faculty of Science, Maejo University, Chiang Mai, 50290, Thailand.

Co-corresponding authors:

Jeerang Wongtrakul, email: jeerang@gmail.com

or

Duncan R. Smith, email: duncan.smi@mahidol.ac.th

Supplementary Figures 1-10
Supplementary Table 1
Replicate 2D-gels
Uncropped western blots

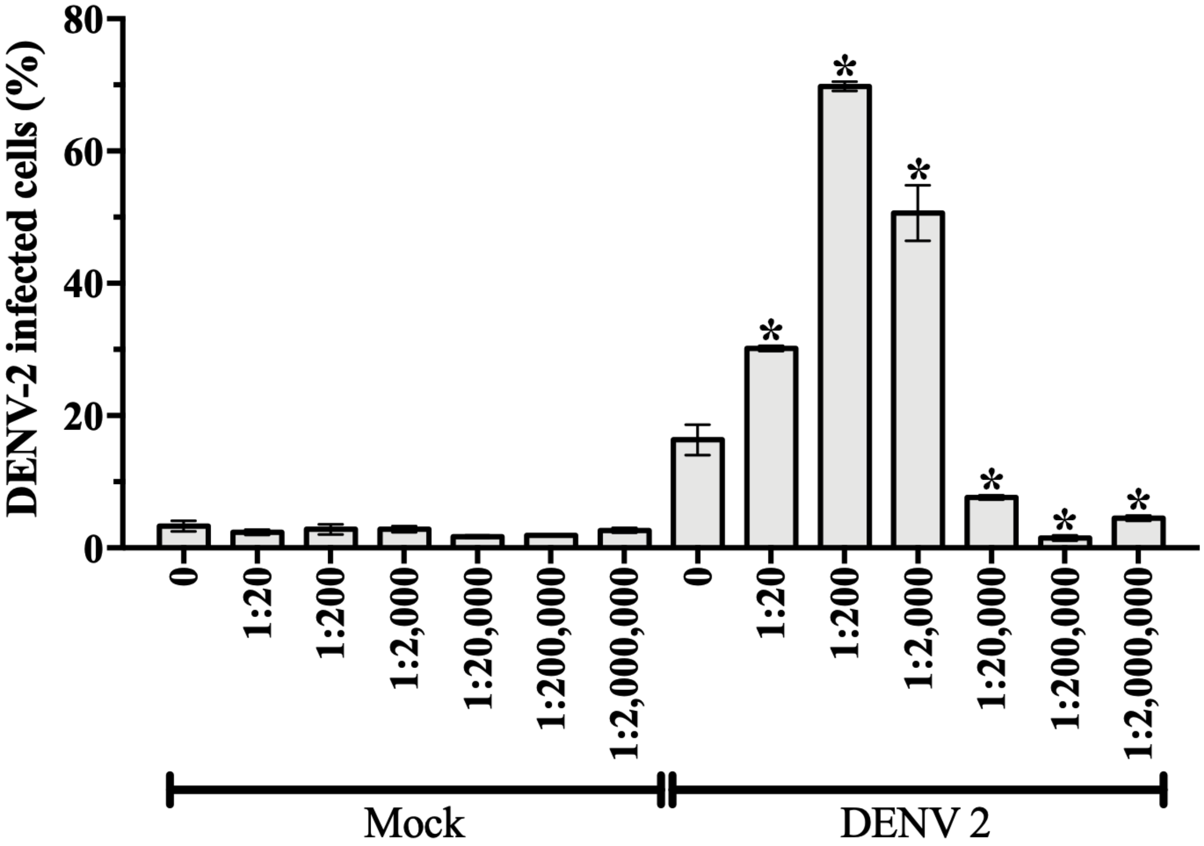

Supplementary Figure 1. DENV 2 infected antibody-mediated enhancement of infection in U937 cells. DENV 2 at MOI 20 was incubated with 10-fold serial dilutions of HB114 antibody in comparing with mock uninfected negative control antibody. Similarly, DENV 2 at MOI 20 in the absence of antibody was used as direct infection control. Percent infected cells were determined by flow cytometry. Results are demonstrated from three independent experiments as mean ± standard error of the mean (mean±SEM). Data were analyzed by One-way ANOVA; *p value< 0.05.

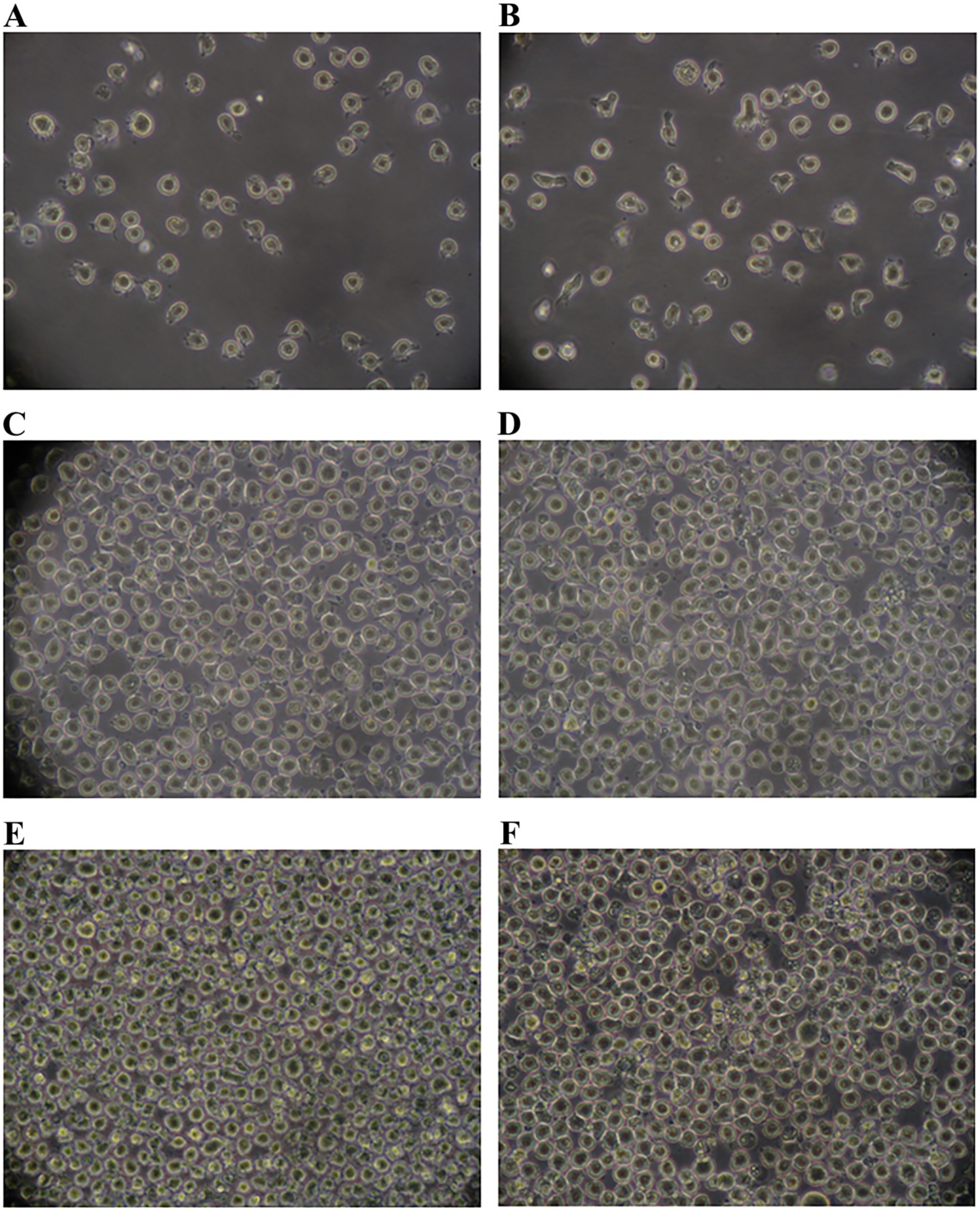

Supplementary Figure 2. Morphology of mock infected or DENV 2 infected U937 cells. Morphology of mock infected (A, C, E) or DENV 2 infected (B, D, F) U937 cells observed on day 0 (A, B), day 1 (C, D) or day 2 (E, F) post infection.

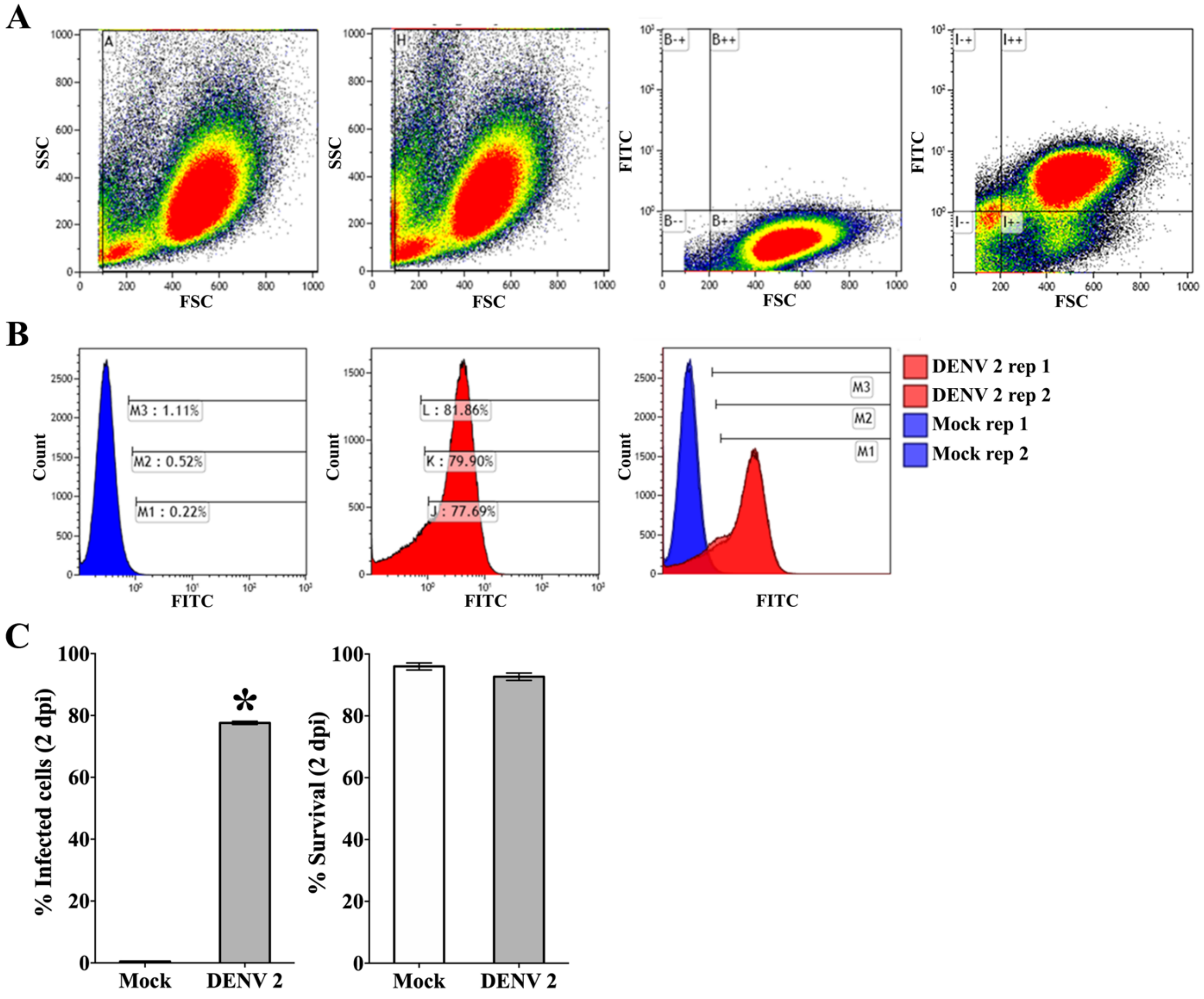

Supplementary Figure 3. DENV 2 infection of U937 cells. U937 cells were separately mocked infected or DENV 2 infected in the presence of a 1:200 final dilution of HB114 antibody and on day 2 p.i. The percentage of DENV infected cells was determined by flow cytometry and the percentage cell survival determined by a trypan blue dye assay. (A) Scatter plot of gated mock infected cells and DENV 2 infected cells, (B) Histogram plot of mock infected and DENV 2 infected cells, and (C) Quantification of DENV 2 infected cells using M2 population of each experiment and the percentage survival of cells. Data represent the means ± SEM of three independent experiments. Data were analyzed by an unpaired t-test; * p value< 0.05.

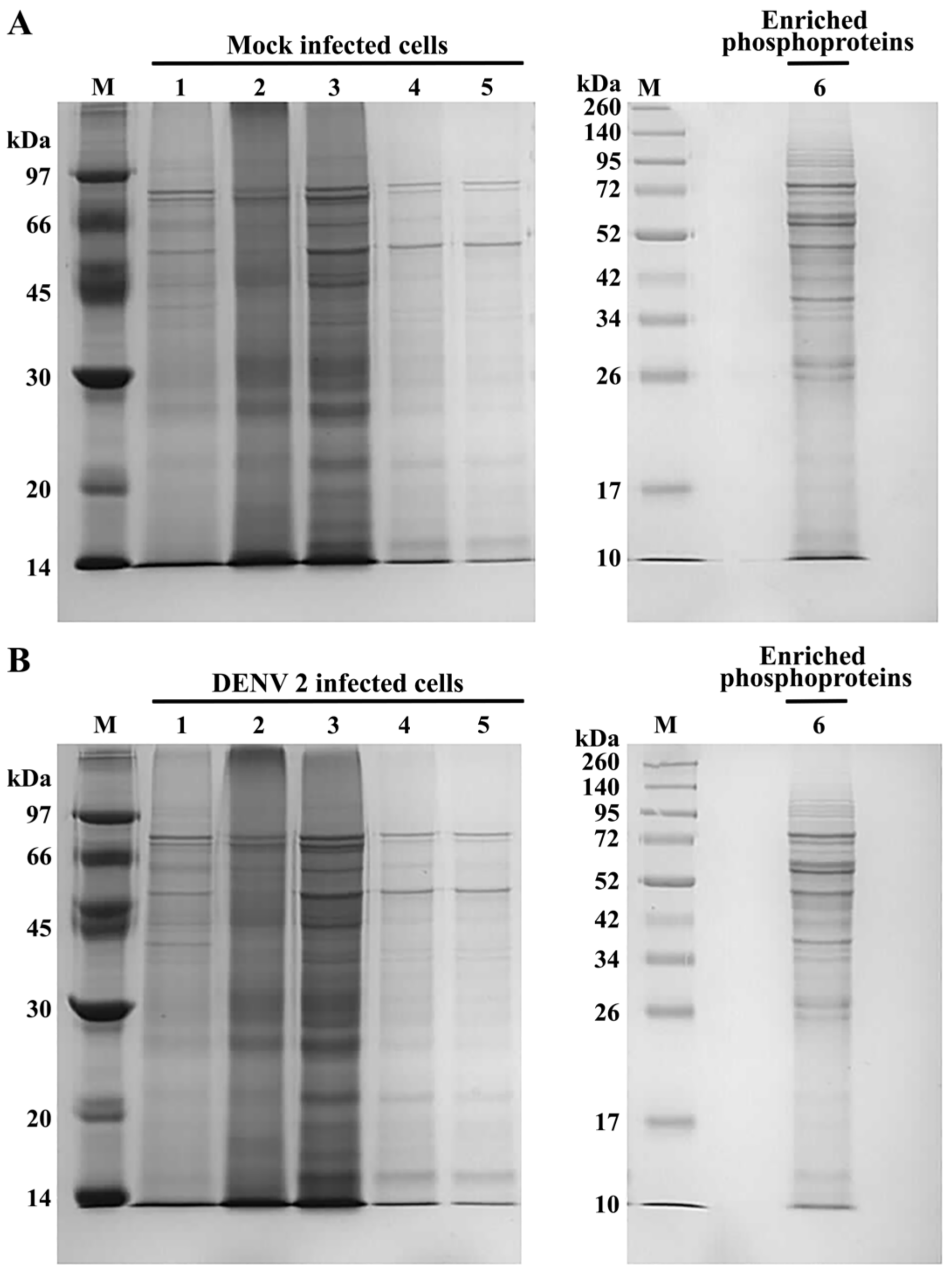

Supplementary Figure 4. Purification and enrichment of phosphoproteins from mock infected and DENV 2 infected U937 cells. (A) SDS-PAGE analysis of phosphoproteins purification from mock infected cells. Lane M, molecular weight marker, lane 1 cell lysate, lane 2 flow through, lane 3 wash fraction number 1, lane 4 wash fraction number 2, lane 5 wash fraction number 3, lane 6 enriched phosphoproteins. (B) SDS-PAGE analysis of phosphoproteins purification from DENV 2 infected cells. Lane M, molecular weight marker, lane 1 cell lysate, lane 2 flow through, lane 3 wash fraction number 1, lane 4 wash fraction number 2, lane 5 wash fraction number 3, lane 6 enriched phosphoproteins.

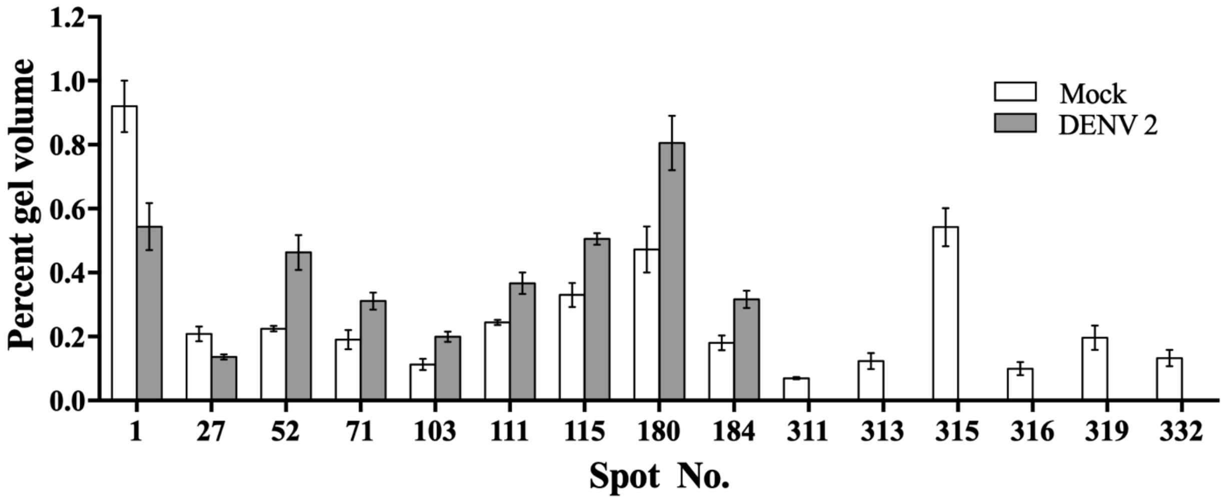

Supplementary Figure 5. The mean percent gel volumes of the 15 differentially phosphorylated proteins. The mean percent gel volumes of 15 differentially phosphorylated phosphoproteins of mock and DENV 2 infected cells are shown as mean ± SEM from triplicate gels.

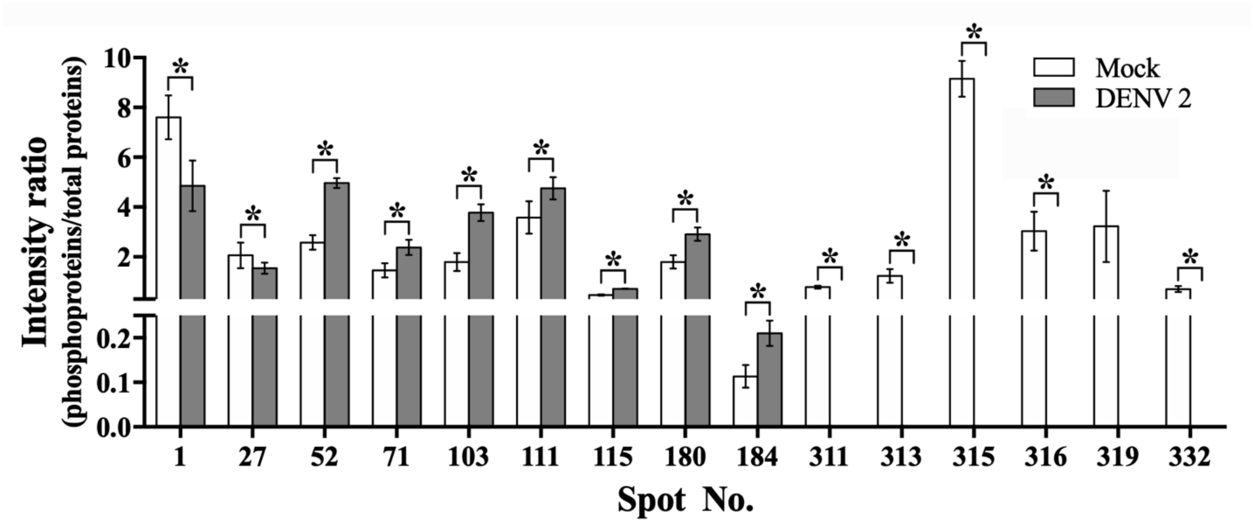

Supplementary Figure 6. Phosphoprotein-to protein intensity ratio of 15 differential expressed phosphoproteins. The ratio was calculated from the intensity of total protein spots and the phosphoprotein spots obtained from triplicate gels and shown as Mean ± SEM. Perseus program was employed to calculate statistical significant using One-sample test and Two-sample test (*; p value<0.05).

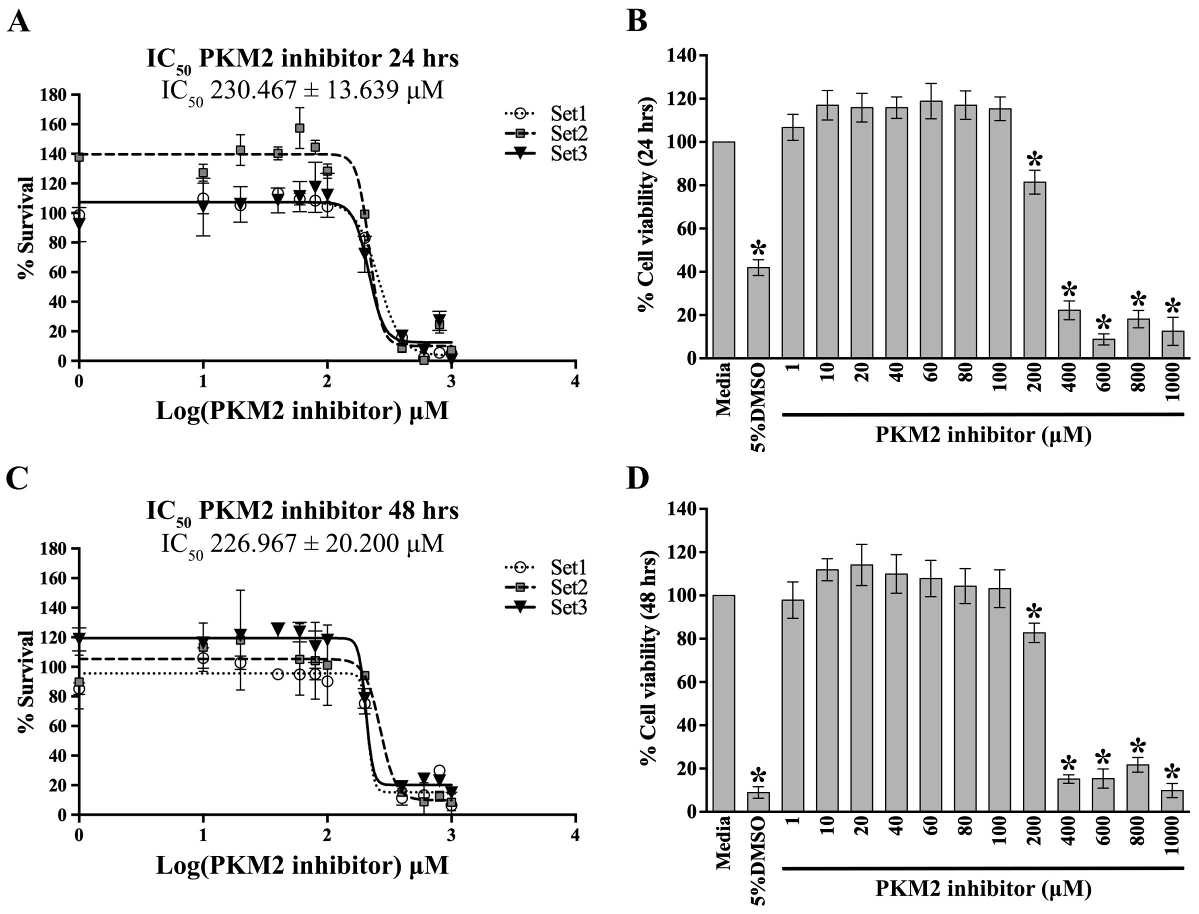

Supplementary Figure 7. Toxicity of a pyruvate kinase M2 inhibitor (PKM2 inhibitor) towards U937 cells. Determination of IC_{50} (A, C) and cell survival (B, D) of U973 cells exposed to a PKM2 inhibitor for 24 (A, B) or 48 h (C, D). Experiment was conducted independently in triplicate. Error bars show SEM. Data were analyzed by One-Way ANOVA with Bonferroni's multiple comparison test (*; *p* value<0.05).

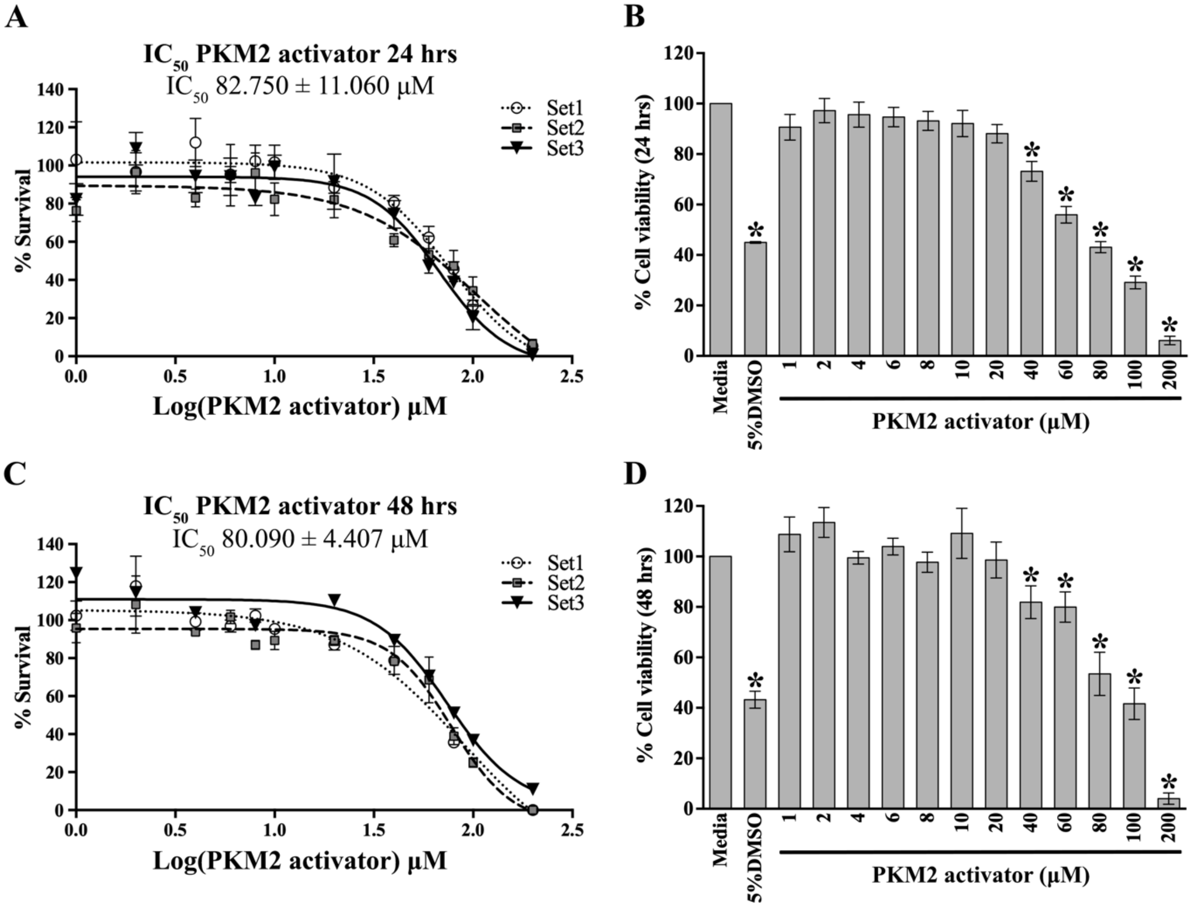

Supplementary Figure 8. Toxicity of pyruvate kinase M2 activator (PKM2 activator) towards U937 cells. Determination of IC_{50} (A, C) and cell survival (B, D) of U973 cells exposed to a PKM2 activator for 24 (A, B) or 48 h (C, D). Experiment was conducted independently in triplicate. Error bars show SEM. Data were analyzed by One-Way ANOVA with Bonferroni's multiple comparison test (*; p value<0.05).

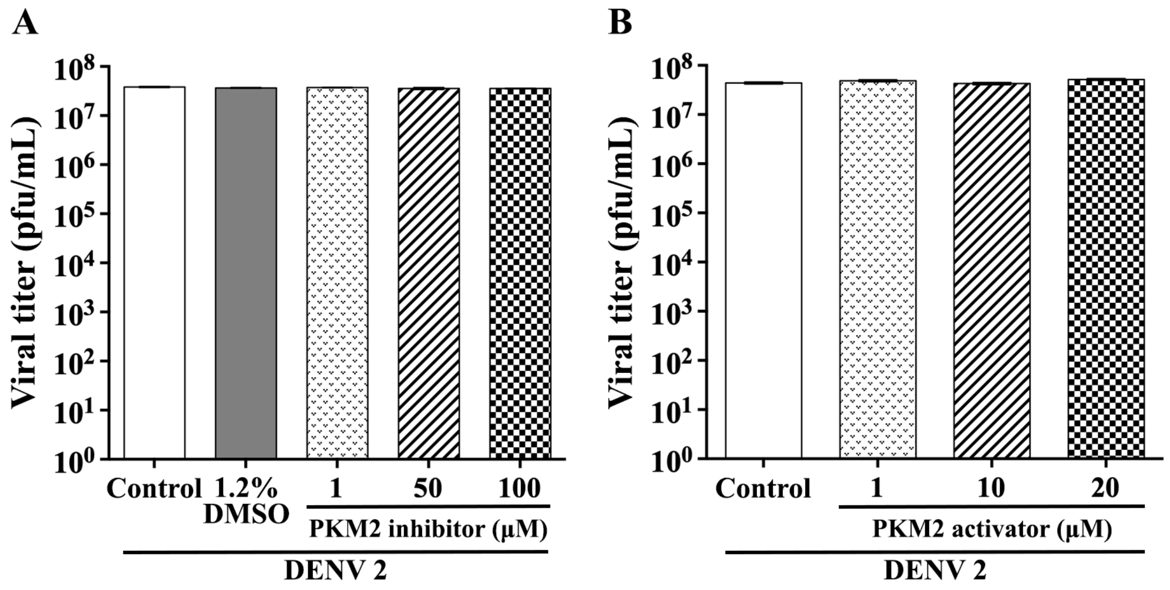

Supplementary Figure 9. Evaluation of virucidal activity of a pyruvate kinase M2 inhibitor and a pyruvate kinase M2 activator. Stock DENV 2 was incubated at 37°C for 1 hr with medium only, vehicle only or different concentrations of (A) a pyruvate kinase M2 inhibitor (PKM2 inhibitor) or (B) a pyruvate kinase M2 activator (PKM2 activator) before determination of viral titer by standard plaque assay. Experiment was undertaken independently in triplicate, with duplicate plaque assay. Bars show mean ± SEM.

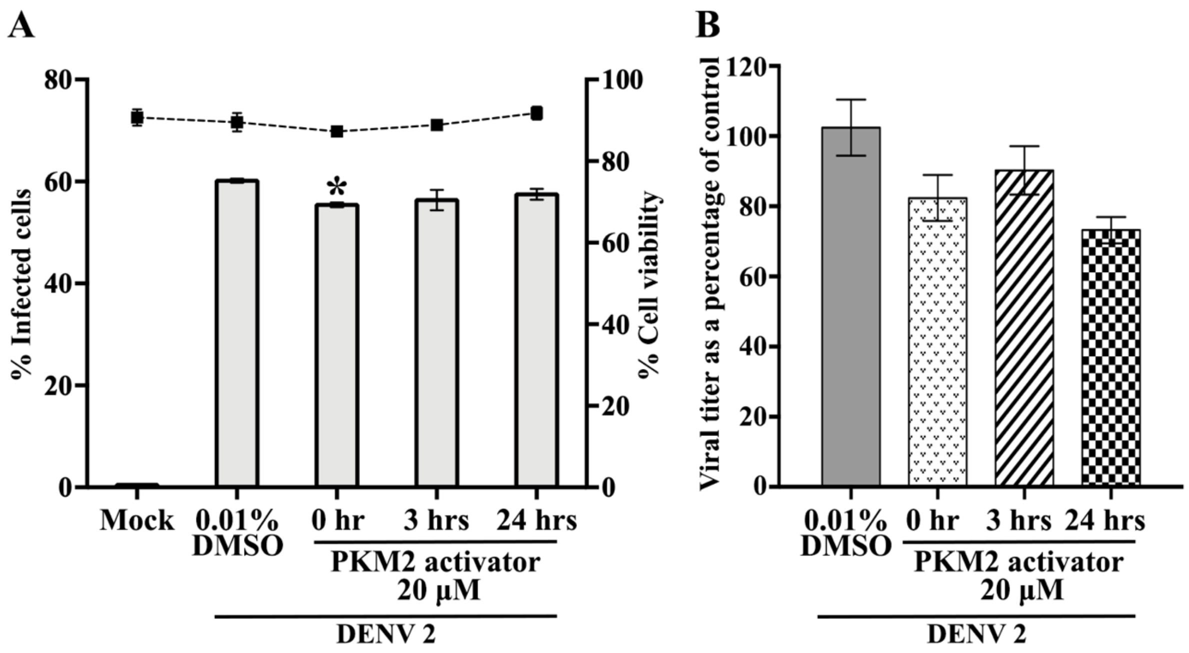

Supplementary Figure 10. Effect of post infection addition of pyruvate kinase M2 activator on DENV 2 infection. U937 cells were incubated with 20 μM PKM2 activator or with vehicle control at 0, 3 and 24 hours in parallel with a mock infection. (A) Cells were collected to determine the cell viability by trypan blue staining and infection level by flow cytometry, and (B) supernatants were collected to determine virus titer by standard plaque assay. Experiments were undertaken independently in triplicate with duplicate plaque assay. Bars show mean ± SEM (*; p value<0.05).

Supplementary Table 1. Functional enrichments in biological processes identified by the STRING bioinformatic analysis software.

**Biological Process (GO)**			
Pathway ID	Pathway description	Count in gene set	False Discovery Rate (FDR)
GO:0006091	generation of precursor metabolites and energy	11 of 388	8.45e-10
GO:0006123	mitochondrial electron transport, cytochrome c to oxygen	5 of 15	1.90e-08
GO:0046034	ATP metabolic process	8 of 190	2.48e-08
GO:1902600	proton transmembrane transport	7 of 137	6.46e-08
GO:0055114	oxidation-reduction process	12 of 923	6.46e-08
GO:0009167	purine ribonucleoside monophosphate metabolic process	8 of 230	6.46e-08
GO:0022900	electron transport chain	7 of 169	1.32e-07
GO:0055086	nucleobase-containing small molecule metabolic process	10 of 662	3.04e-07
GO:0072521	purine-containing compound metabolic process	9 of 478	3.19e-07
GO:0017144	drug metabolic process	9 of 622	2.55e-06
GO:0015980	energy derivation by oxidation of organic compounds	6 of 217	1.21e-05
GO:1901135	carbohydrate derivative metabolic process	10 of 1083	1.59e-05
GO:0019637	organophosphate metabolic process	9 of 1011	8.59e-05
GO:0006754	ATP biosynthetic process	4 of 75	9.32e-05
GO:0044281	small molecule metabolic process	11 of 1779	0.00014
GO:0006810	transport	16 of 4130	0.00014
GO:0072522	purine-containing compound biosynthetic process	5 of 206	0.00017
GO:0061718	glucose catabolic process to pyruvate	3 of 25	0.00017
GO:0061621	canonical glycolysis	3 of 25	0.00017

Biological Process (GO)			
Pathway ID	Pathway description	Count in gene set	False Discovery Rate (FDR)
GO:0006735	NADH regeneration	3 of 25	0.00017
GO:0009168	purine ribonucleoside monophosphate biosynthetic process	4 of 98	0.00019
GO:0010941	regulation of cell death	10 of 1638	0.00035
GO:0006757	ATP generation from ADP	3 of 39	0.00039
GO:0042866	pyruvate biosynthetic process	3 of 42	0.00047
GO:0001907	killing by symbiont of host cells	2 of 5	0.00058
GO:0034404	nucleobase-containing small molecule biosynthetic process	4 of 149	0.00068
GO:0046394	carboxylic acid biosynthetic process	5 of 311	0.00072
GO:0009435	NAD biosynthetic process	3 of 51	0.00072
GO:0006090	pyruvate metabolic process	3 of 66	0.0013
GO:0016310	phosphorylation	8 of 1236	0.0014
GO:1901137	carbohydrate derivative biosynthetic process	6 of 625	0.0015
GO:1901566	organonitrogen compound biosynthetic process	8 of 1370	0.0027
GO:0009166	nucleotide catabolic process	3 of 101	0.0037
GO:0034641	cellular nitrogen compound metabolic process	15 of 5126	0.0043
GO:0042981	regulation of apoptotic process	8 of 1501	0.0045
GO:0006139	nucleobase-containing compound metabolic process	14 of 4551	0.0047
GO:0006006	glucose metabolic process	3 of 113	0.0047
GO:0016051	carbohydrate biosynthetic process	3 of 120	0.0053
GO:1901564	organonitrogen compound metabolic process	15 of 5281	0.0056
GO:0019752	carboxylic acid metabolic process	6 of 854	0.0062

Biological Process (GO)			
Pathway ID	Pathway description	Count in gene set	False Discovery Rate (FDR)
GO:0043403	skeletal muscle tissue regeneration	2 of 29	0.0069
GO:0006796	phosphate-containing compound metabolic process	9 of 2065	0.0069
GO:0009987	cellular process	25 of 14652	0.0070
GO:0043900	regulation of multi-organism process	4 of 372	0.0110
GO:0050821	protein stabilization	3 of 166	0.0112
GO:0033574	response to testosterone	2 of 41	0.0120
GO:0071827	plasma lipoprotein particle organization	2 of 42	0.0124
GO:0015031	protein transport	7 of 1391	0.0124
GO:0008152	metabolic process	20 of 9569	0.0125
GO:0044237	cellular metabolic process	19 of 8797	0.0137
GO:0006094	gluconeogenesis	2 of 46	0.0142
GO:0035722	interleukin-12-mediated signaling pathway	2 of 47	0.0144
GO:0006457	protein folding	3 of 214	0.0197
GO:0005975	carbohydrate metabolic process	4 of 457	0.0197
GO:0051186	cofactor metabolic process	4 of 467	0.0209
GO:0006839	mitochondrial transport	3 of 223	0.0215
GO:0006807	nitrogen compound metabolic process	18 of 8349	0.0216
GO:0007569	cell aging	2 of 66	0.0248
GO:0006886	intracellular protein transport	5 of 836	0.0273
GO:0097006	regulation of plasma lipoprotein particle levels	2 of 70	0.0274
GO:0044238	primary metabolic process	18 of 8808	0.0419
GO:0046907	intracellular transport	6 of 1390	0.0487

Mock1 strip 18 cm. pH3-10 L load protein 300 ug

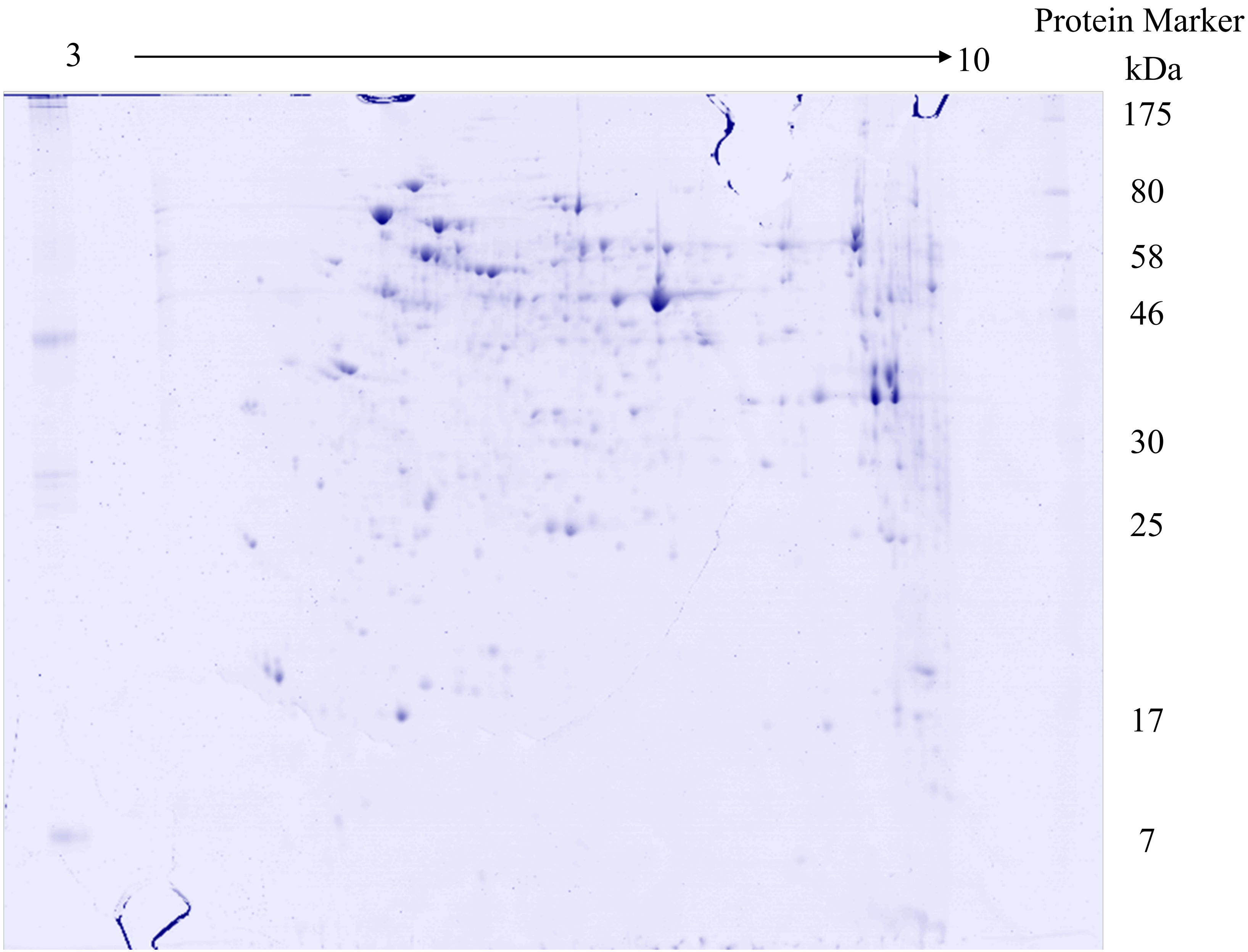

Mock2 strip 18 cm. pH3-10 L load protein 300 ug

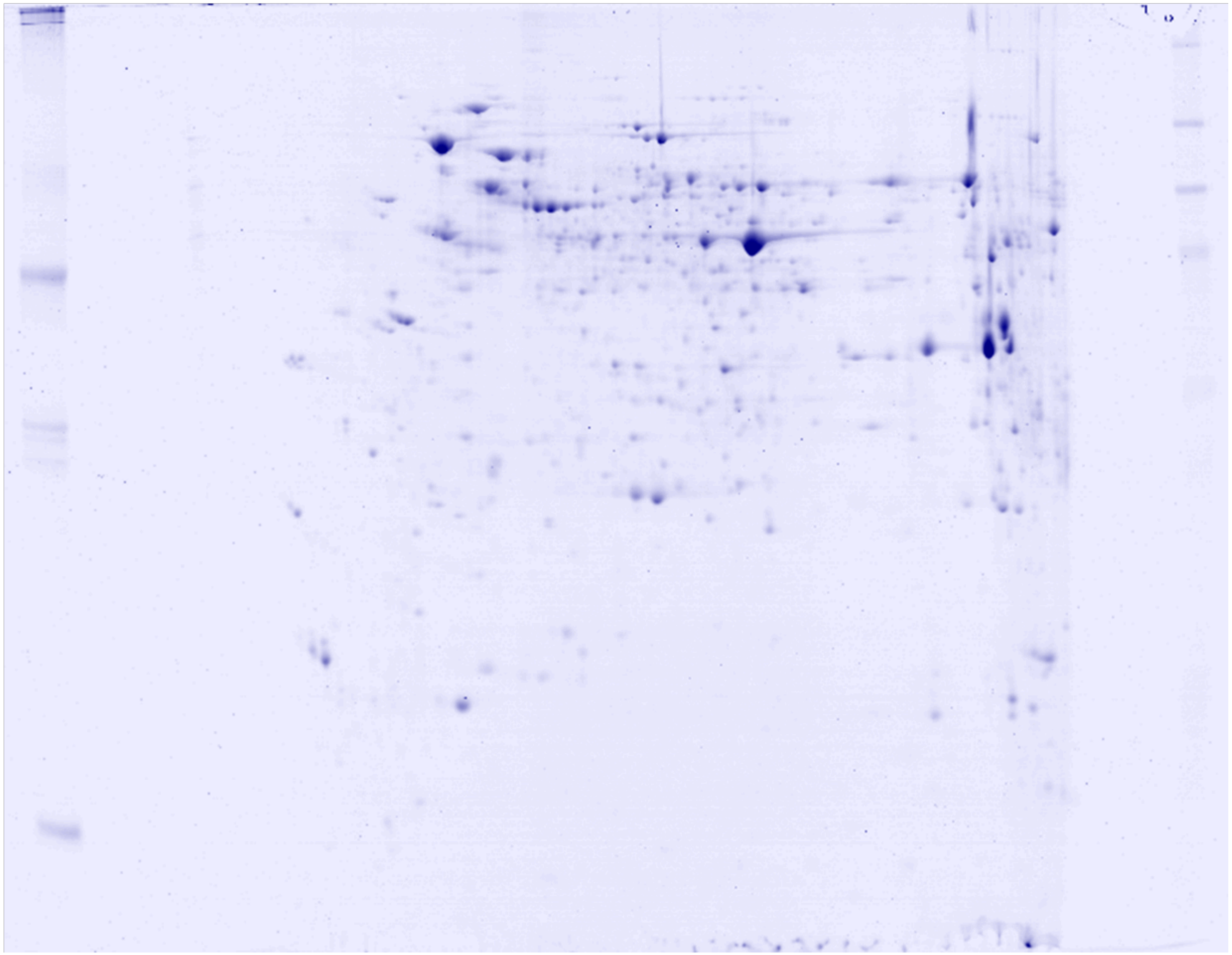

Mock3 strip 18 cm. pH3-10 L load protein 300 ug

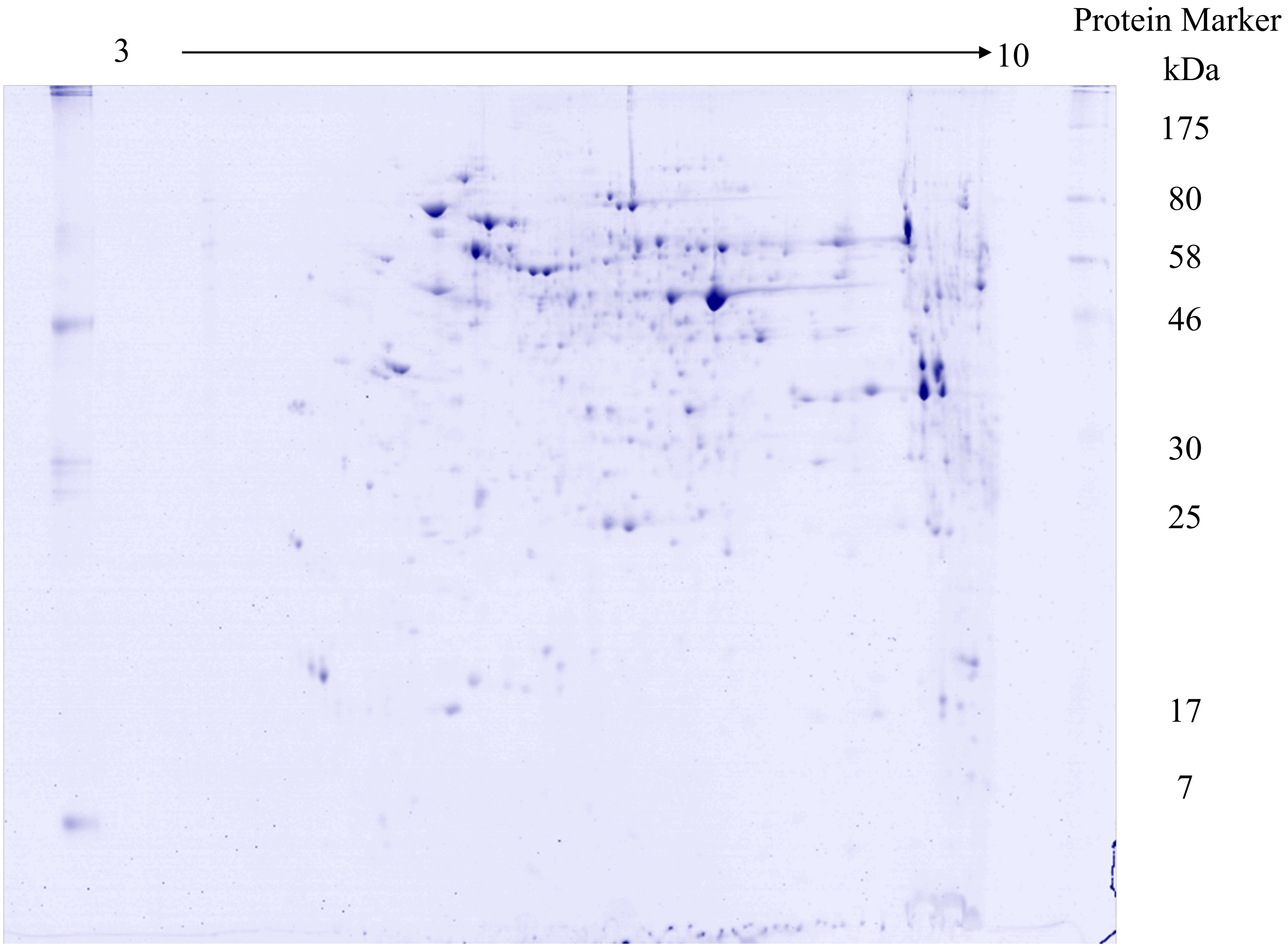

DV1 strip 18 cm. pH3-10 L load protein 300 ug

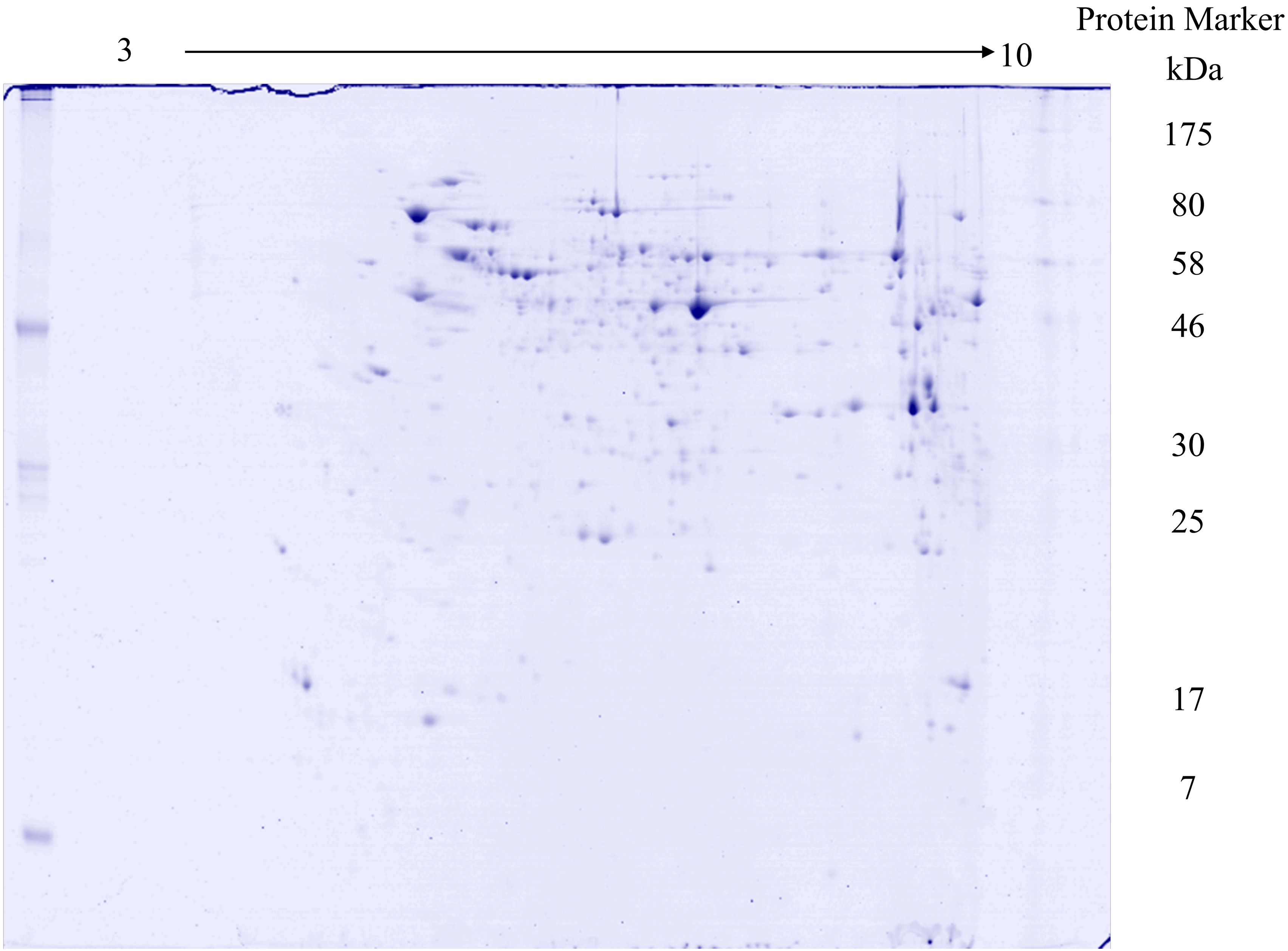

DV2 strip 18 cm. pH3-10 L load protein 300 ug

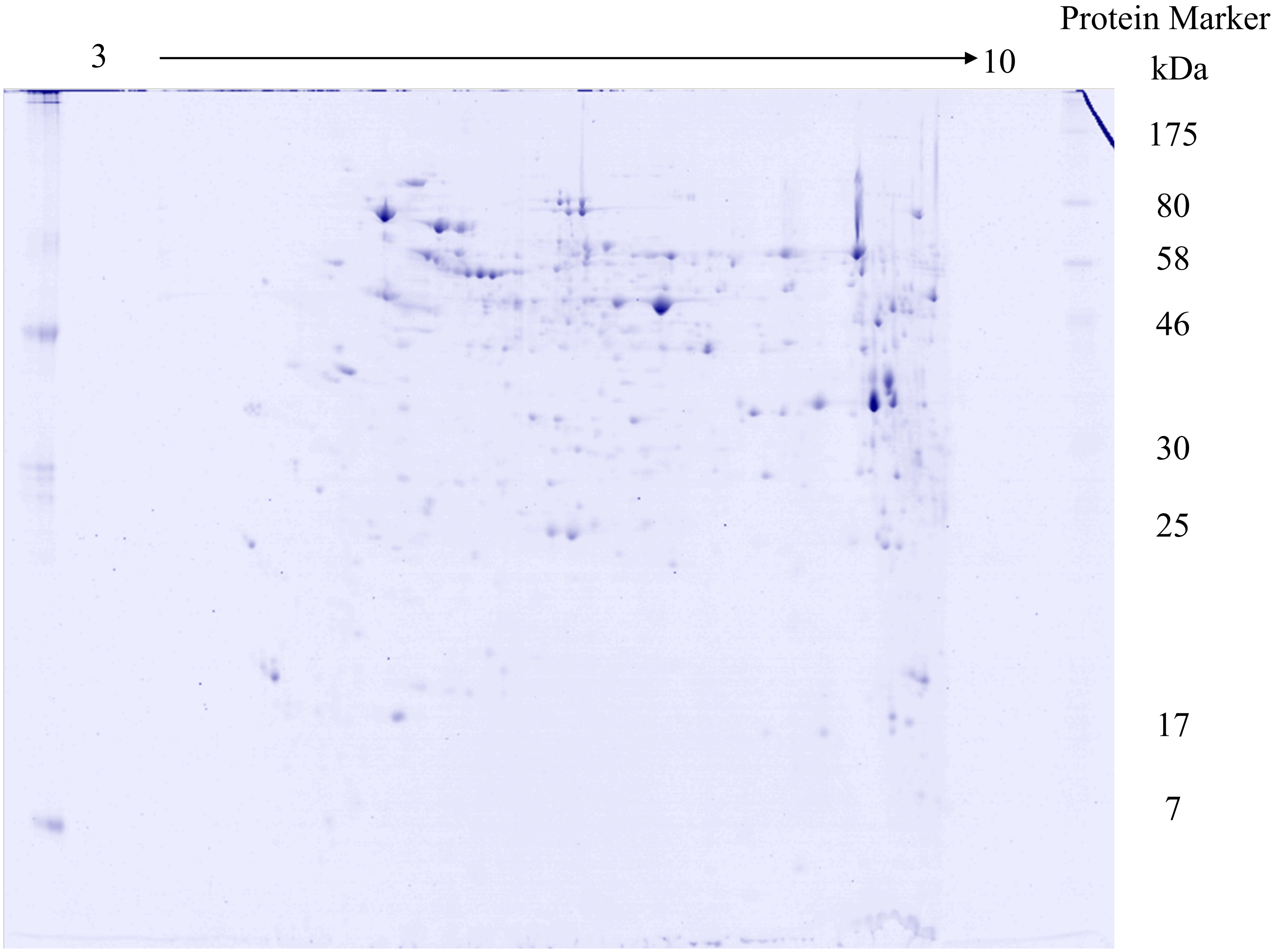

DV3 strip 18 cm. pH3-10 L load protein 300 ug

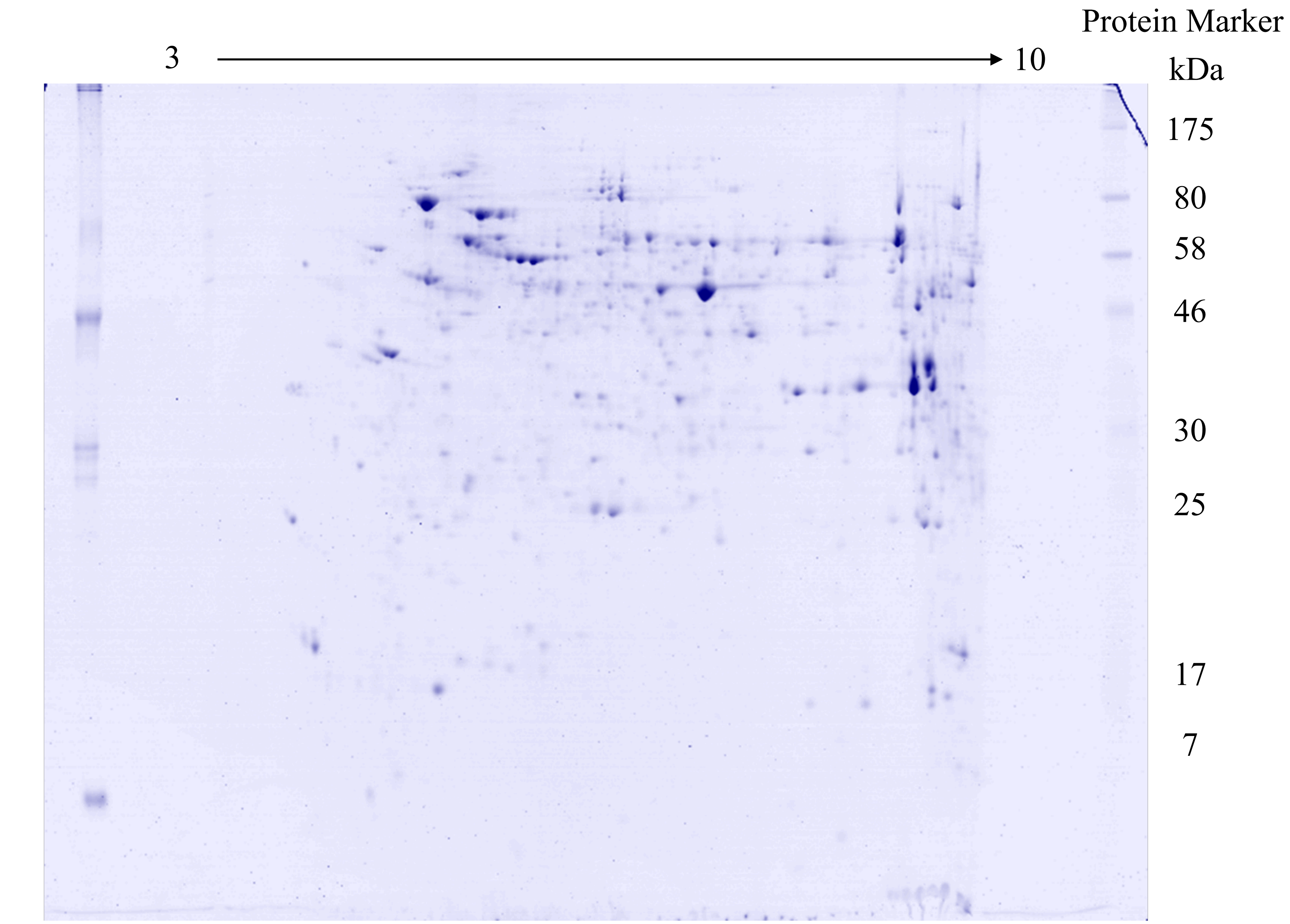

Mock1 strip 18 cm. pH3-10 L load protein 300 ug Pro-Q Diamond

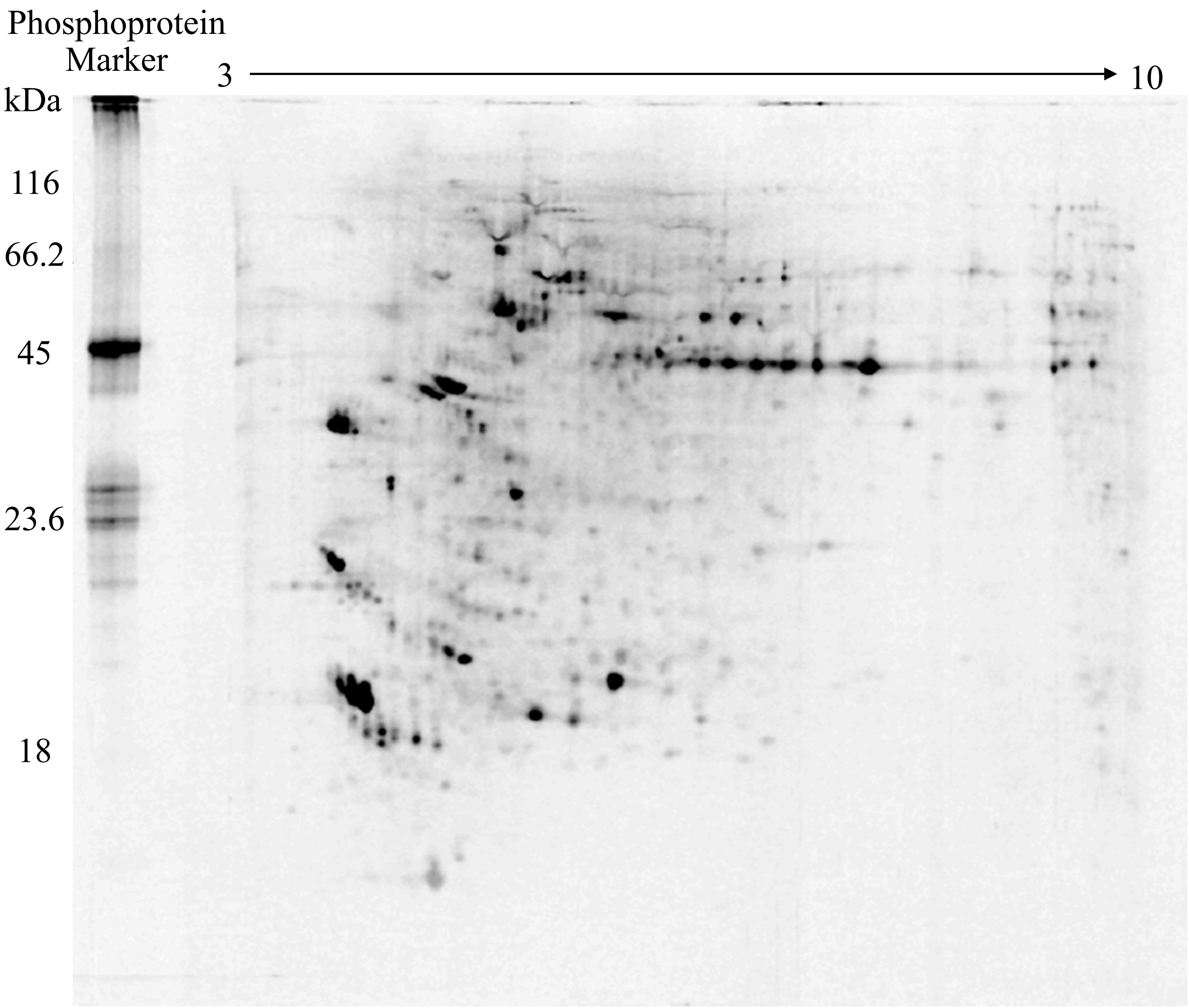

Mock2 strip 18 cm. pH3-10 L load protein 300 ug Pro-Q Diamond

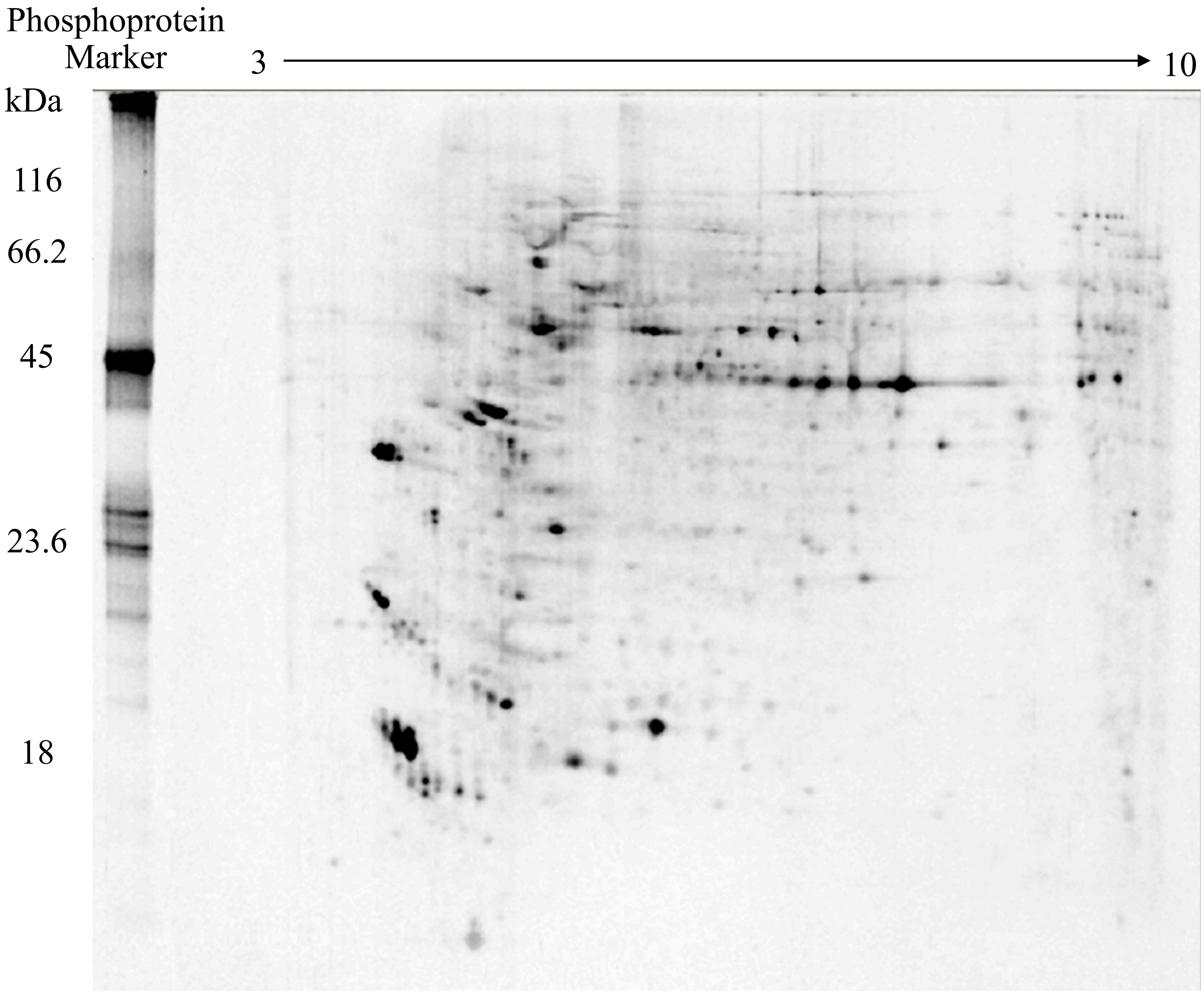

Mock3 strip 18 cm. pH3-10 L load protein 300 ug Pro-Q Diamond

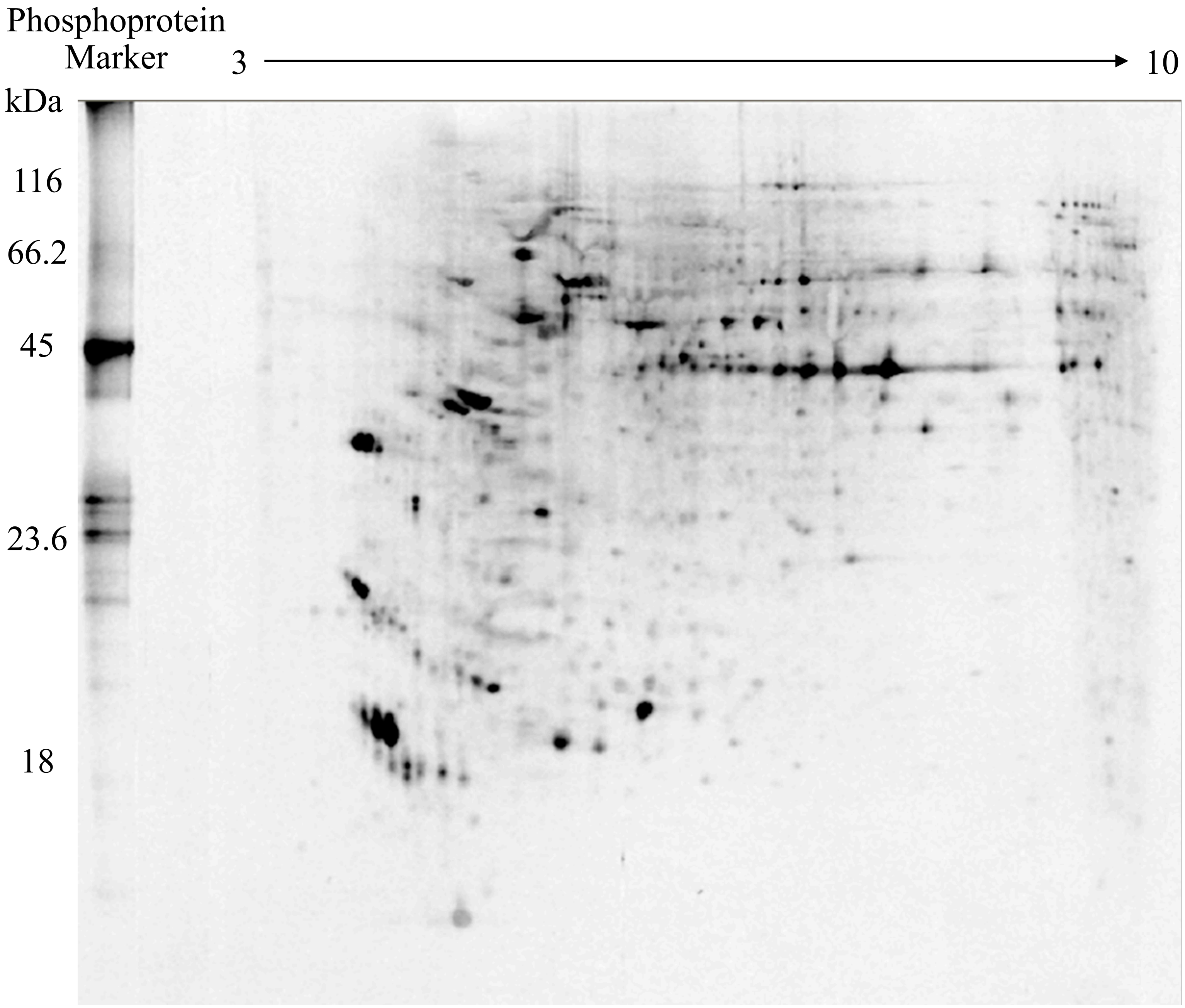

DV1 strip 18 cm. pH3-10 L load protein 300 ug Pro-Q Diamond

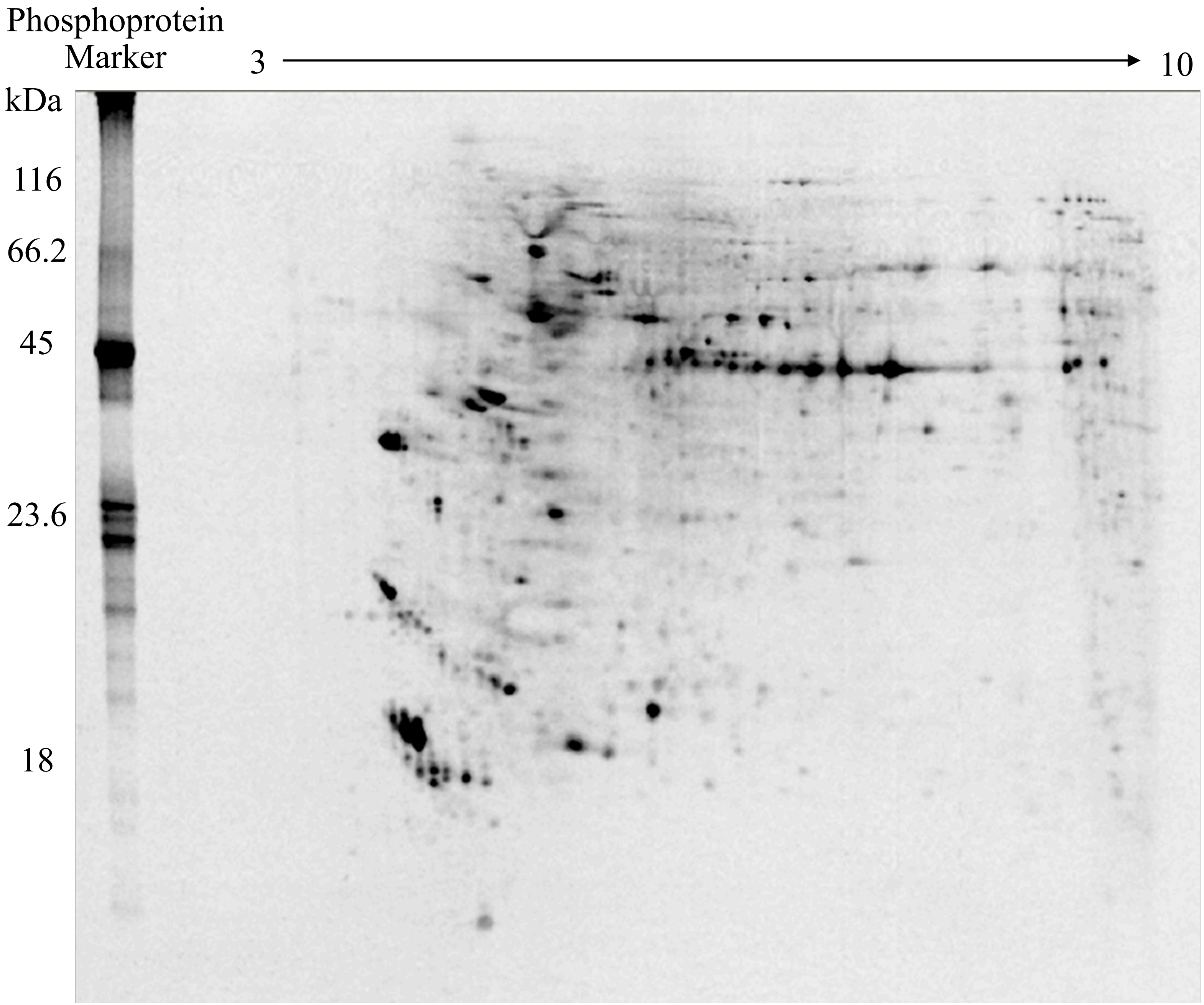

DV2 strip 18 cm. pH3-10 L load protein 300 ug Pro-Q Diamond

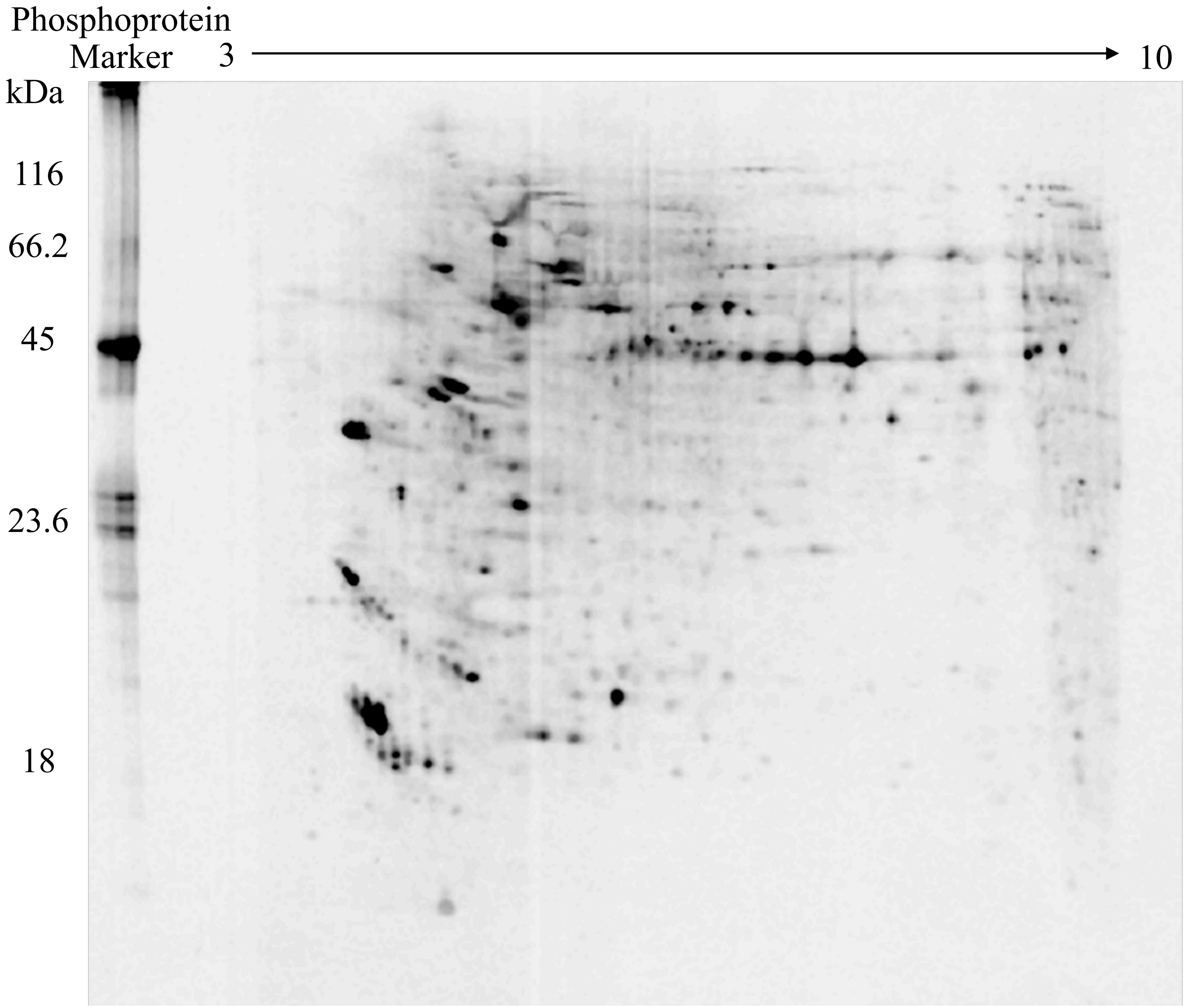

DV3 strip 18 cm. pH3-10 L load protein 300 ug Pro-Q Diamond

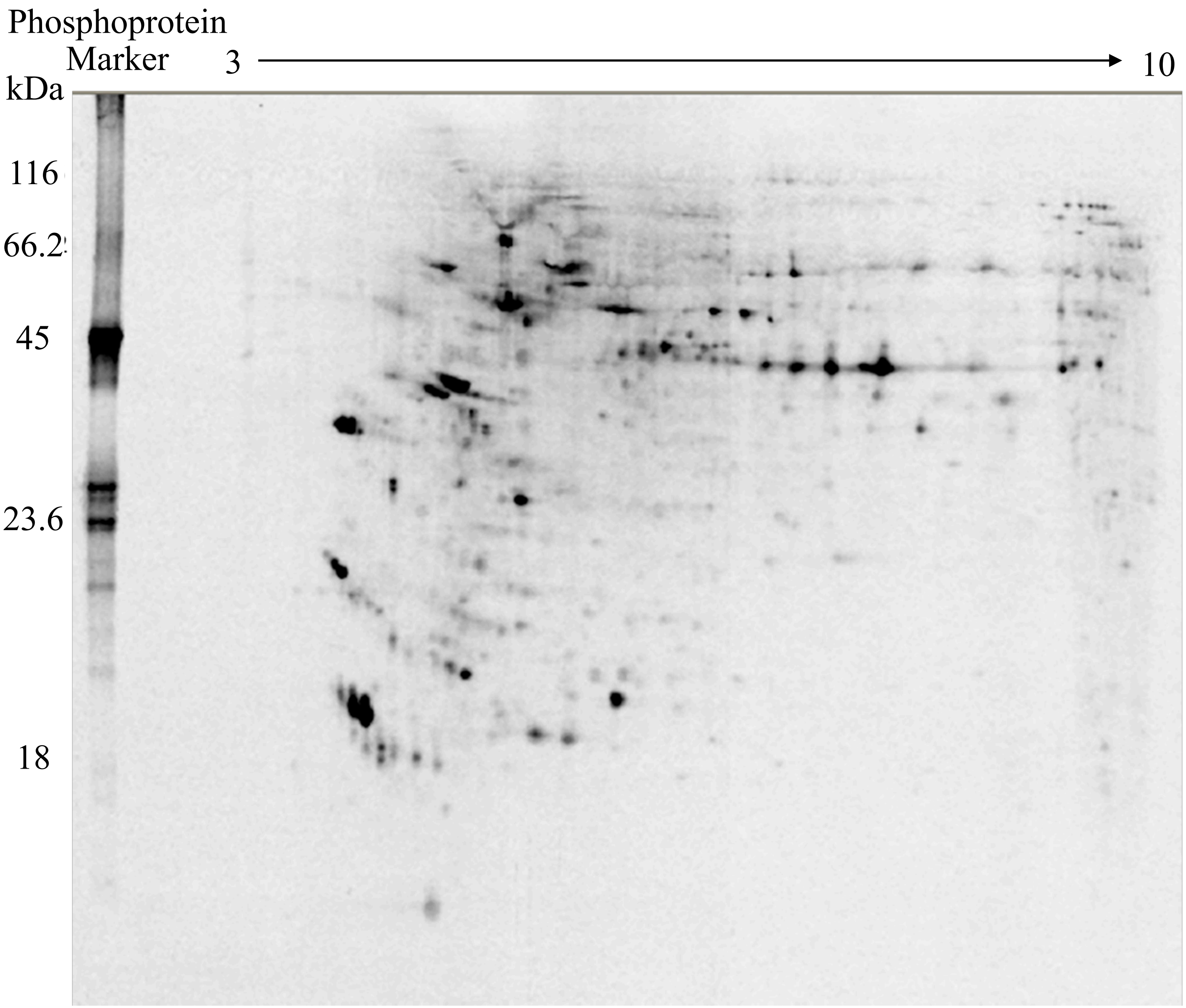

Mock1 strip 18 cm. pH3-10 L load protein 300 ug SyPro Ruby

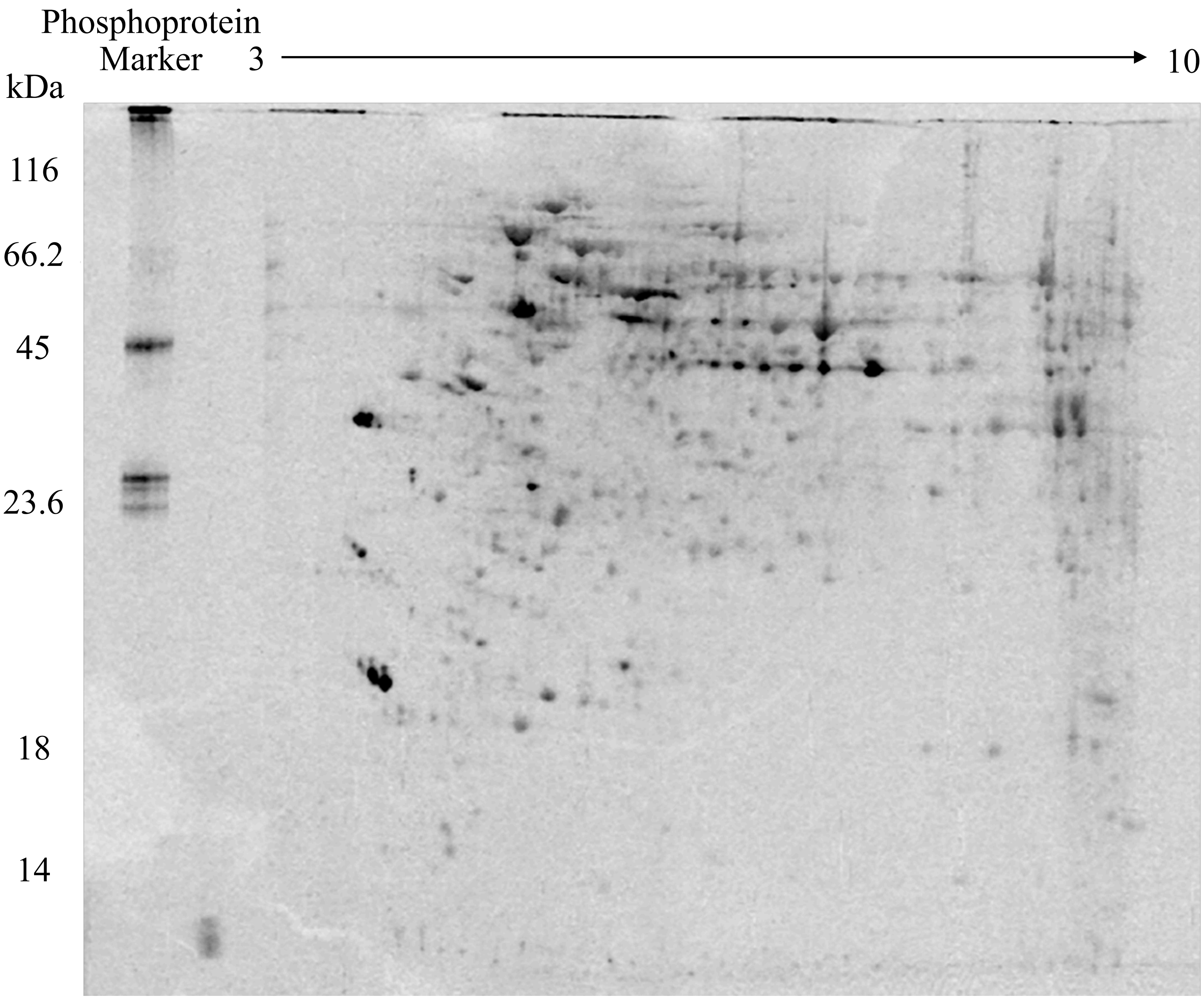

Mock2 strip 18 cm. pH3-10 L load protein 300 ug SyPro Ruby

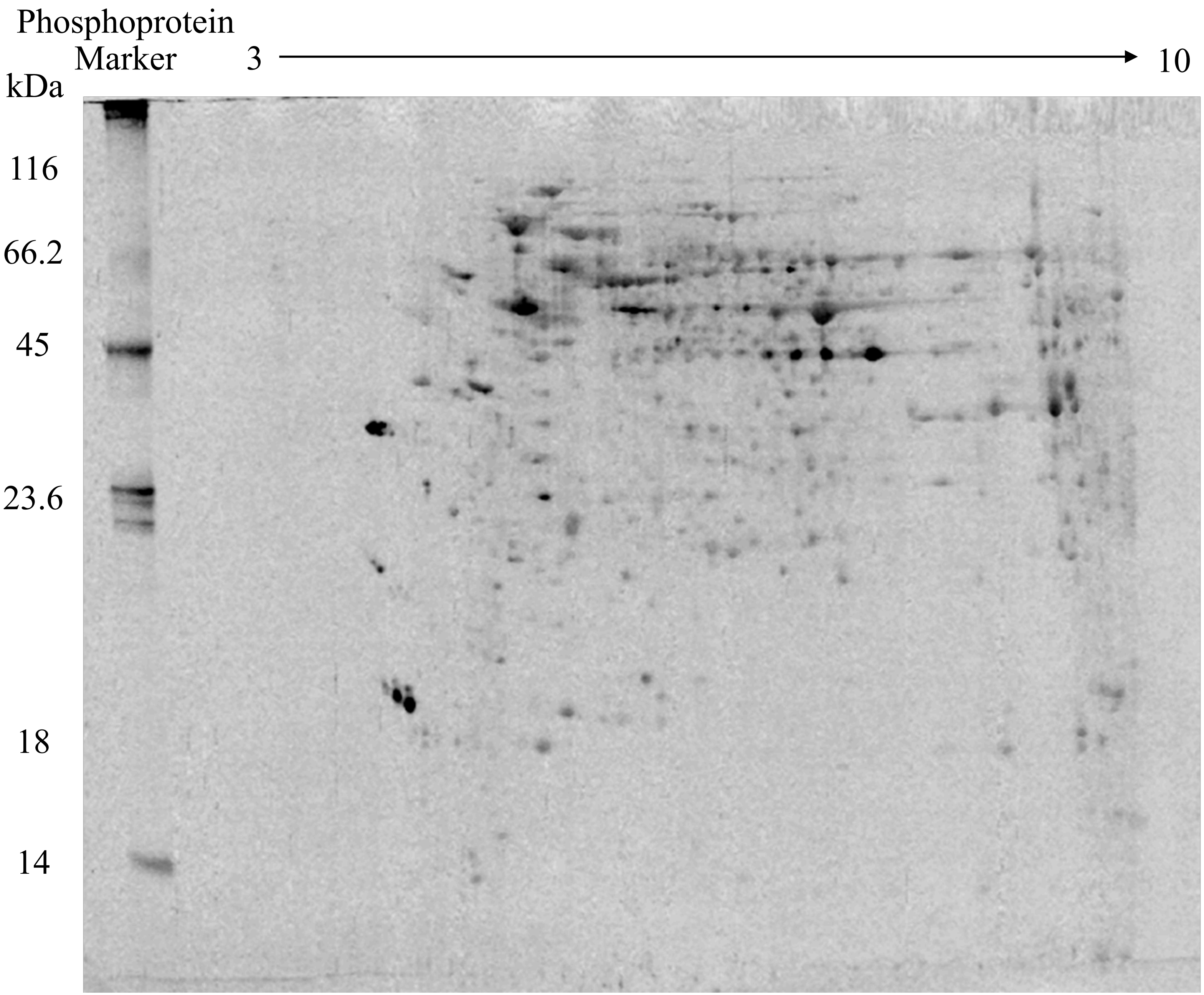

Mock3 strip 18 cm. pH3-10 L load protein 300 ug SyPro Ruby

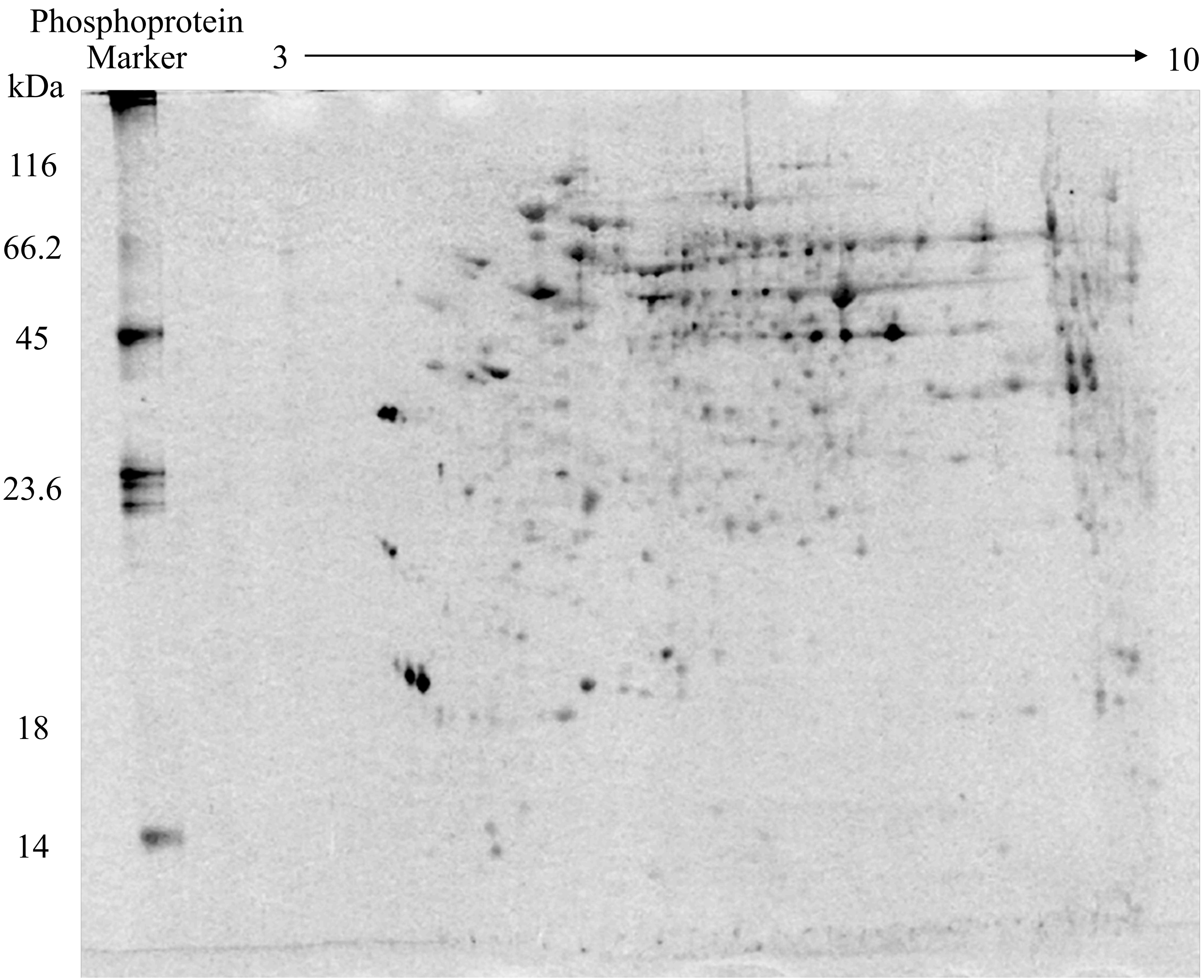

DV1 strip 18 cm. pH3-10 L load protein 300 ug SyPro Ruby

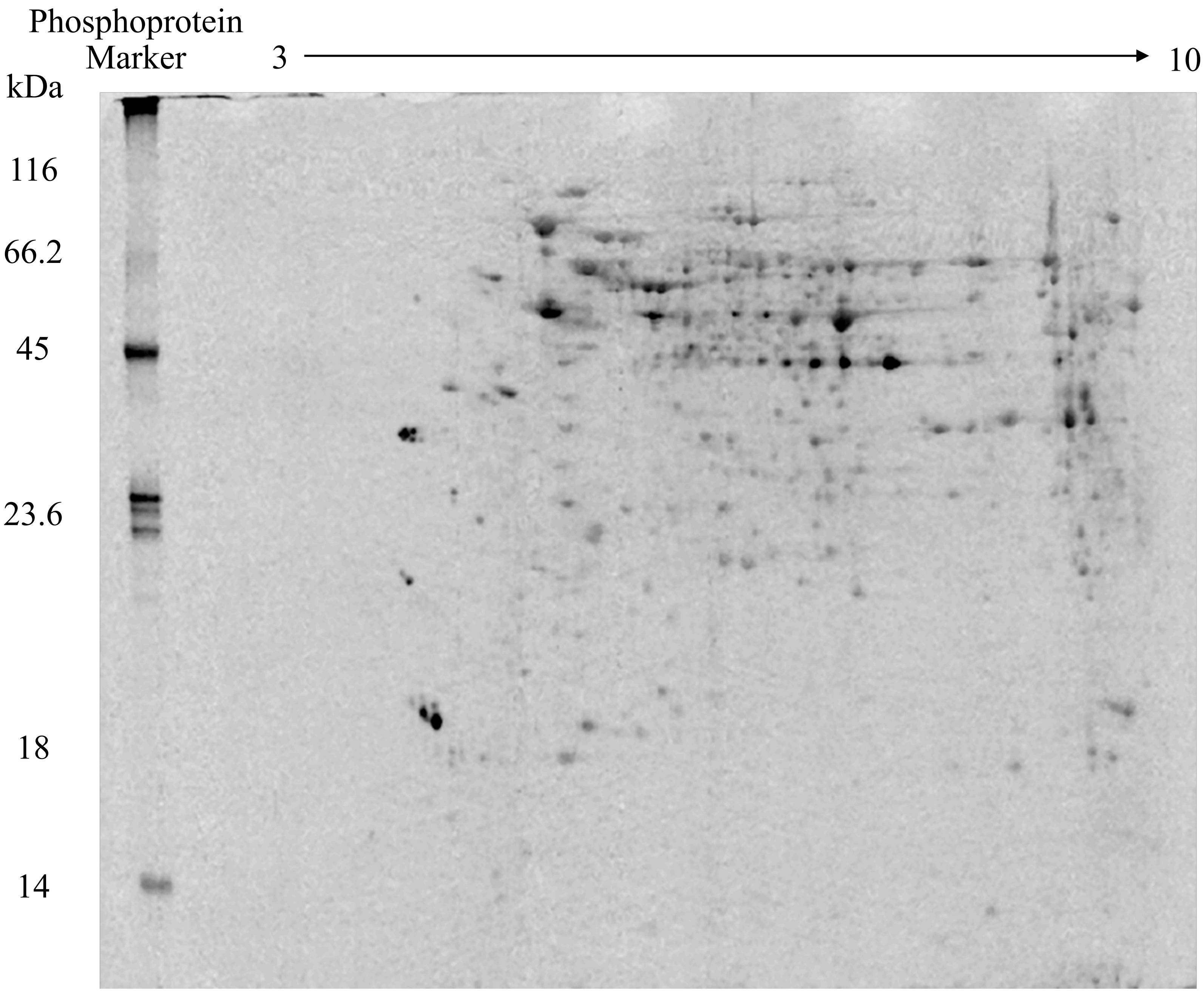

DV2 strip 18 cm. pH3-10 L load protein 300 ug SyPro Ruby

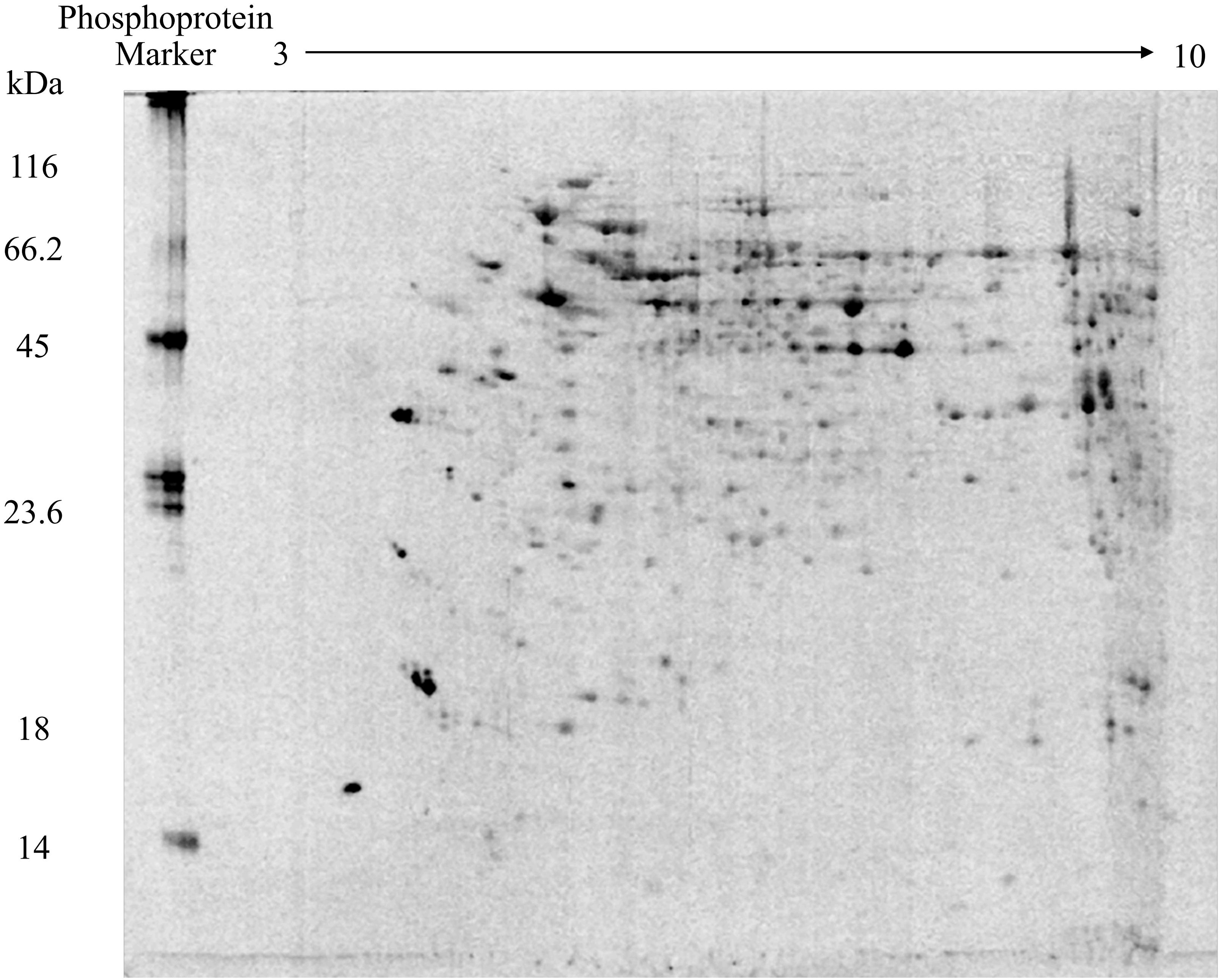

DV3 strip 18 cm. pH3-10 L load protein 300 ug SyPro Ruby

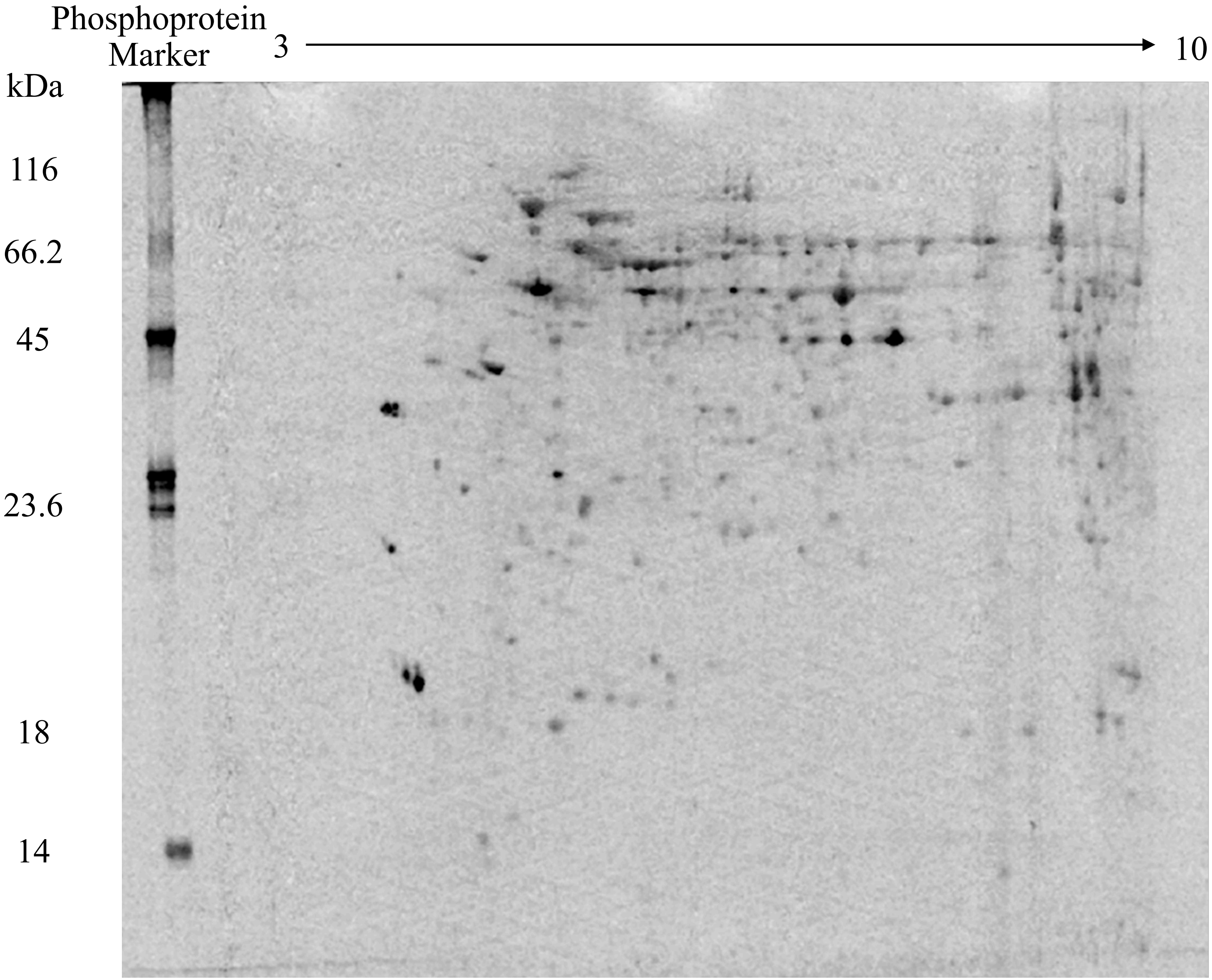

Mock_1 _ phosphoproteins

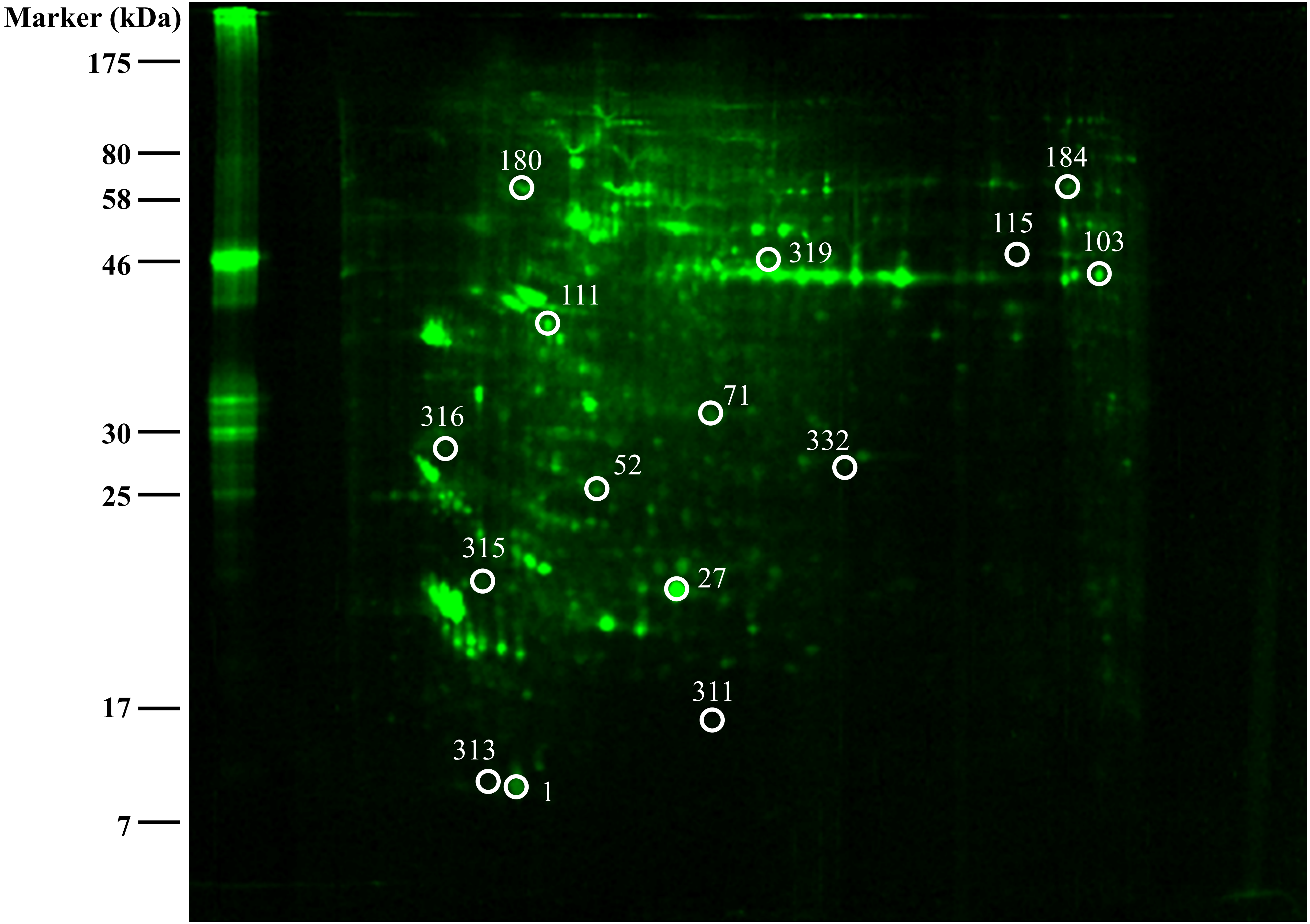

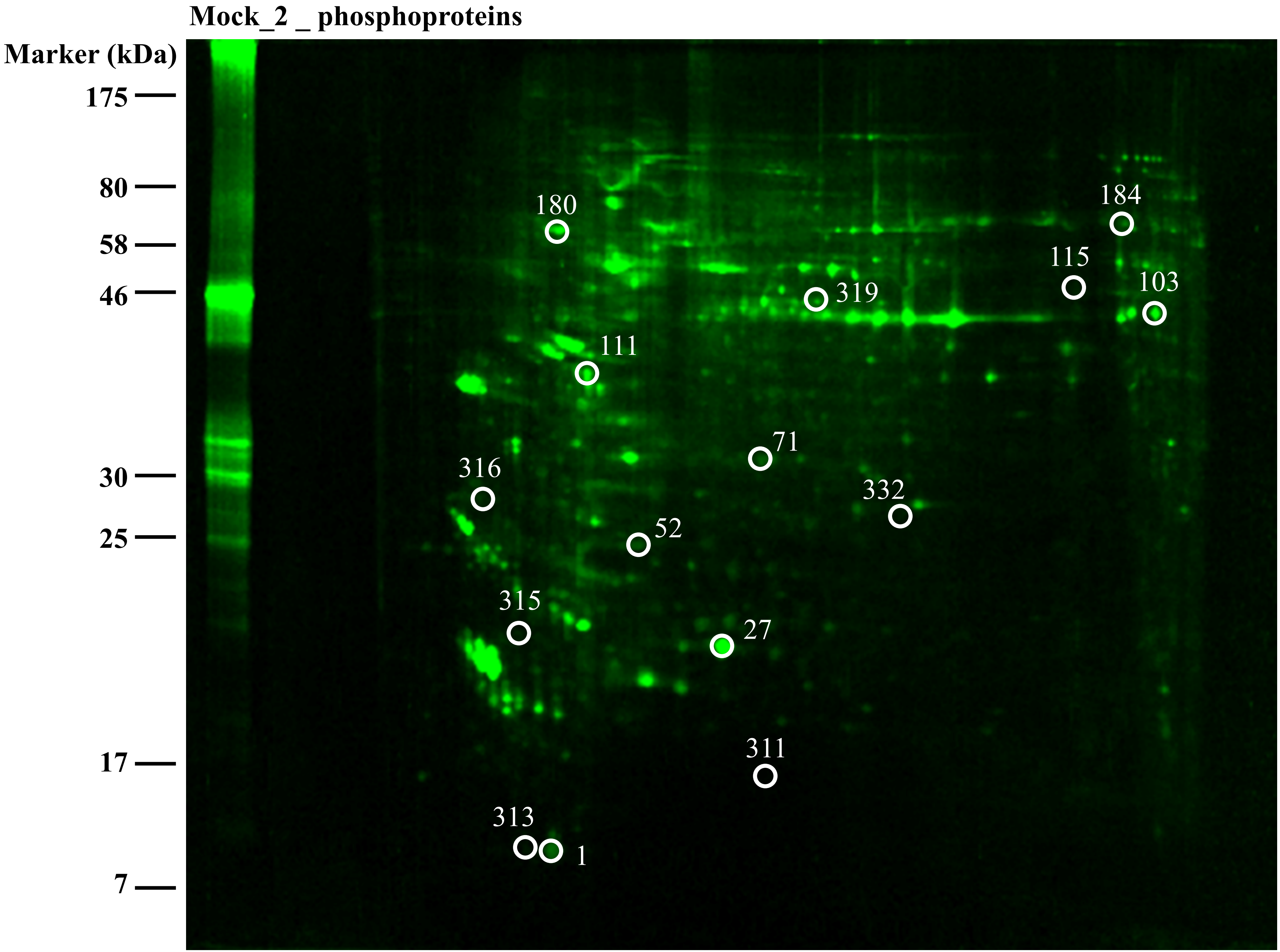
Mock_2 _ phosphoproteins
Marker (kDa)
175
80
58
46
30
25
17
7
180
184
115
103
319
111
71
316
332
52
315
27
311
313
1

Mock_3 _ phosphoproteins

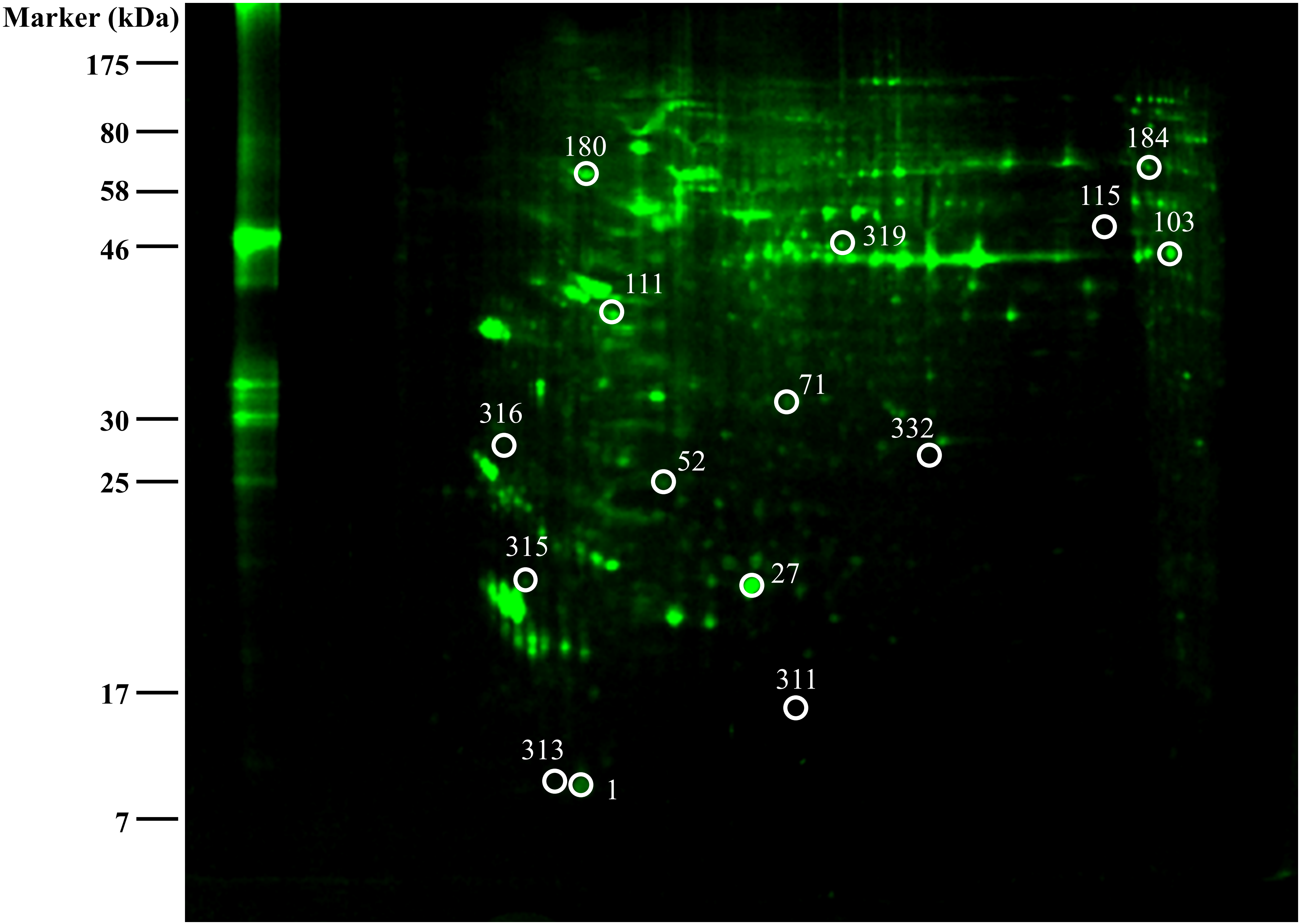

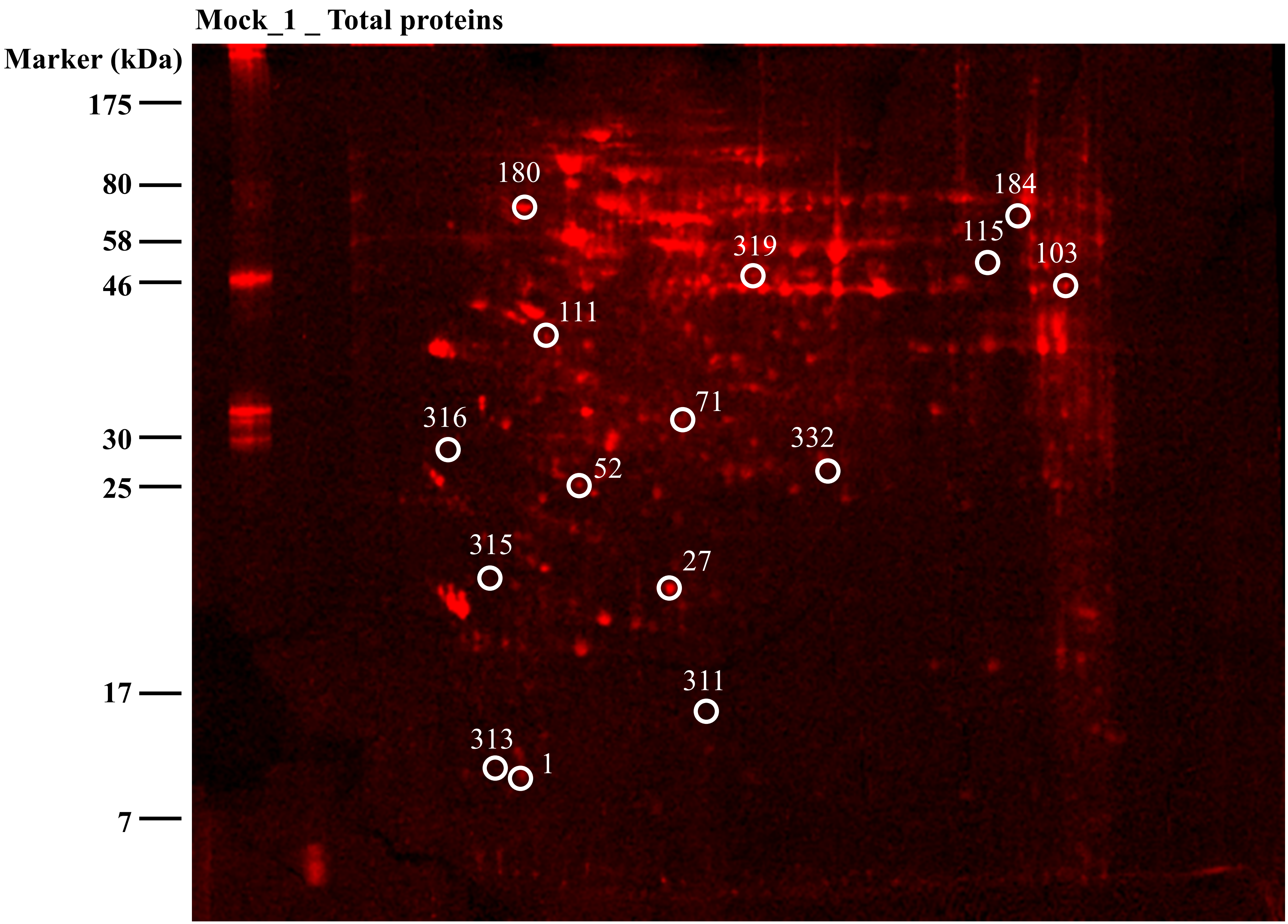
Mock_1 _ Total proteins
Marker (kDa)
175
80
58
46
30
25
17
7
180
184
115
319
103
111
71
316
332
52
315
27
311
313
1

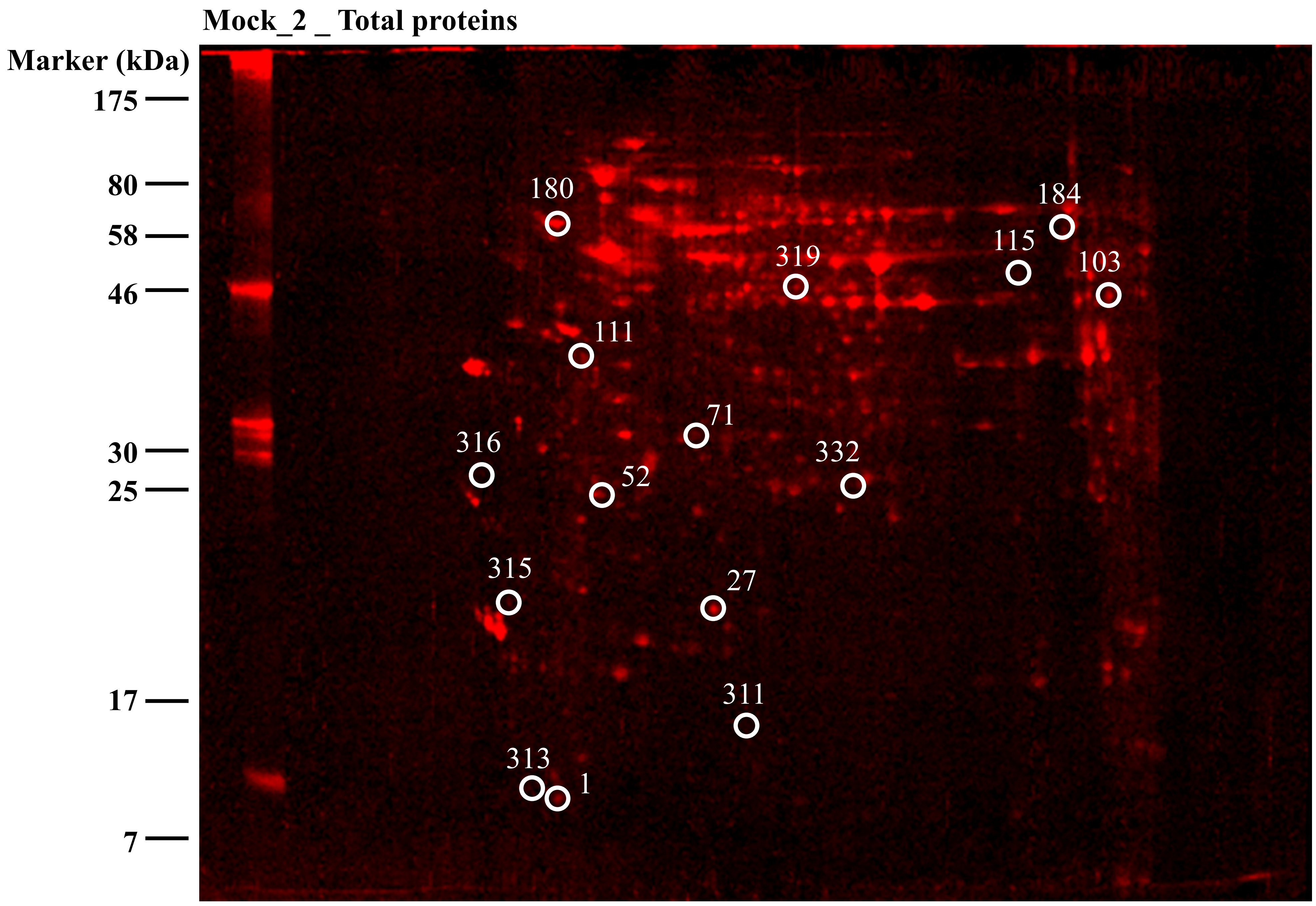
Mock_2 _ Total proteins
Marker (kDa)
175
80
58
46
30
25
17
7
180
184
115
319
103
111
71
316
332
52
315
27
311
313
1

Mock_3 _ Total proteins

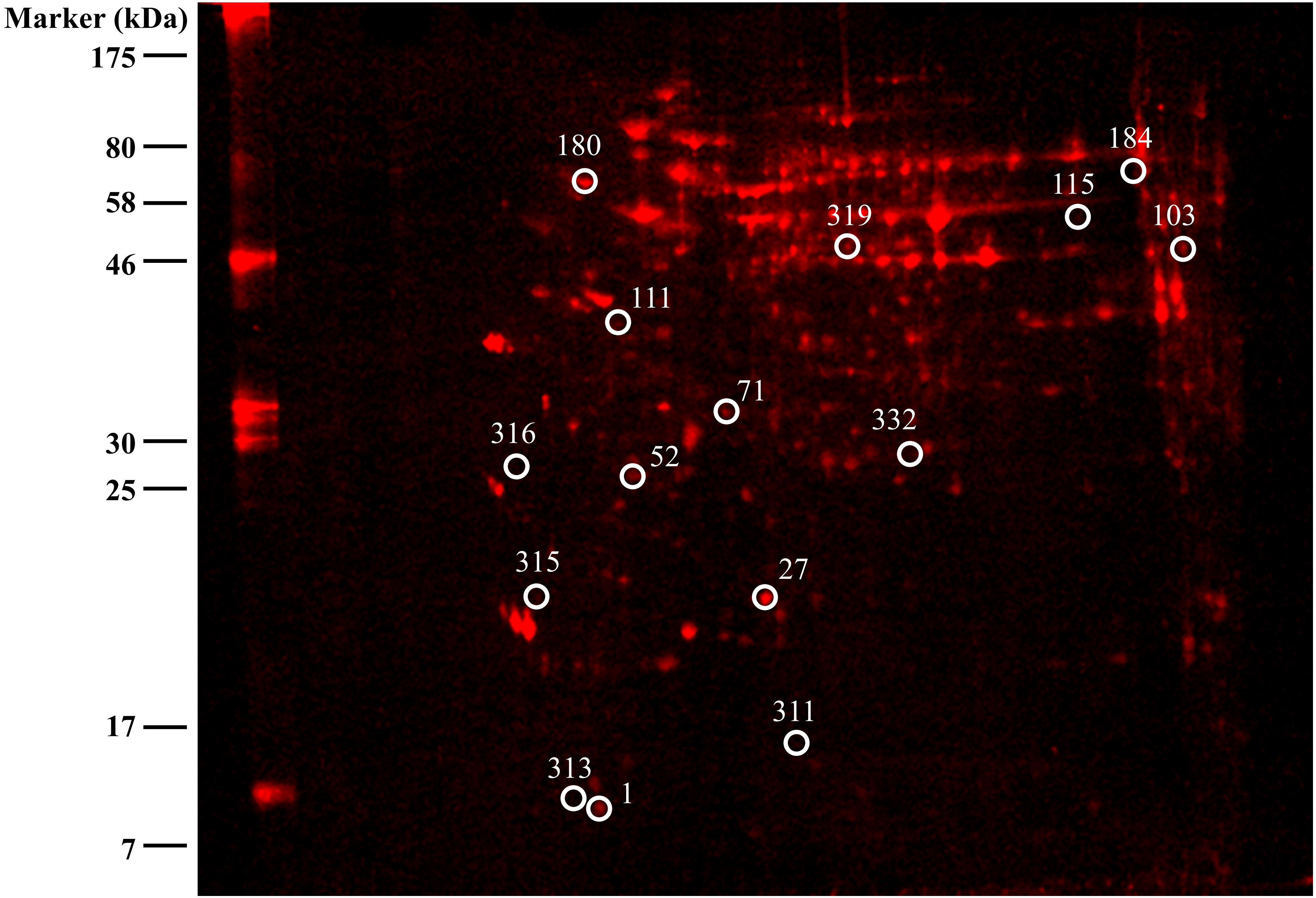

DENV 2_1 _ phosphoproteins

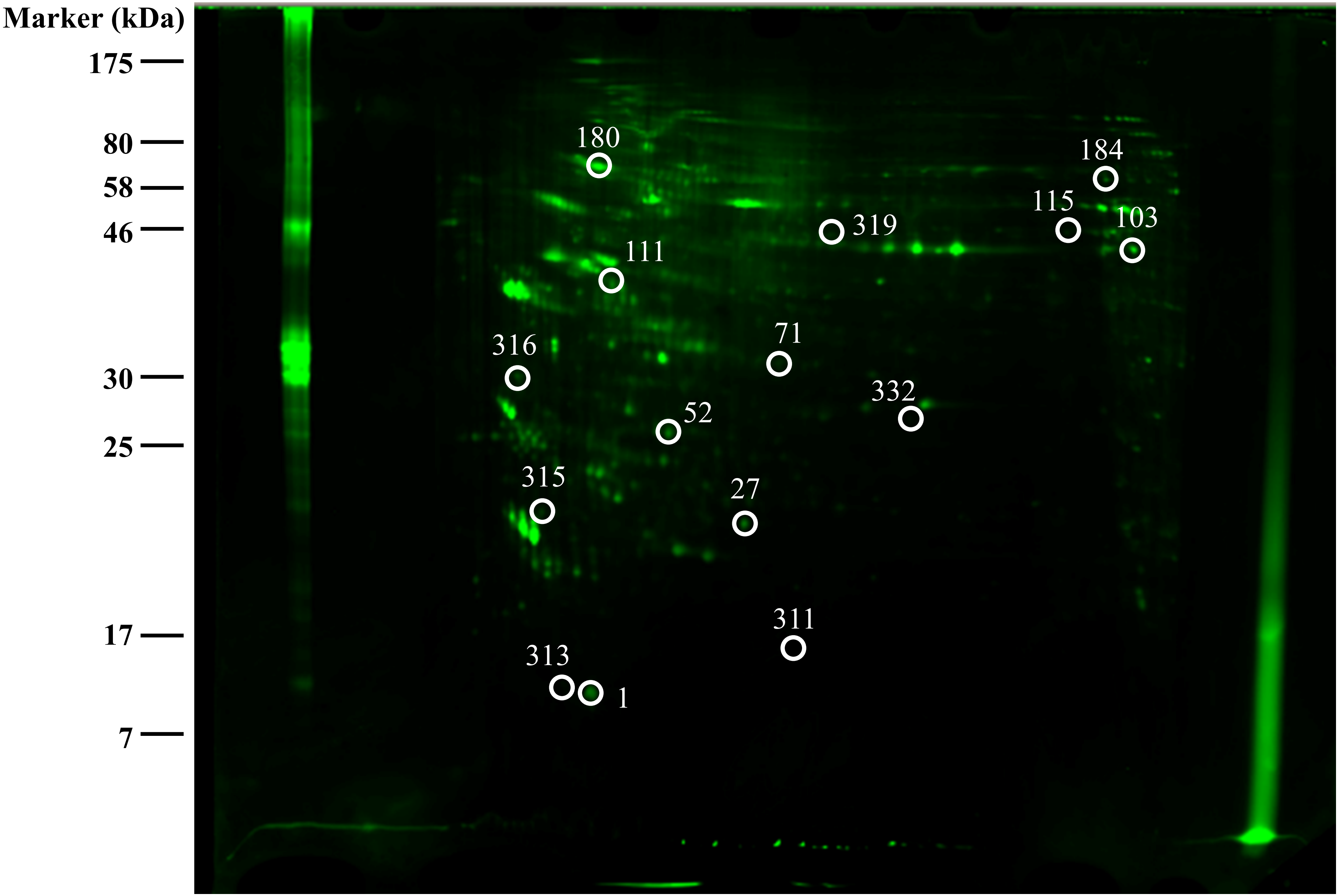

DENV 2_2 _ phosphoproteins

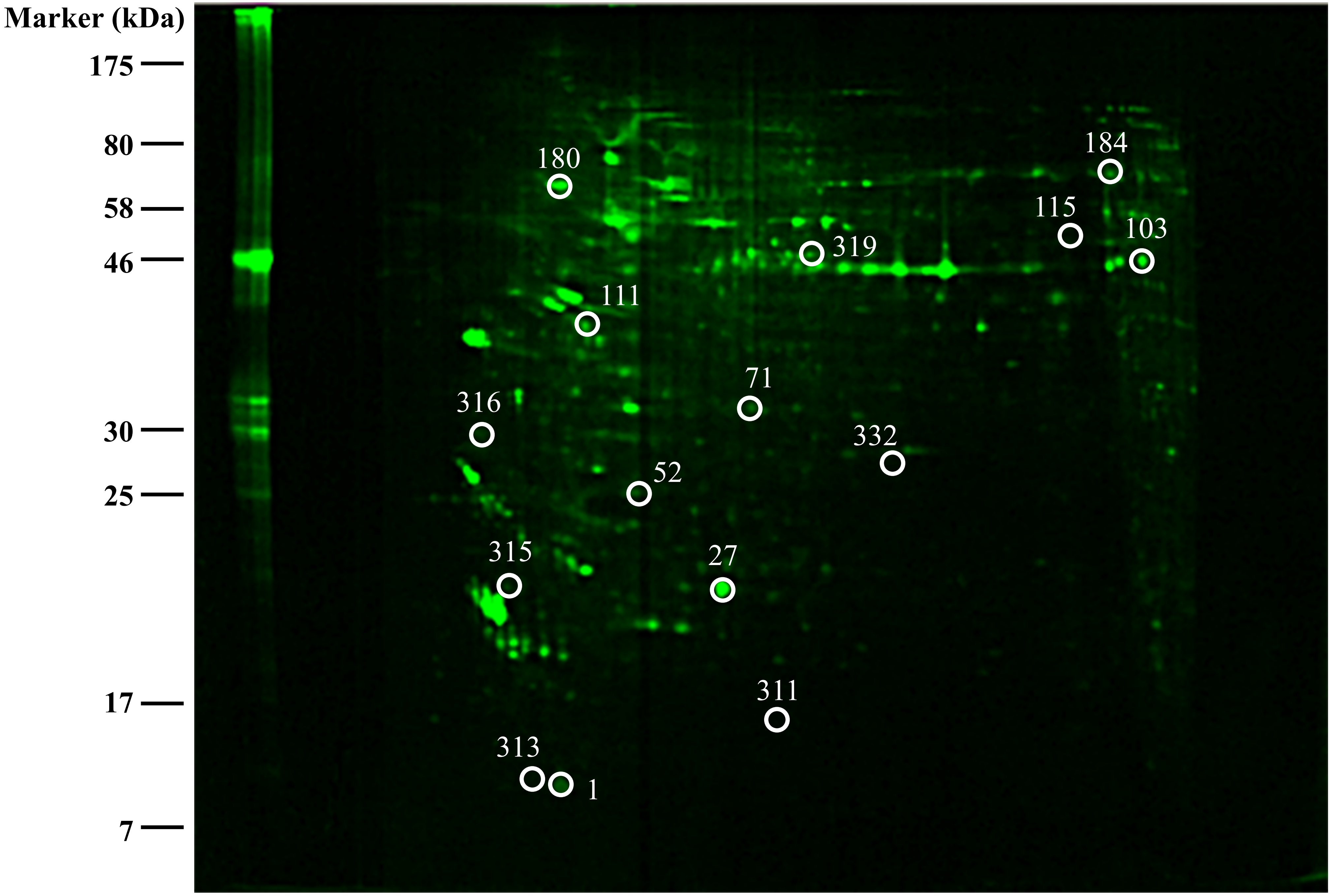

DENV 2_3 _ phosphoproteins

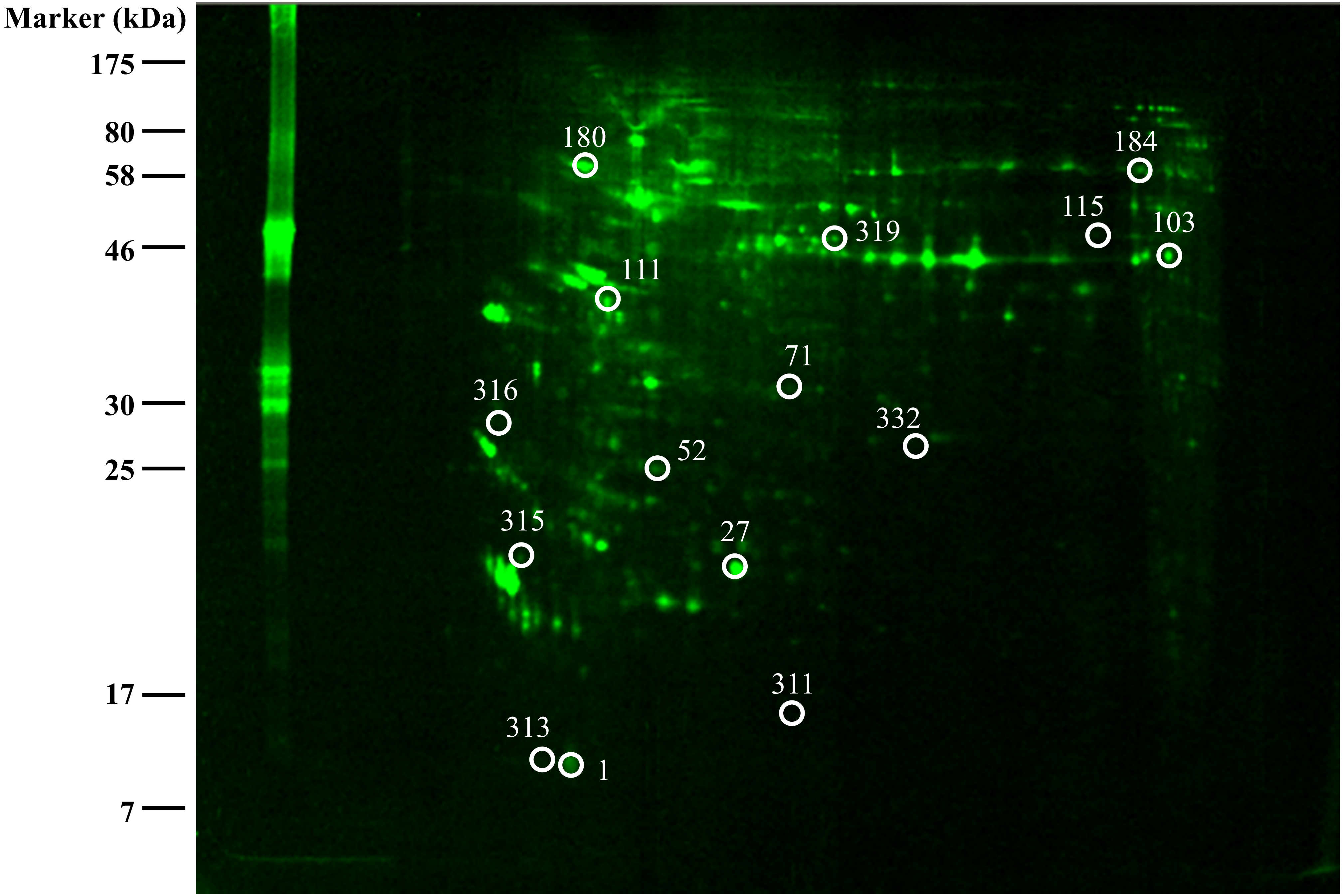

DENV 2_1 _ Total proteins

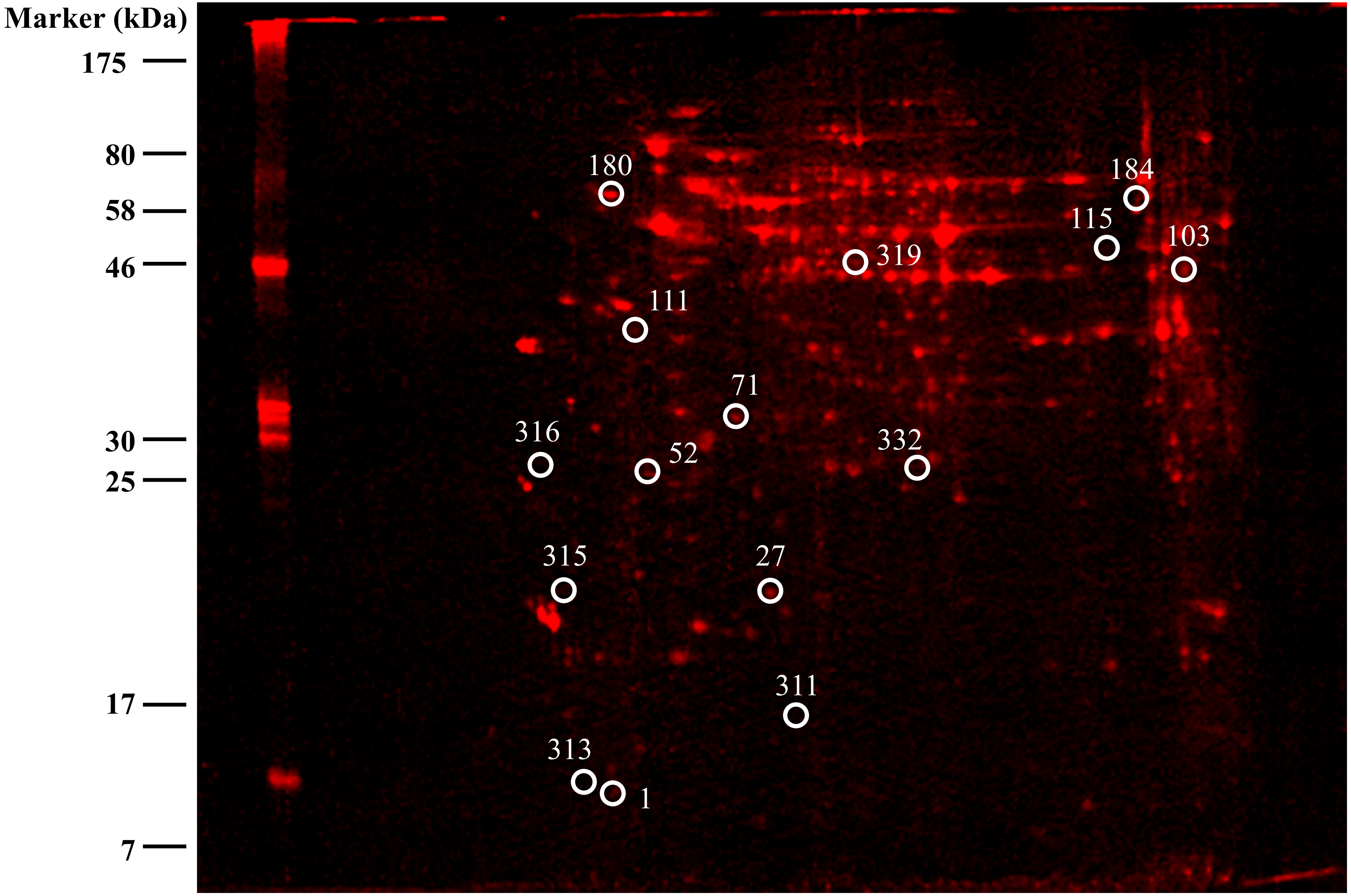

DENV 2_2 _ Total proteins

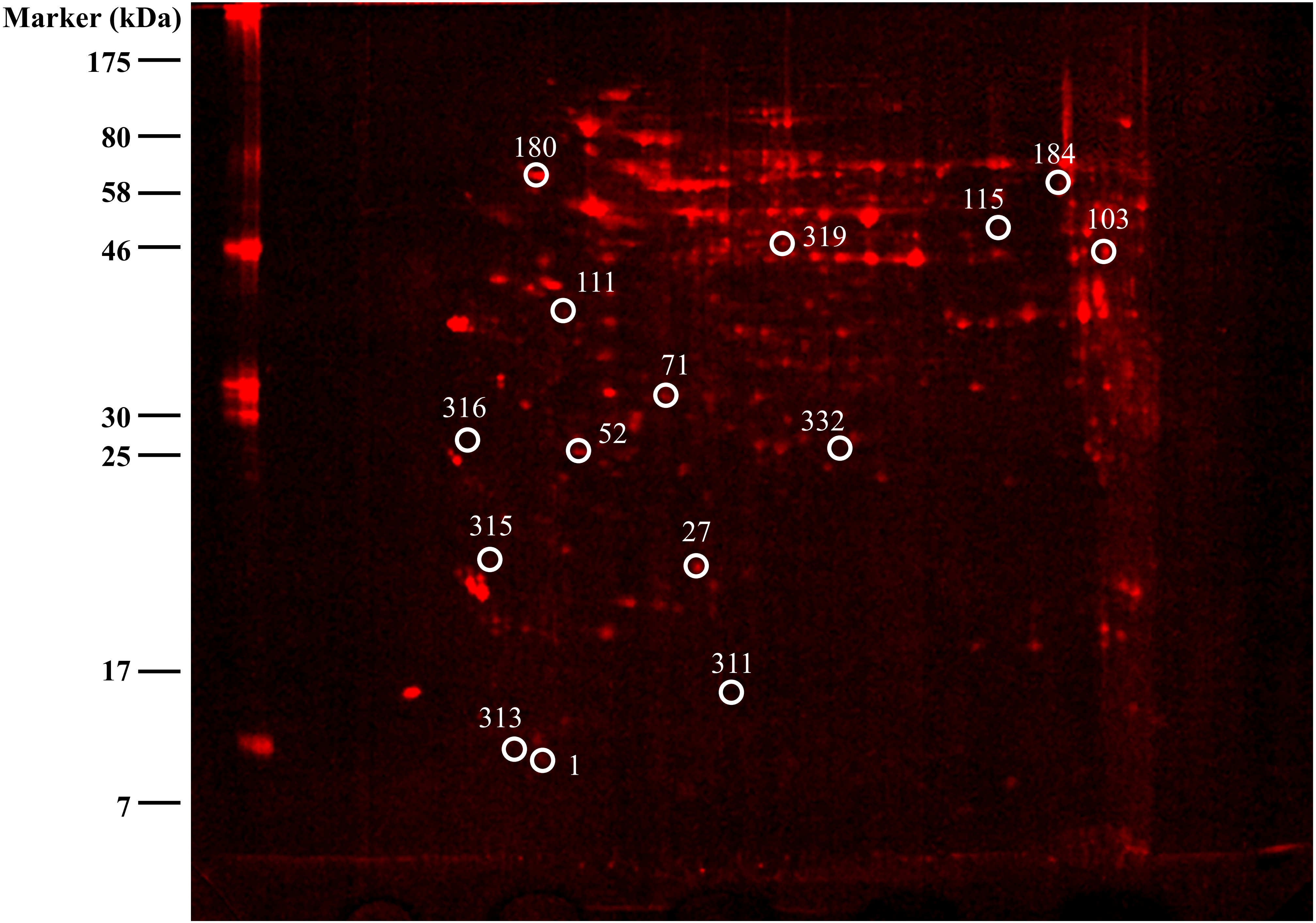

DENV 2_3 _ Total proteins

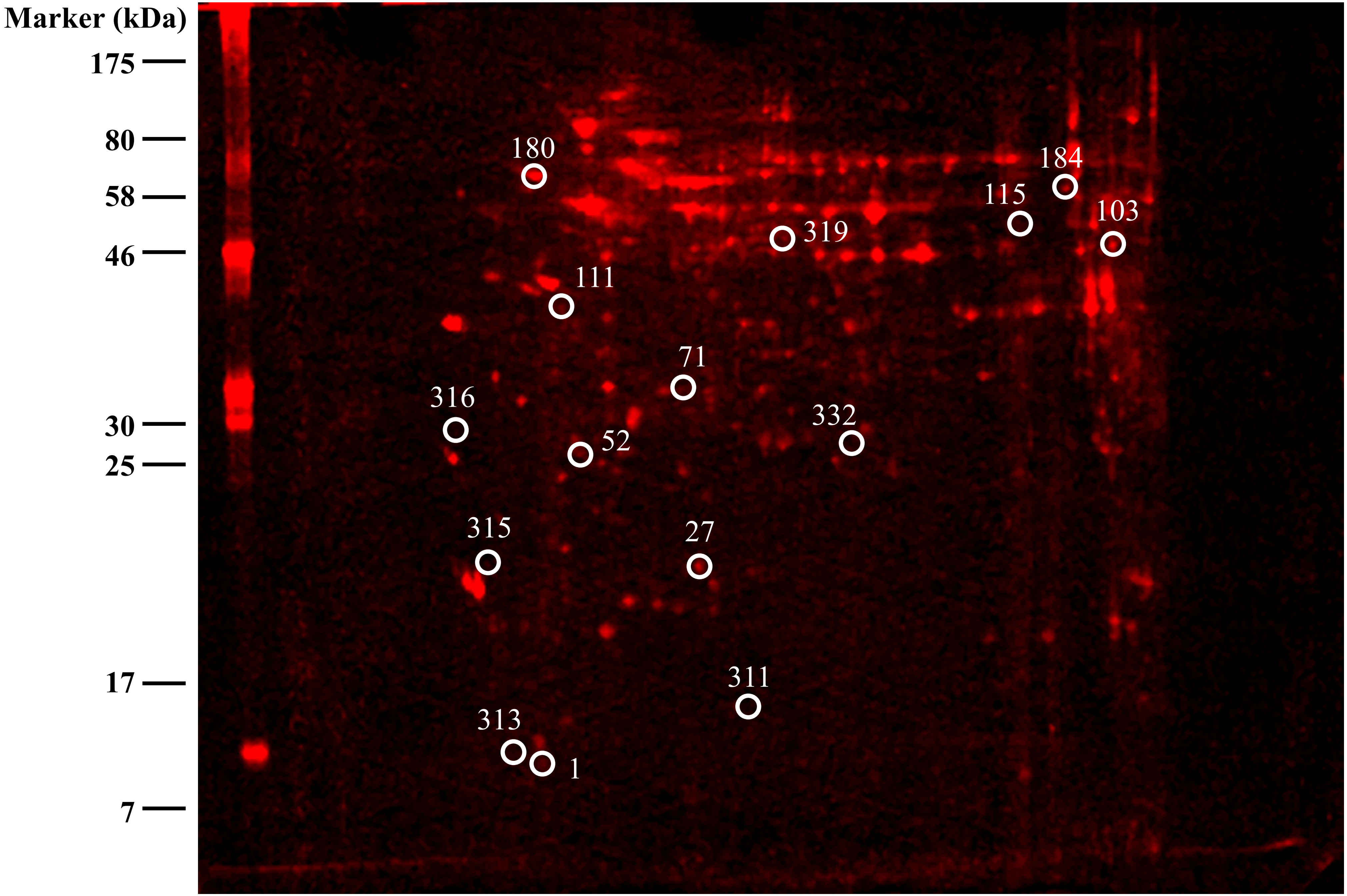

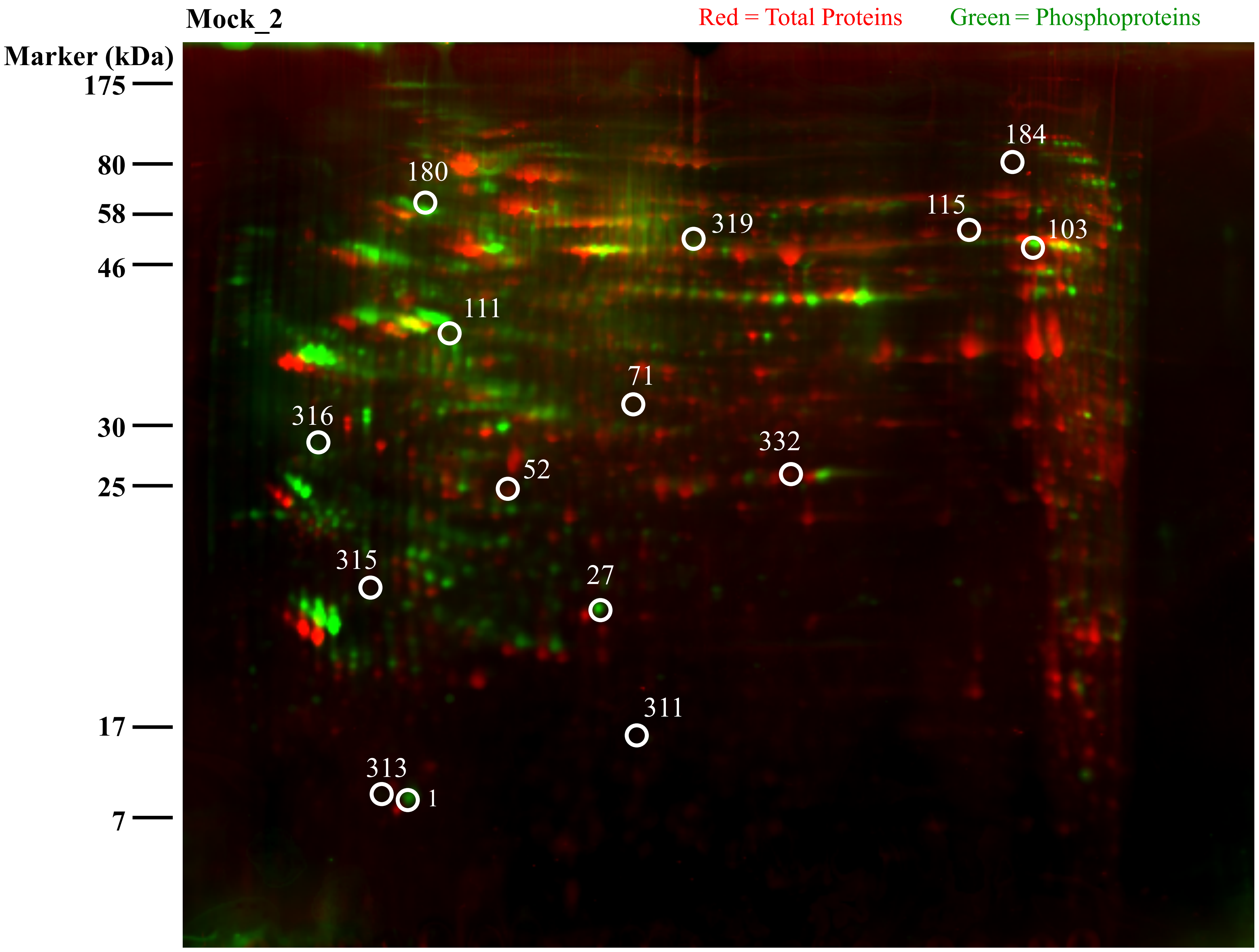

Mock_2
Red = Total Proteins
Green = Phosphoproteins
Marker (kDa)
175
80
58
46
30
25
17
7
180
184
115
103
319
111
71
316
332
52
315
27
311
313
1

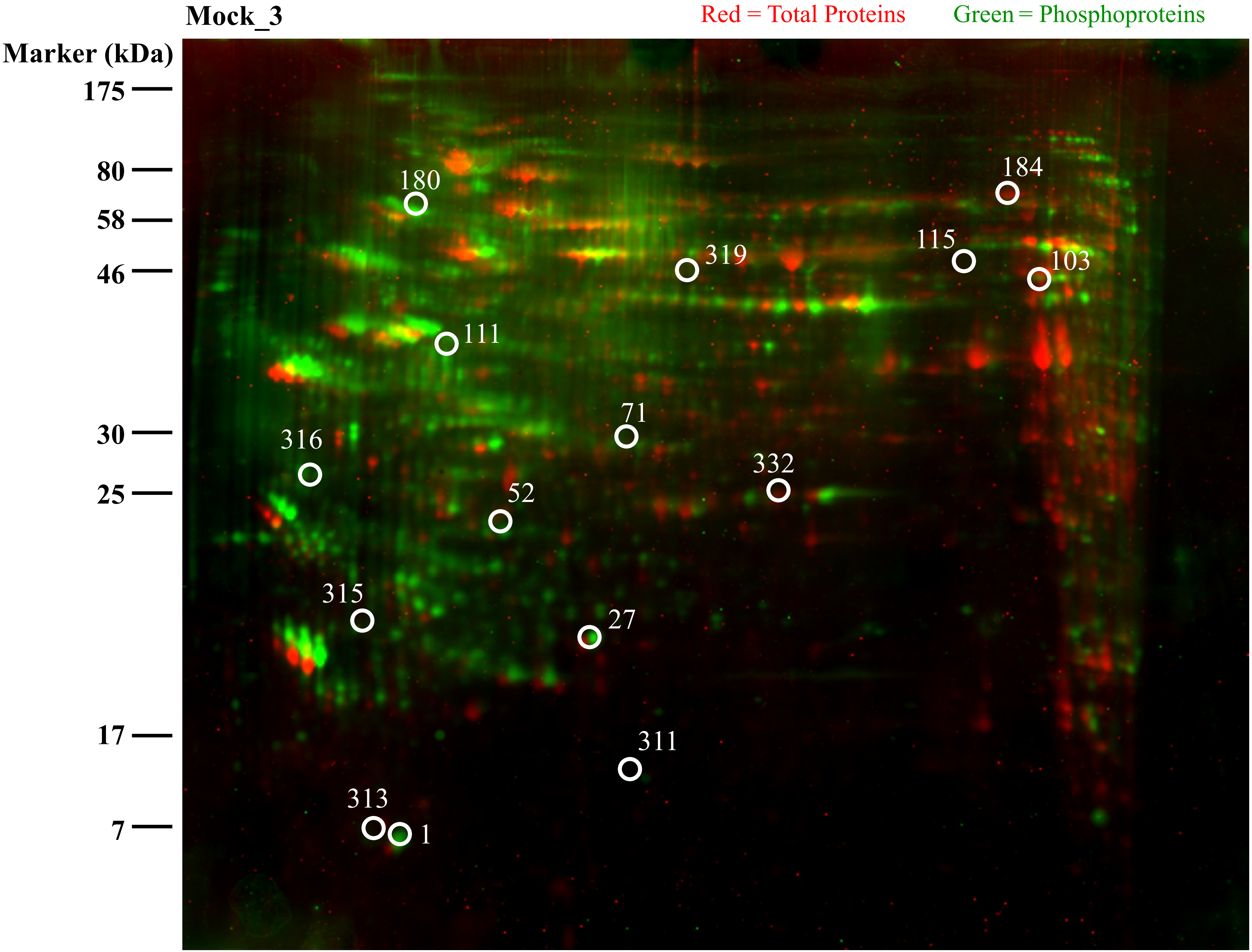

Mock_3
Red = Total Proteins
Green = Phosphoproteins
Marker (kDa)
175
80
58
46
30
25
17
7
180
184
115
319
103
111
71
316
332
52
315
27
311
313
1

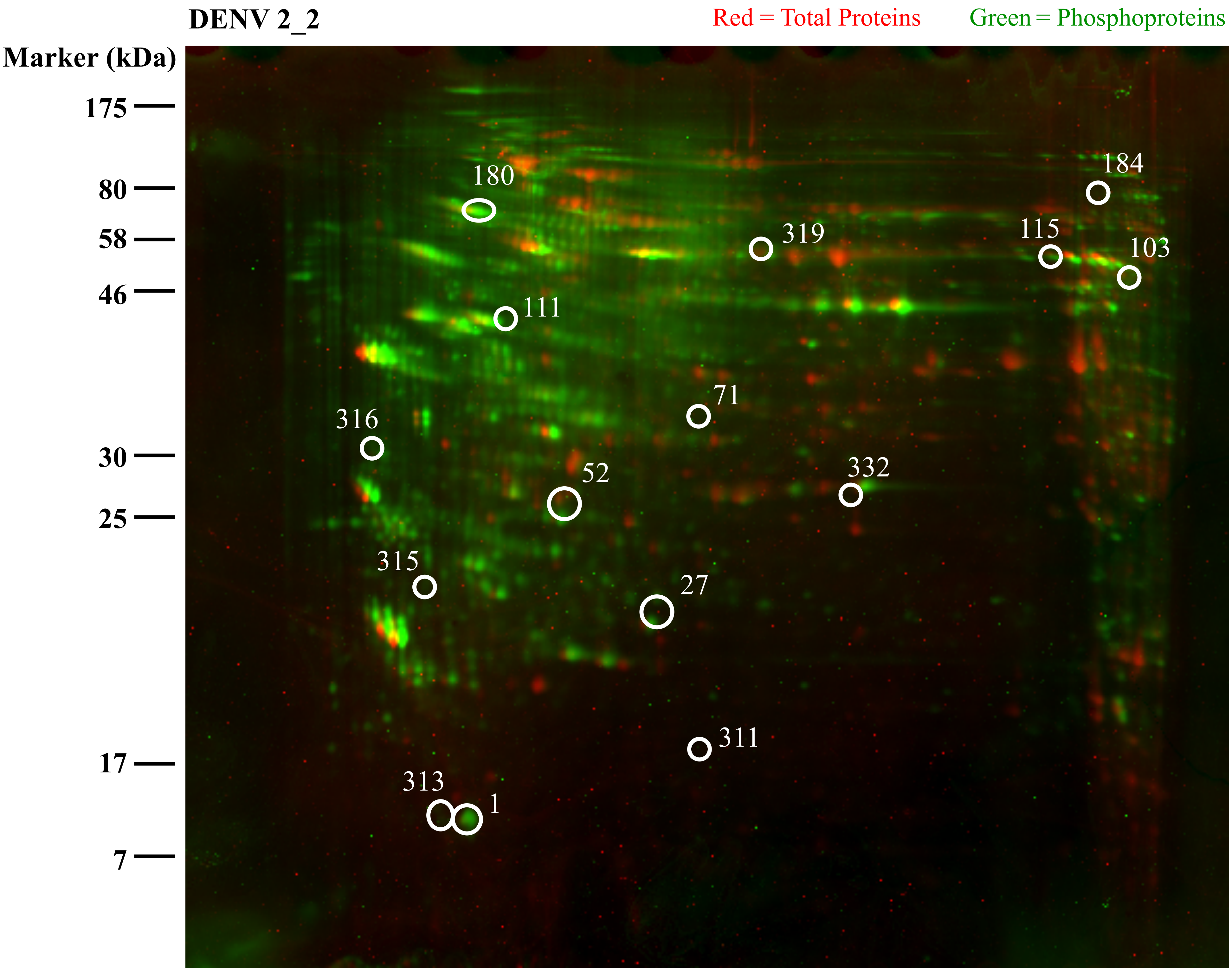
DENV 2_2
Red = Total Proteins
Green = Phosphoproteins
Marker (kDa)
175
80
58
46
30
25
17
7
180
184
319
115
103
111
71
316
332
52
315
27
311
313
1

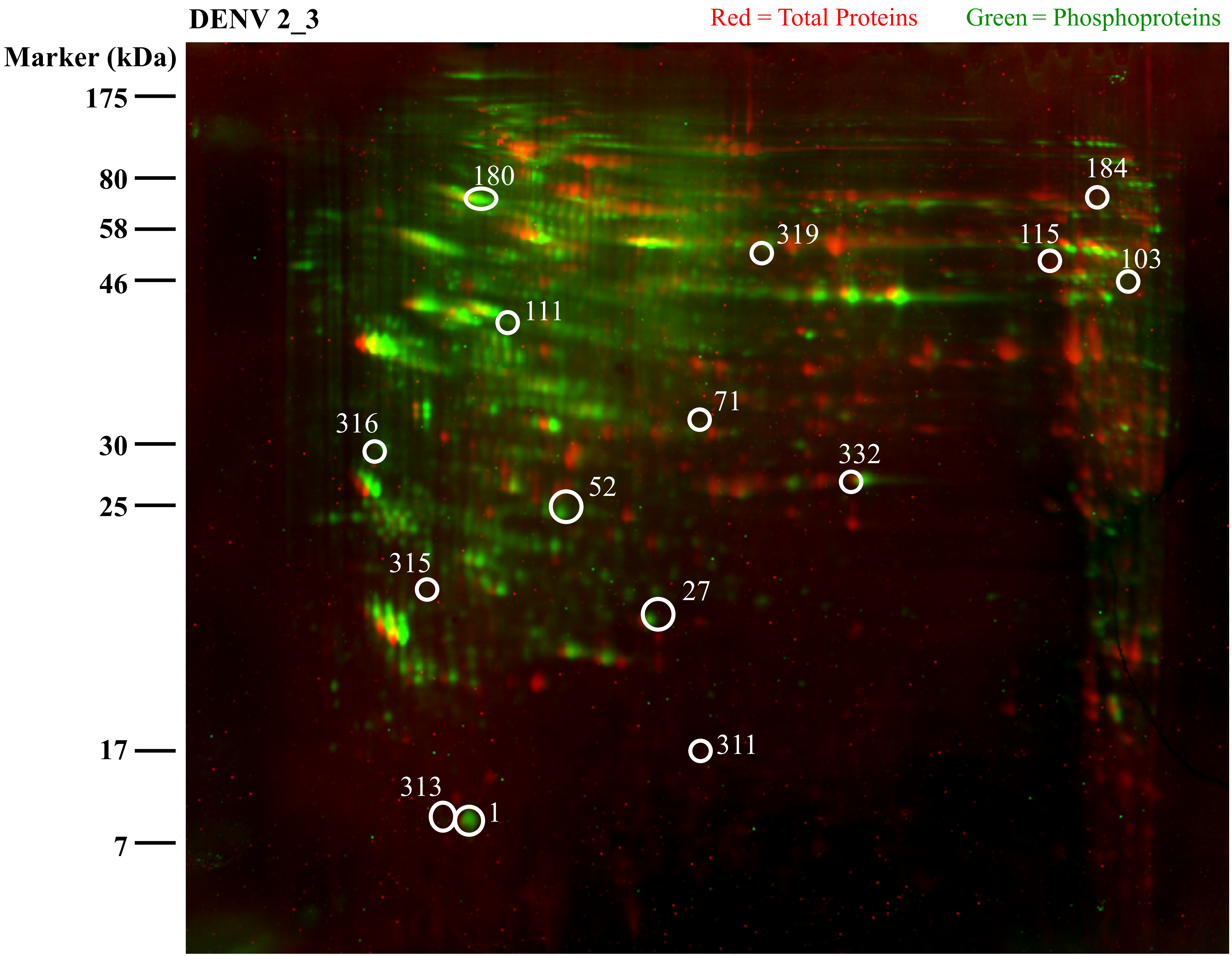
DENV 2_3
Red = Total Proteins
Green = Phosphoproteins
Marker (kDa)
175
80
58
46
30
25
17
7
180
184
319
115
103
111
71
316
332
52
315
27
311
313
1

Full uncropped western blot image for figure 3
p-PKM2 Tyr105: set 1 (used in manuscript figure 3A)

Immunoblot: p-PKM2 Tyr105

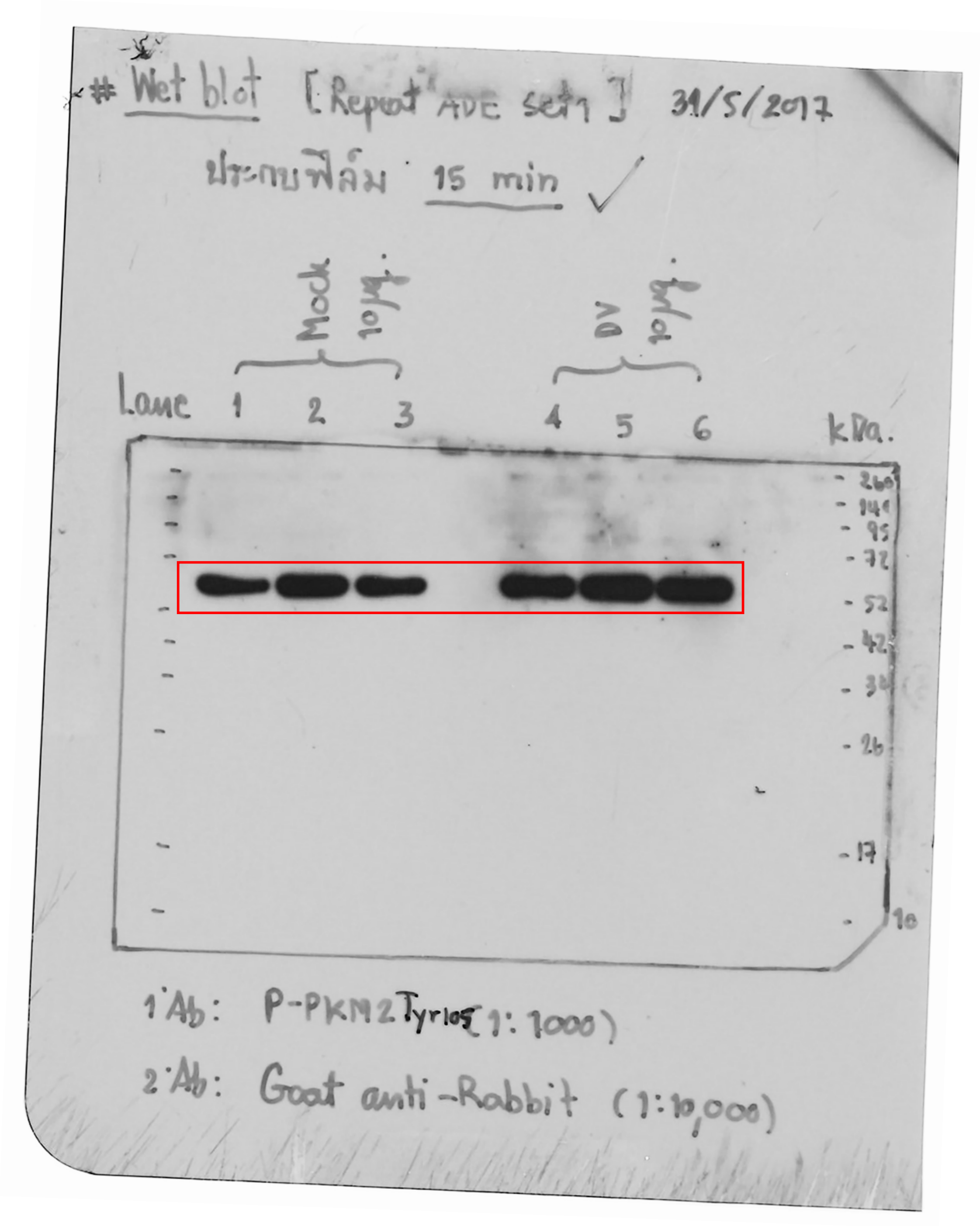

Red box represents the cropped image used in manuscript figure 3A.

Full uncropped western blot image for figure 3
Total PKM2: set 1 (used in manuscript figure 3A)

Immunoblot: total PKM2

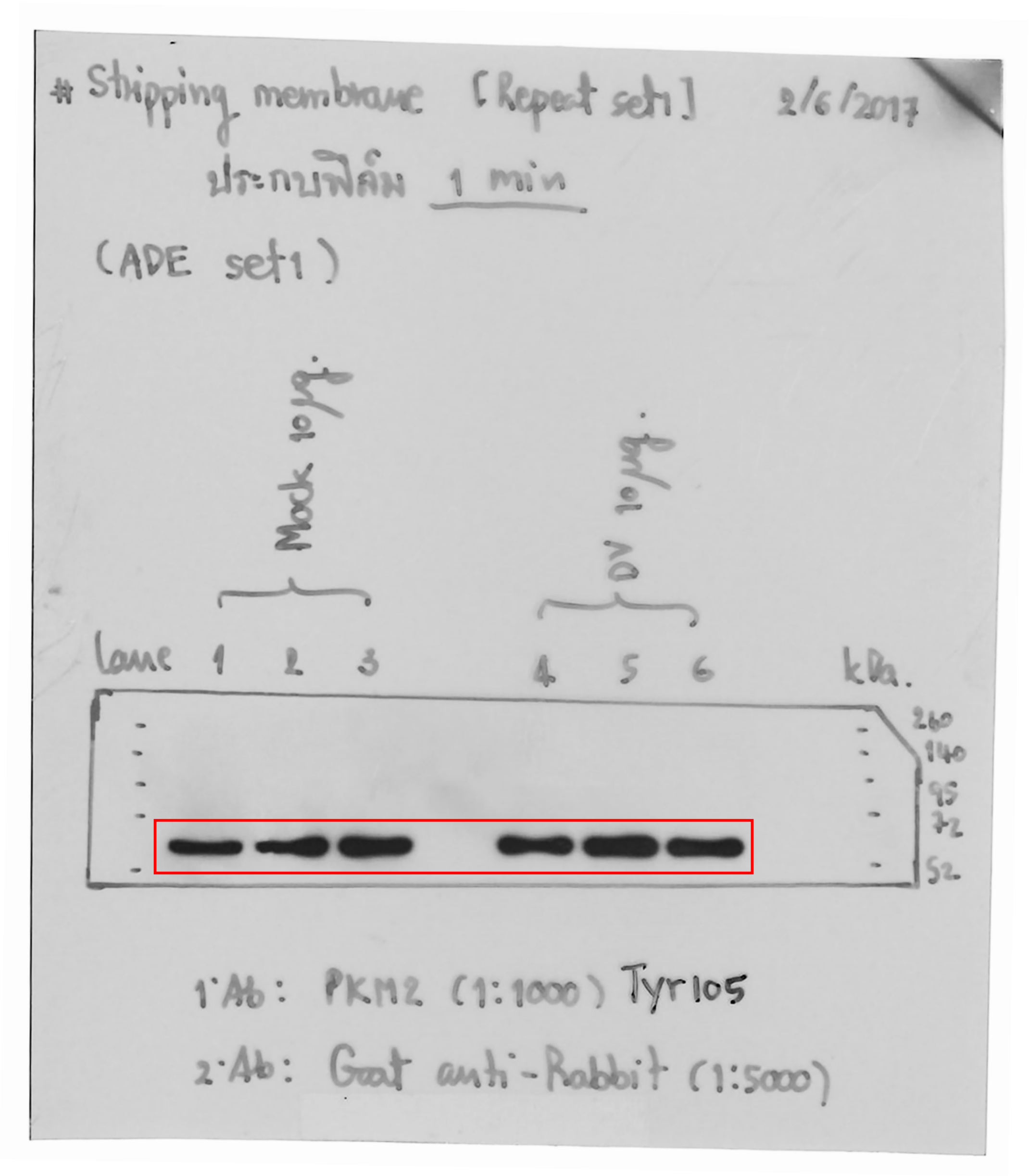

Red box represents the cropped image used in manuscript figure 3A.

Full uncropped western blot image for figure 3
GAPDH protein: set 1 (used in manuscript figure 3A)

Immunoblot: GAPDH

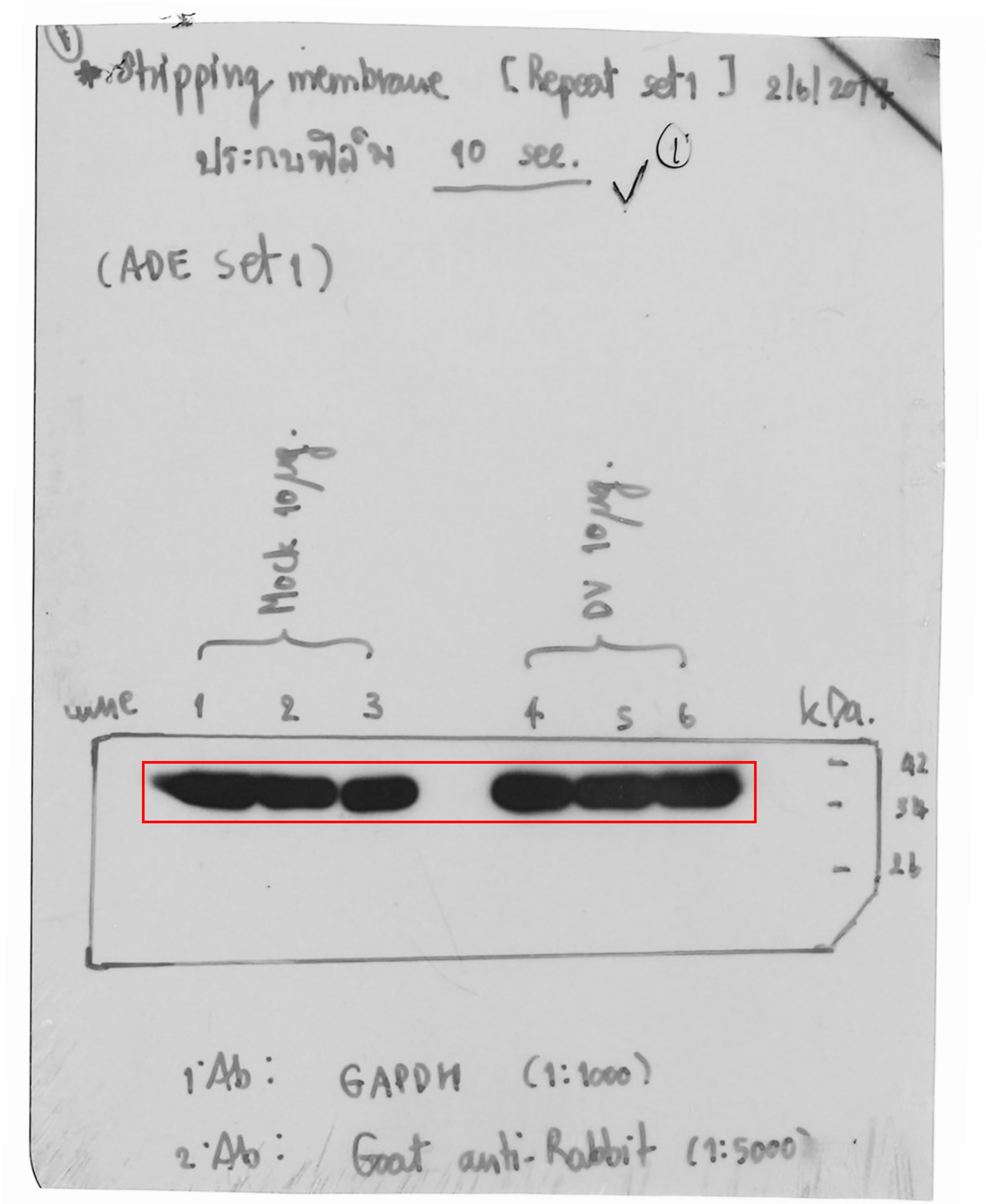

Red box represents the cropped image used in manuscript figure 3A.

Full uncropped western blot image for figure 3
p-PKM2 Tyr105: set 2

Immunoblot: p-PKM2 Tyr105

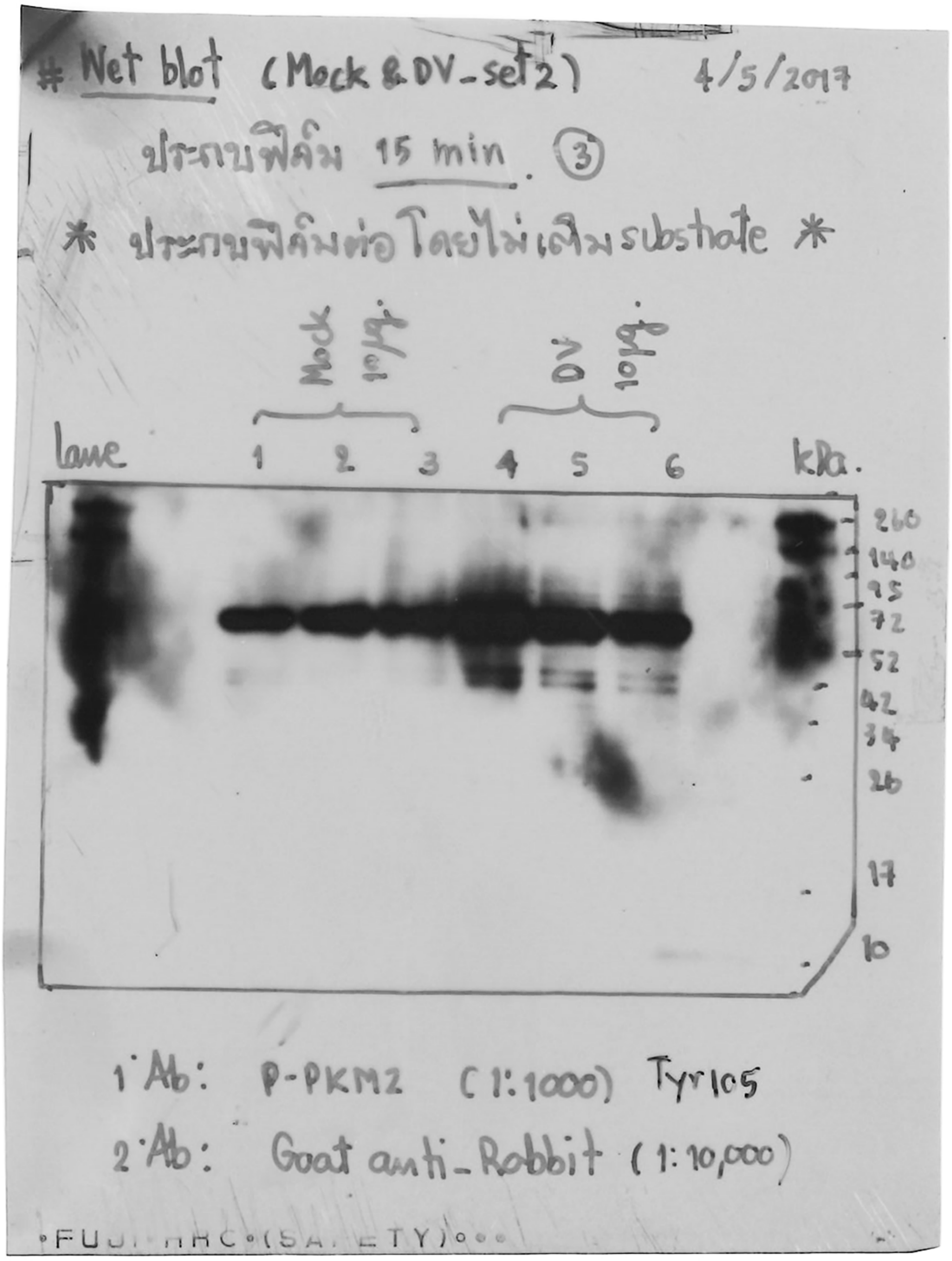

Full uncropped western blot image for figure 3
Total PKM2: set 2

Immunoblot: total PKM2

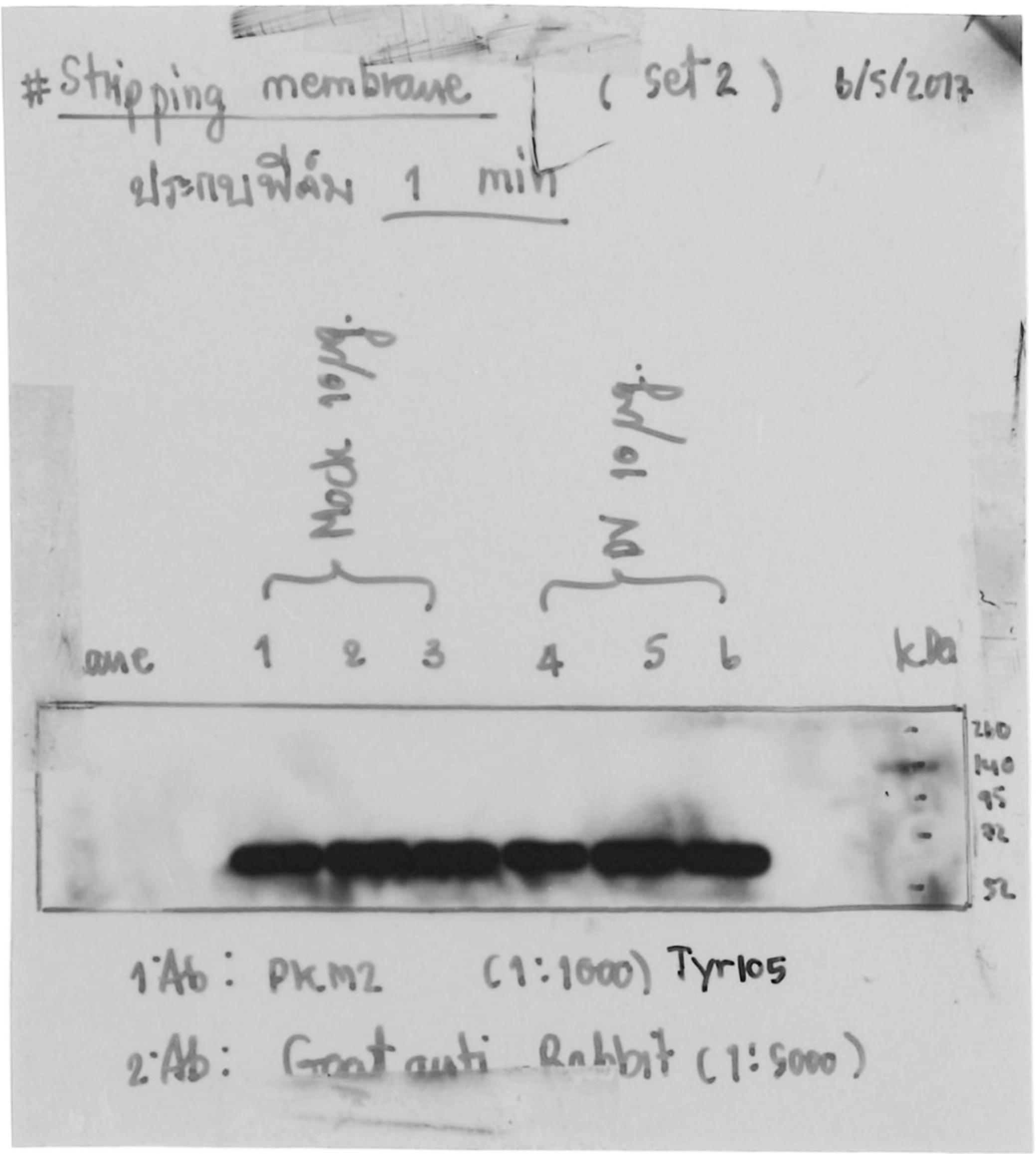

Full uncropped western blot image for figure 3
GAPDH protein: set 2

Immunoblot: GAPDH

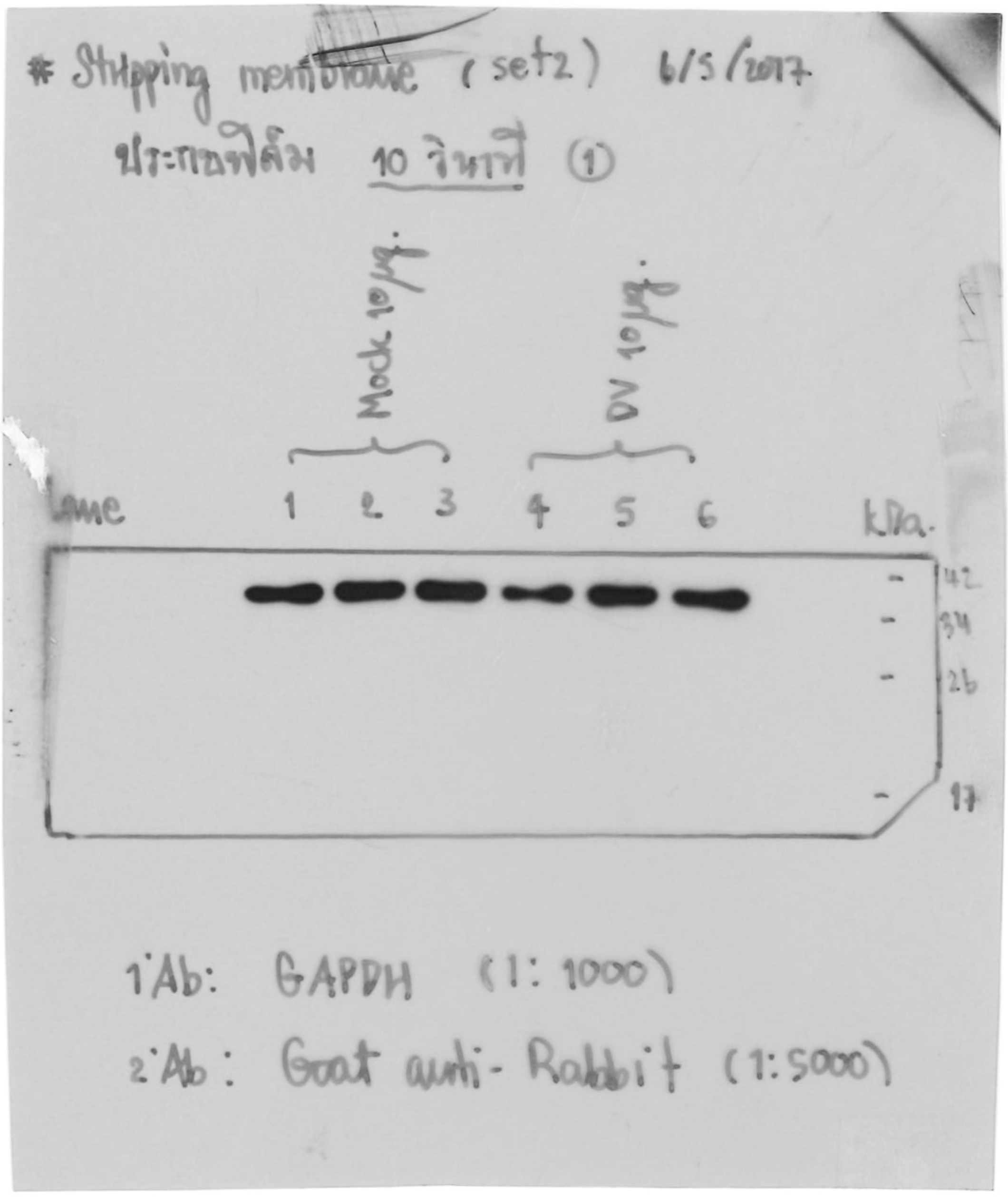

Full uncropped western blot image for figure 3
p-PKM2 Tyr105: set 3

Immunoblot: p-PKM2 Tyr105

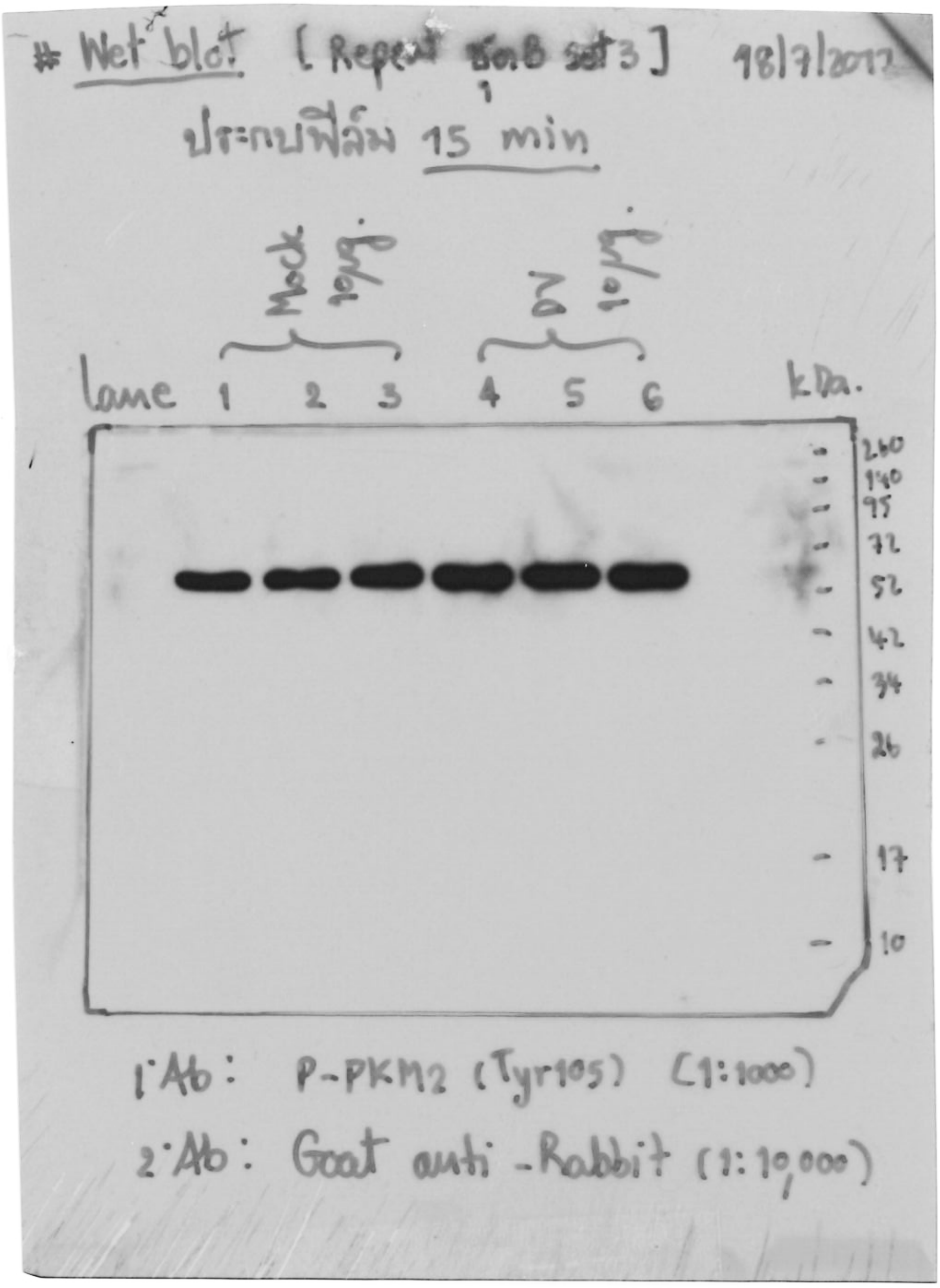

Full uncropped western blot image for figure 3
Total PKM2: set 3

Immunoblot: total PKM2

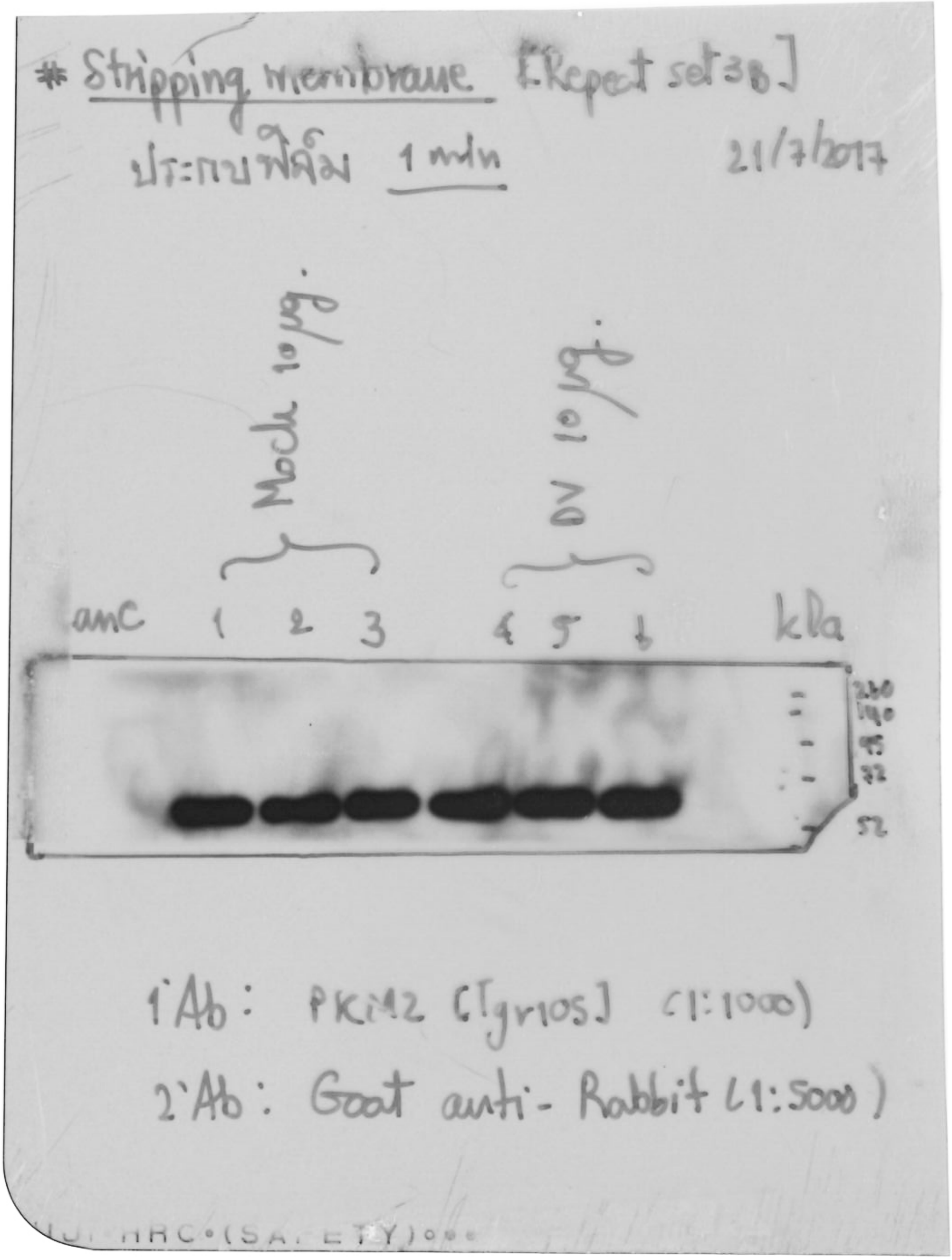

Full uncropped western blot image for figure 3
GAPDH protein: set 3

Immunoblot: GAPDH

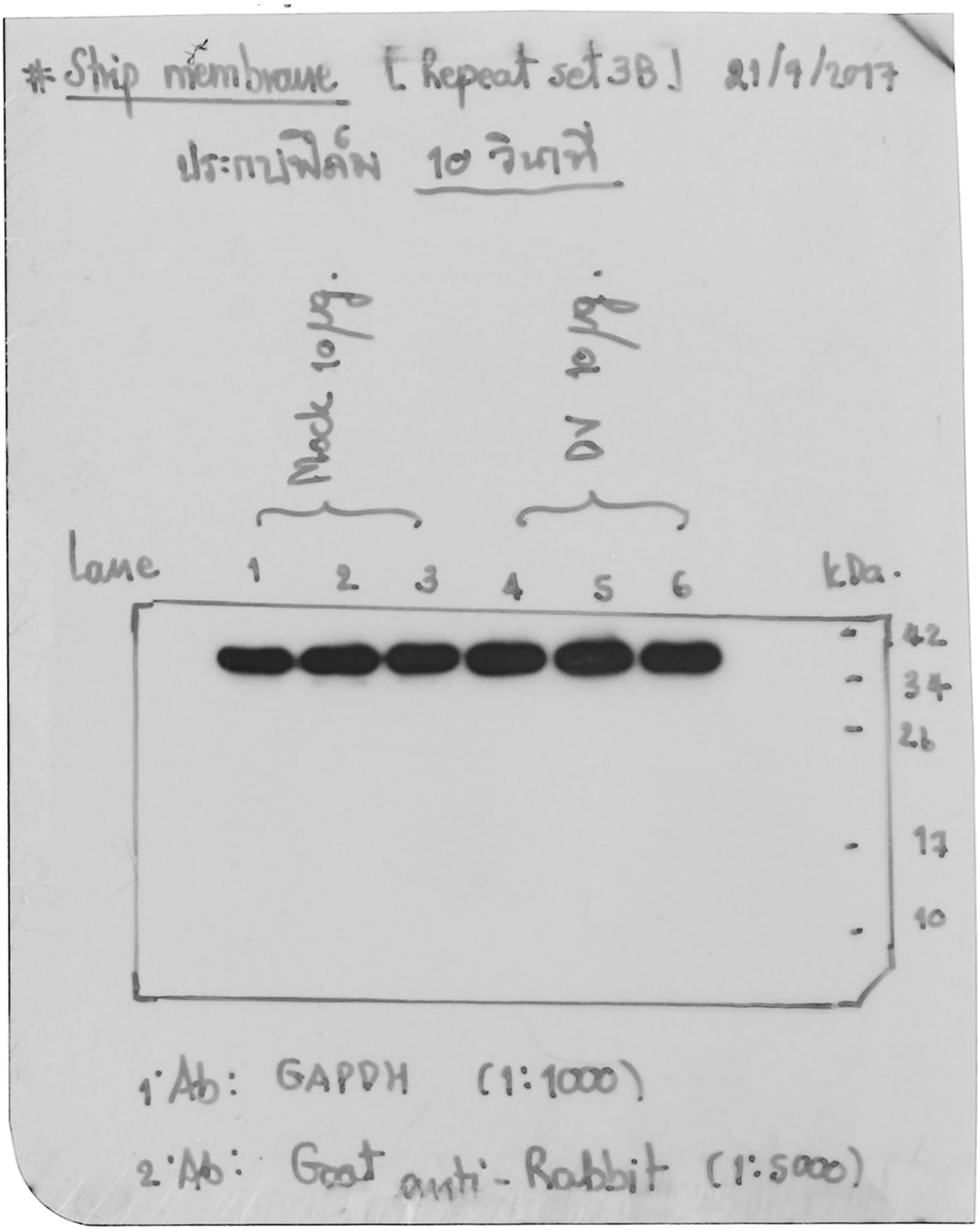

Full uncropped western blot image for figure 3
p-PKM2 Ser37: set 1 (used in manuscript figure 3B)

Immunoblot: p-PKM2 Ser37

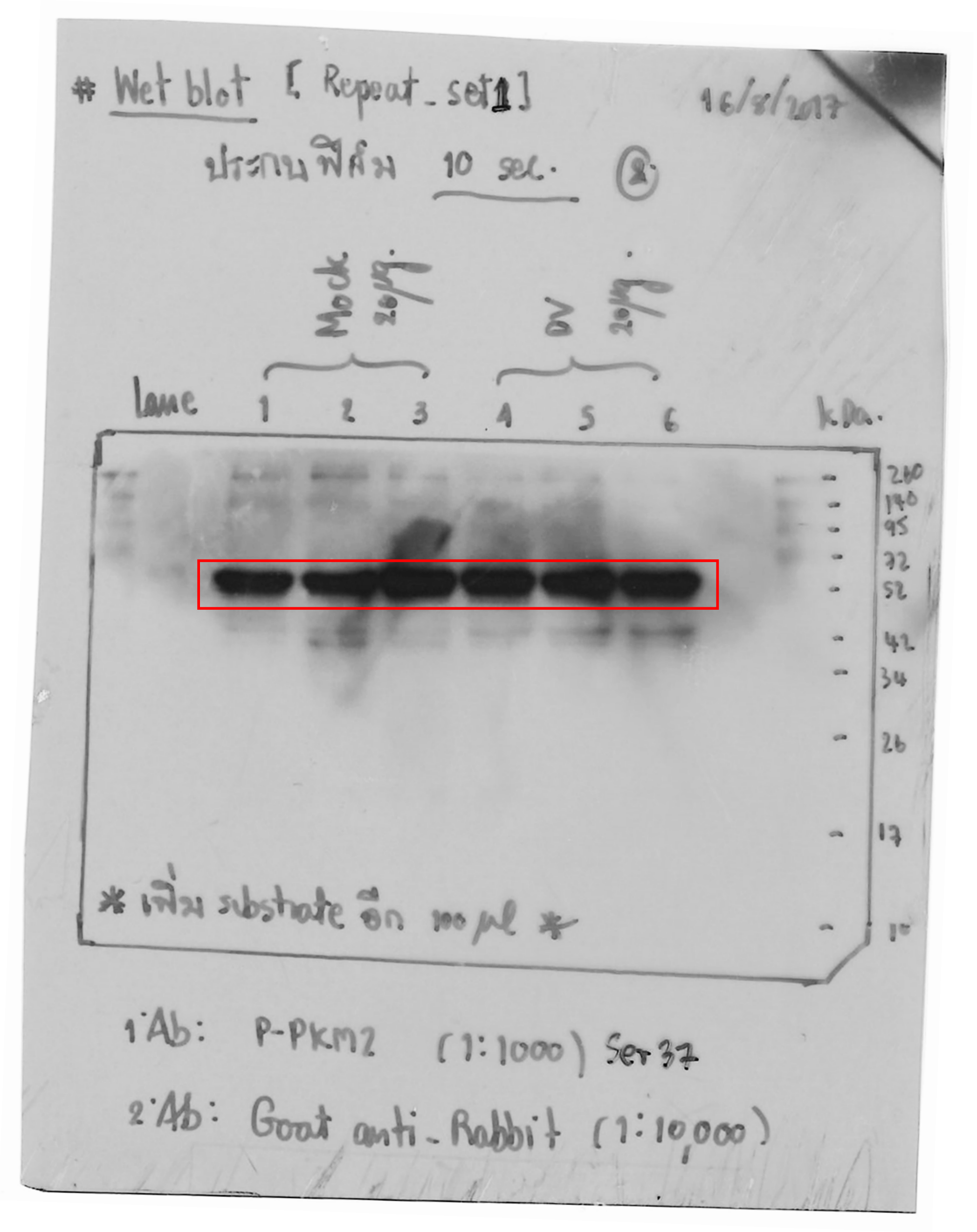

Red box represents the cropped image used in manuscript figure 3B.

Full uncropped western blot image for figure 3
Total PKM2: set 1 (used in manuscript figure 3B)

Immunoblot: total PKM2

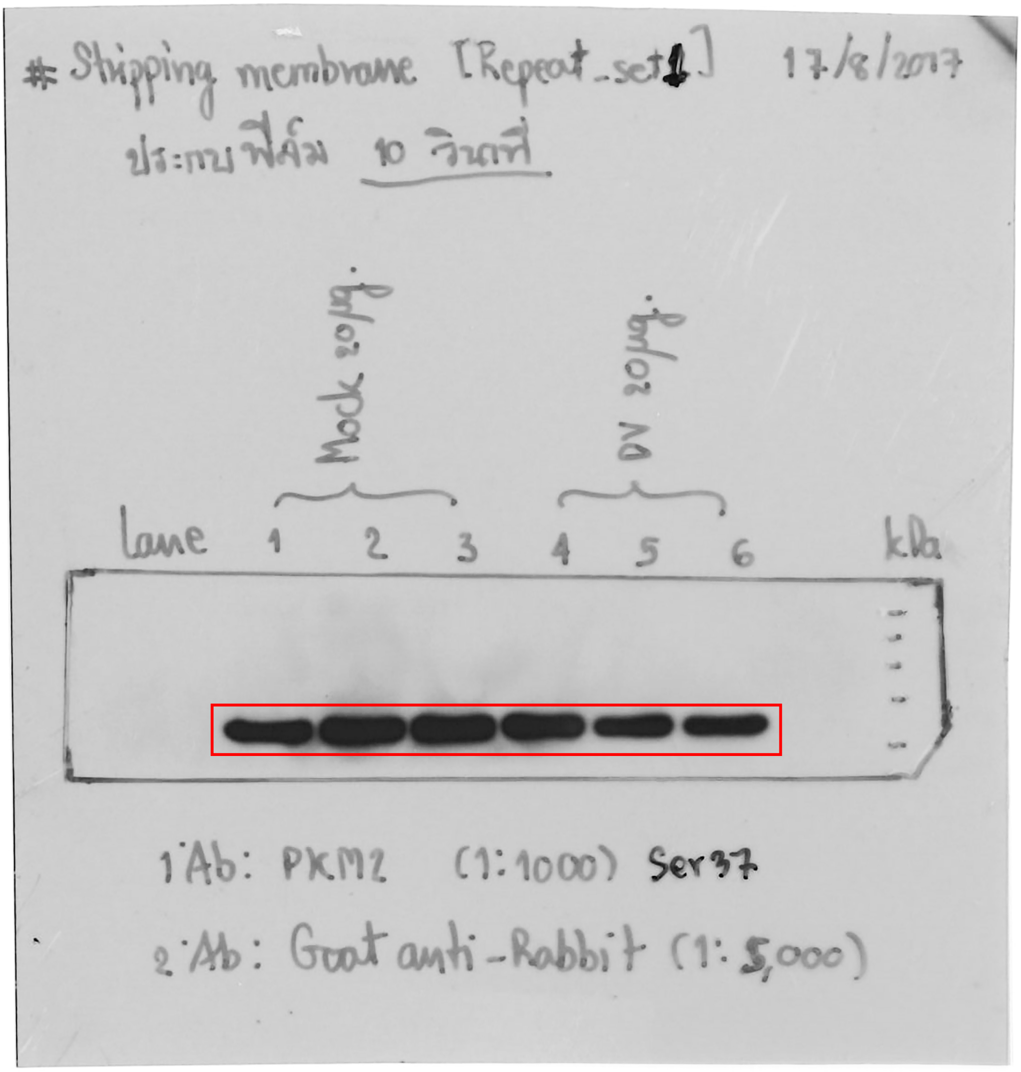

Red box represents the cropped image used in manuscript figure 3B.

Full uncropped western blot image for figure 3
GAPDH protein: set 1 (used in manuscript figure 3B)

Immunoblot: GAPDH

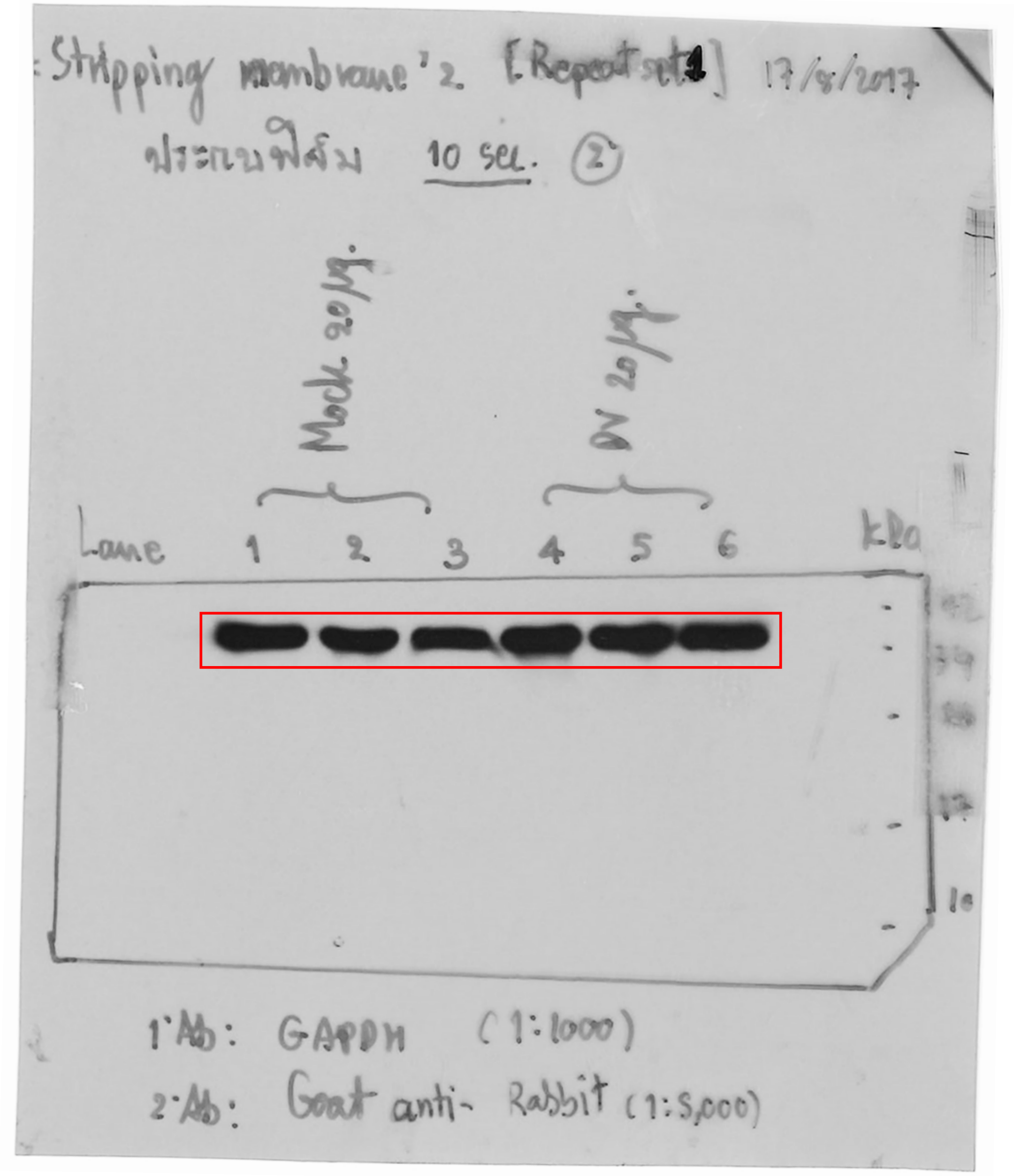

Red box represents the cropped image used in manuscript figure 3B.

Full uncropped western blot image for figure 3
p-PKM2 Ser37: set 2

Immunoblot: p-PKM2 Ser37

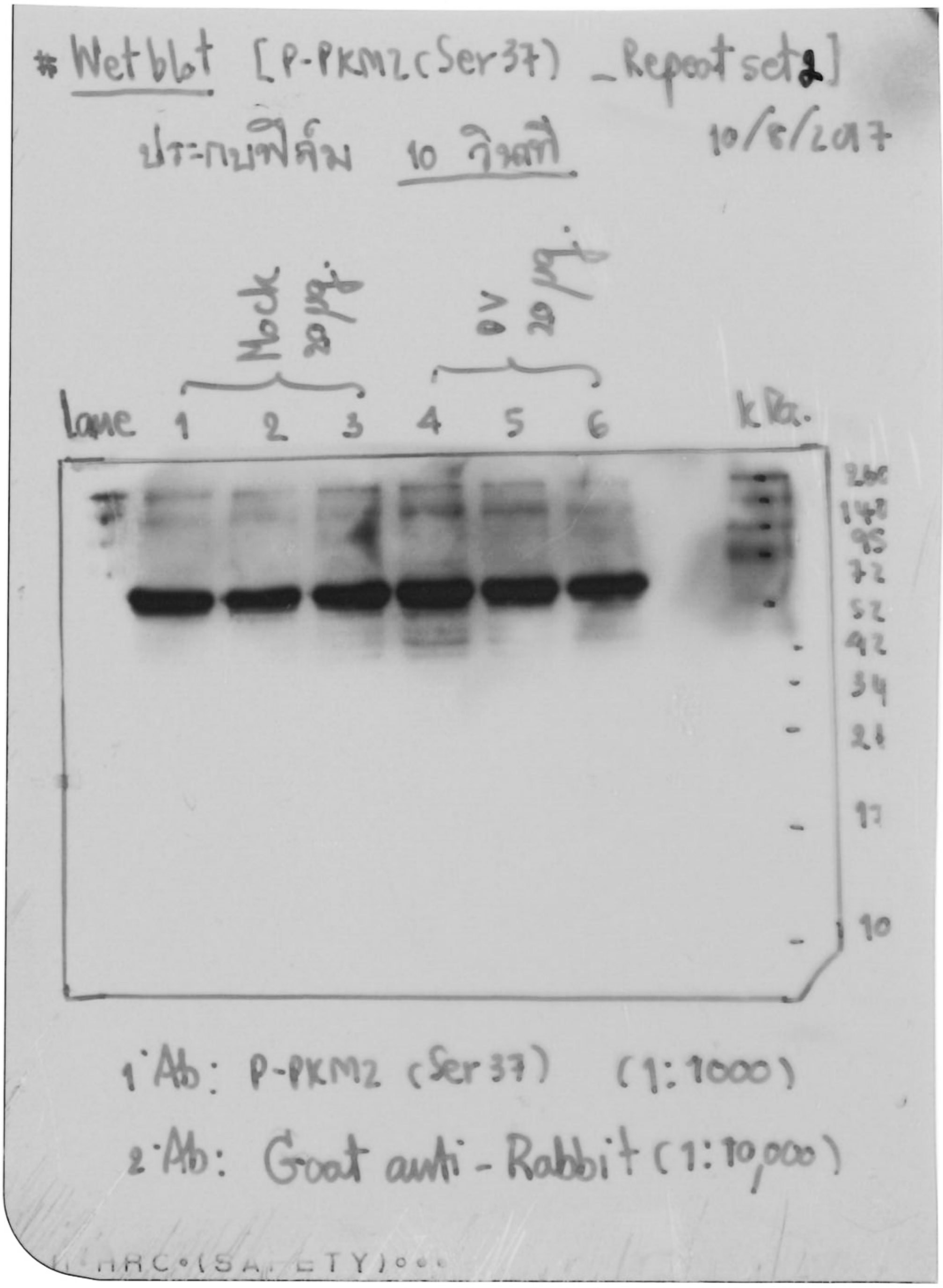

Full uncropped western blot image for figure 3
Total PKM2: set 2

Immunoblot: total PKM2

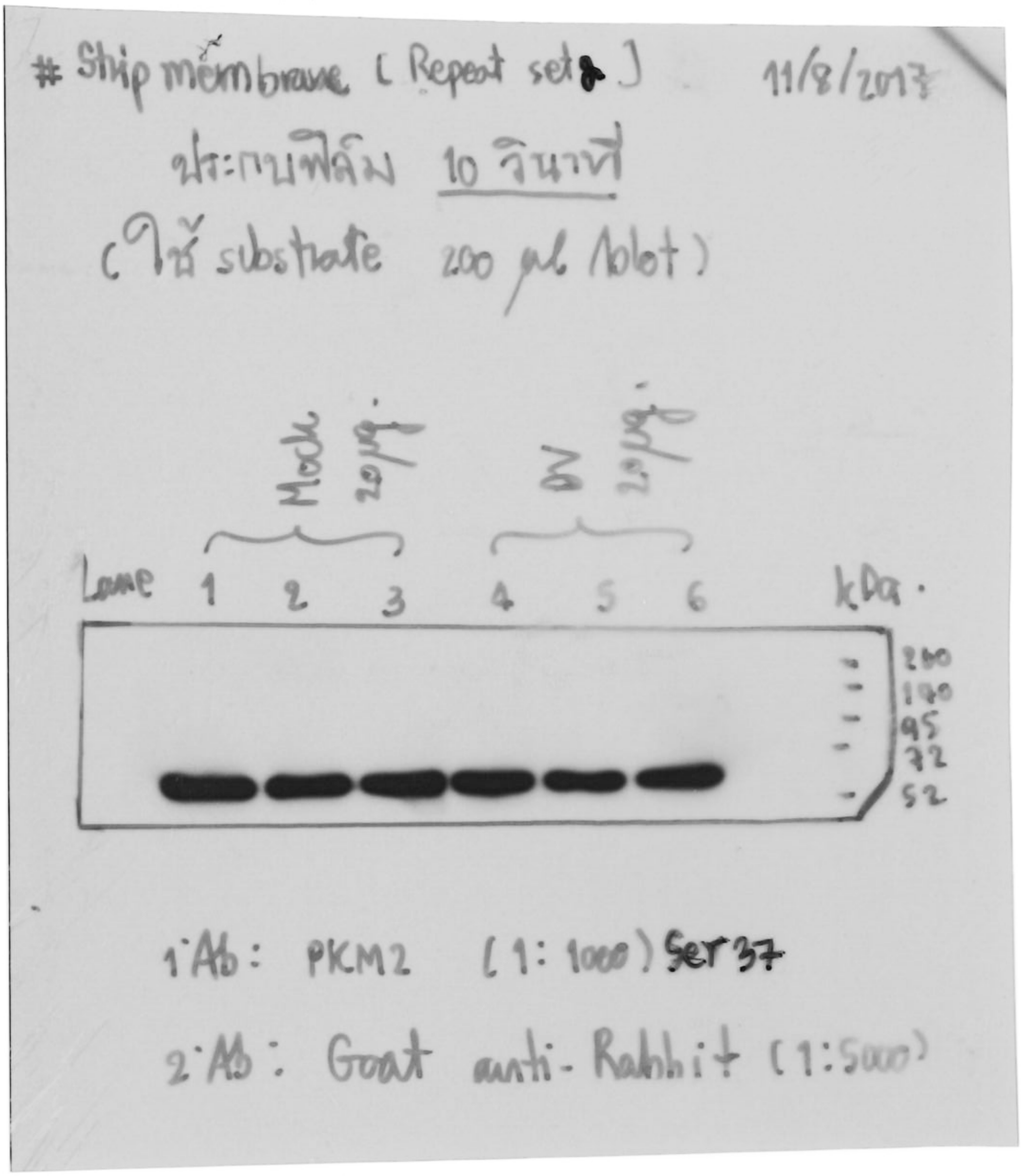

Full uncropped western blot image for figure 3
GAPDH protein: set 2

Immunoblot: GAPDH

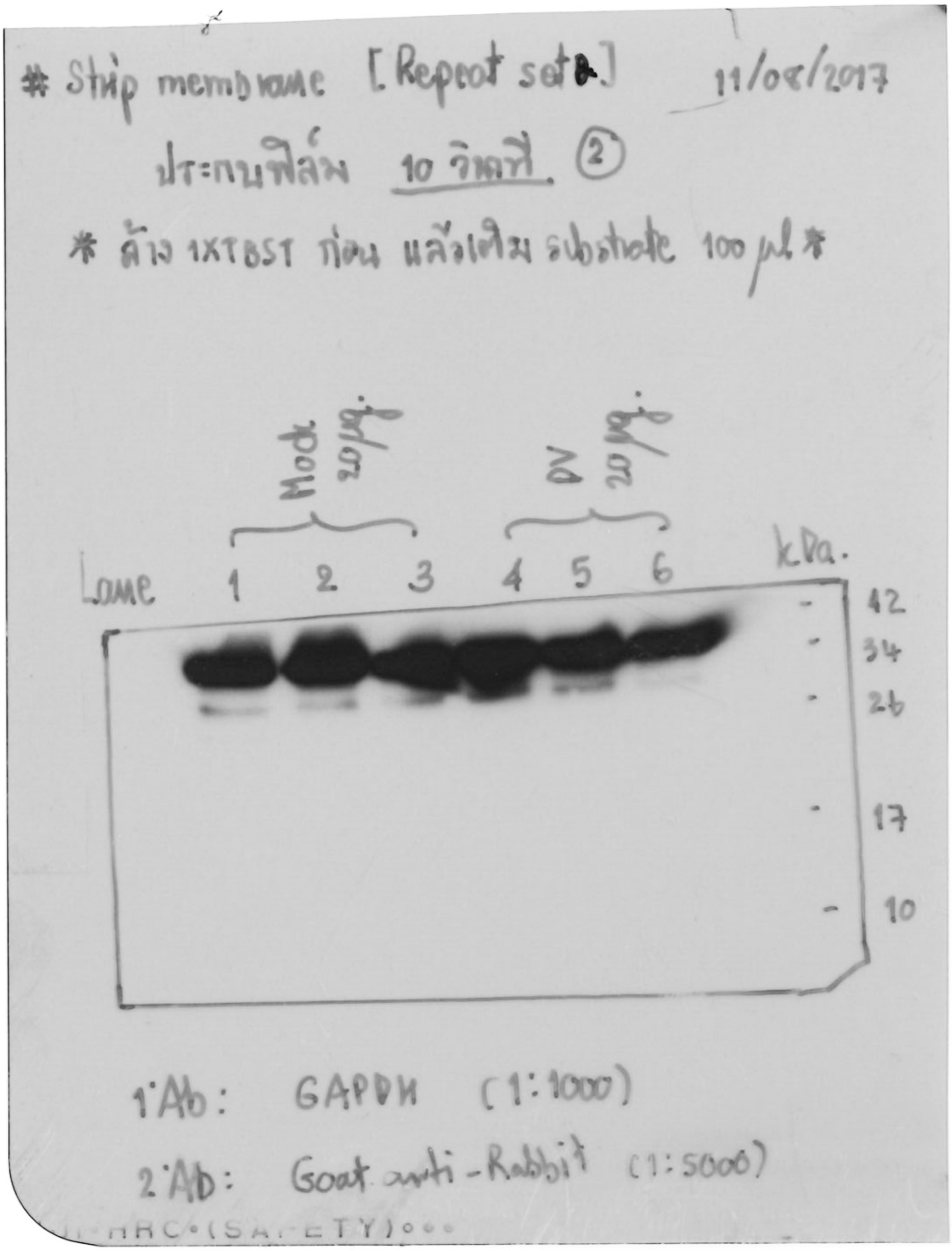

Full uncropped western blot image for figure 3
p-PKM2 Ser37: set 3

Immunoblot: p-PKM2 Ser37

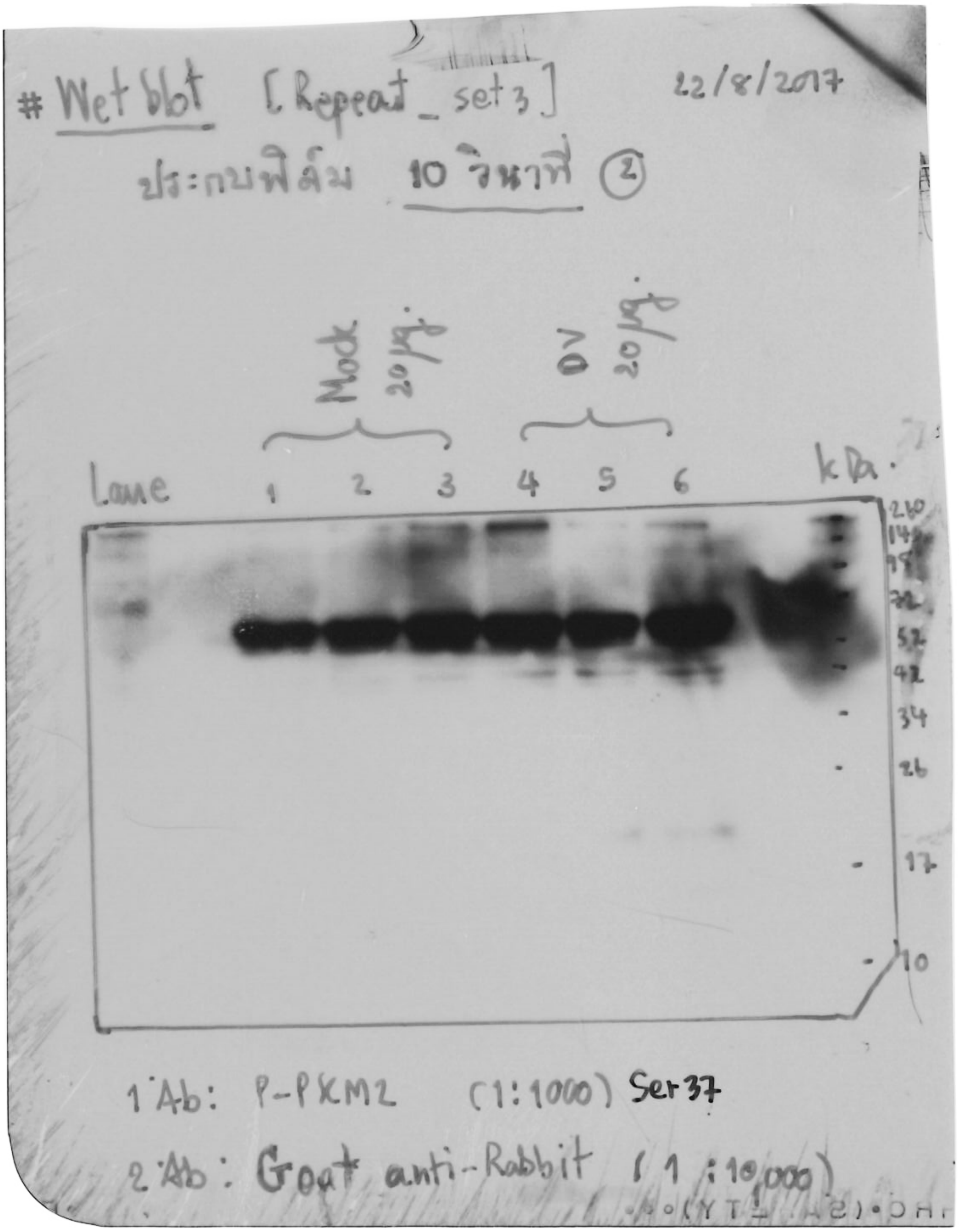

Full uncropped western blot image for figure 3
Total PKM2: set 3

Immunoblot: total PKM2

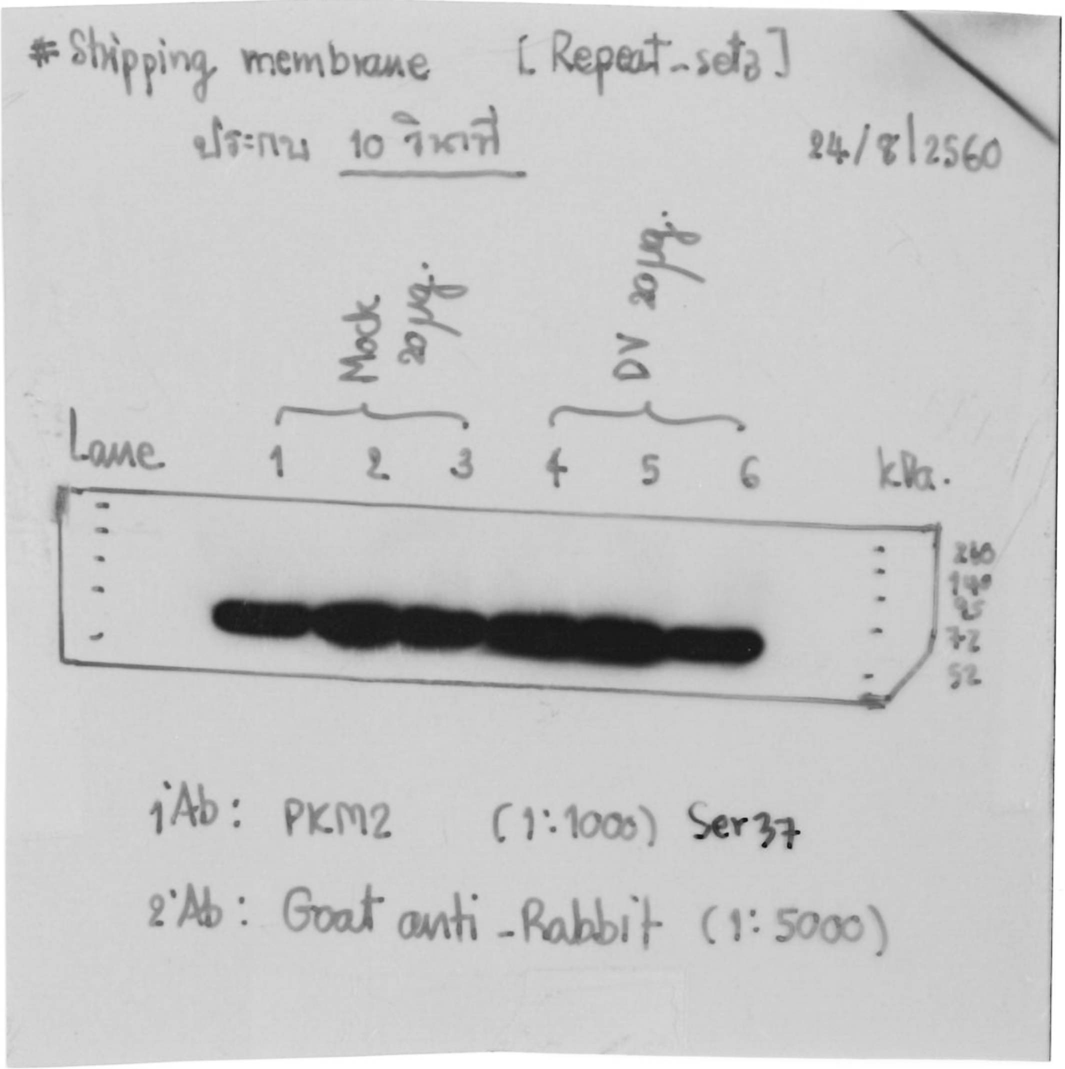

Full uncropped western blot image for figure 3
GAPDH protein: set 3

Immunoblot: GAPDH

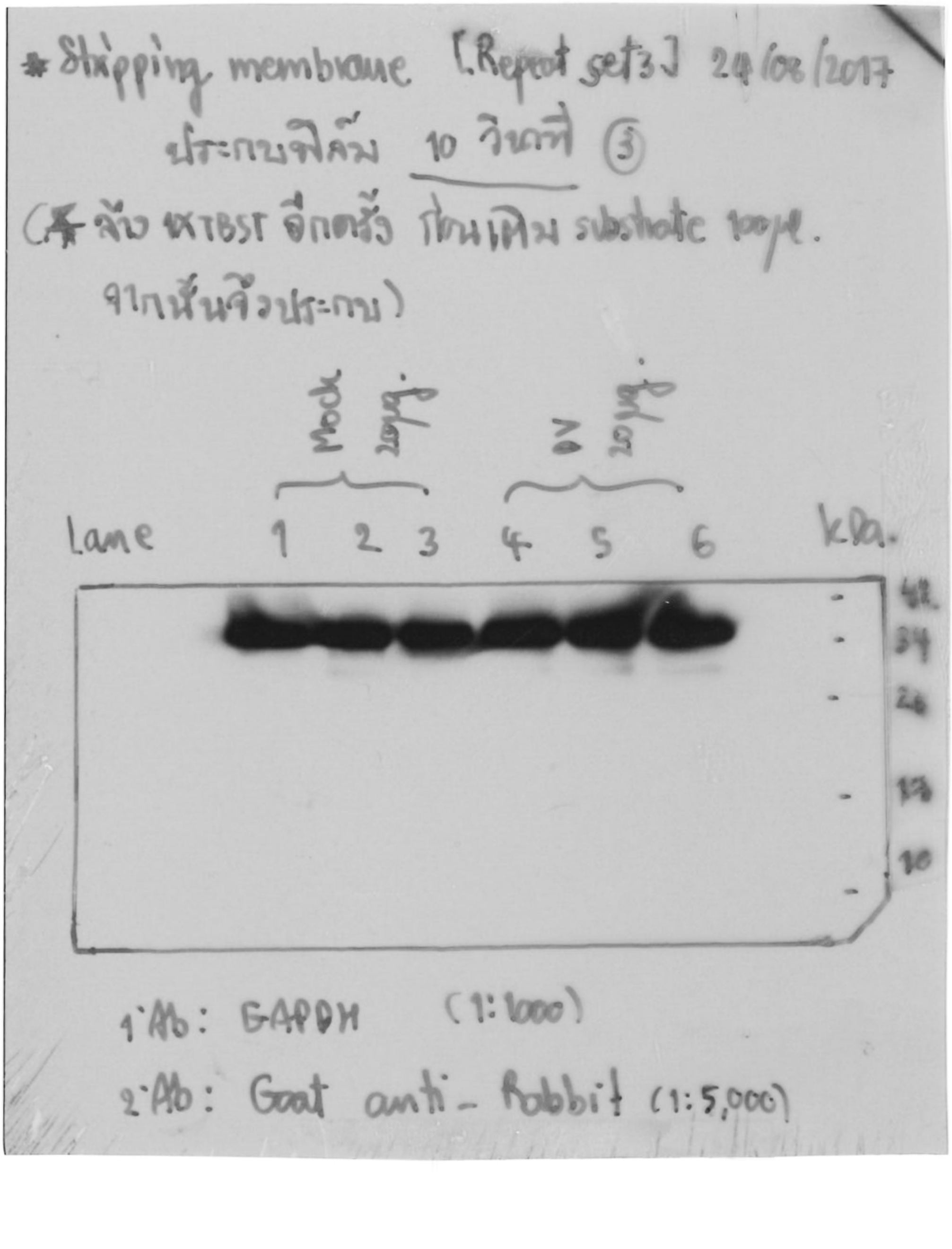

Full uncropped western blot image for figure 5
NS1 protein: set 1 (used in manuscript figure 5A)

Immunoblot: NS1 protein

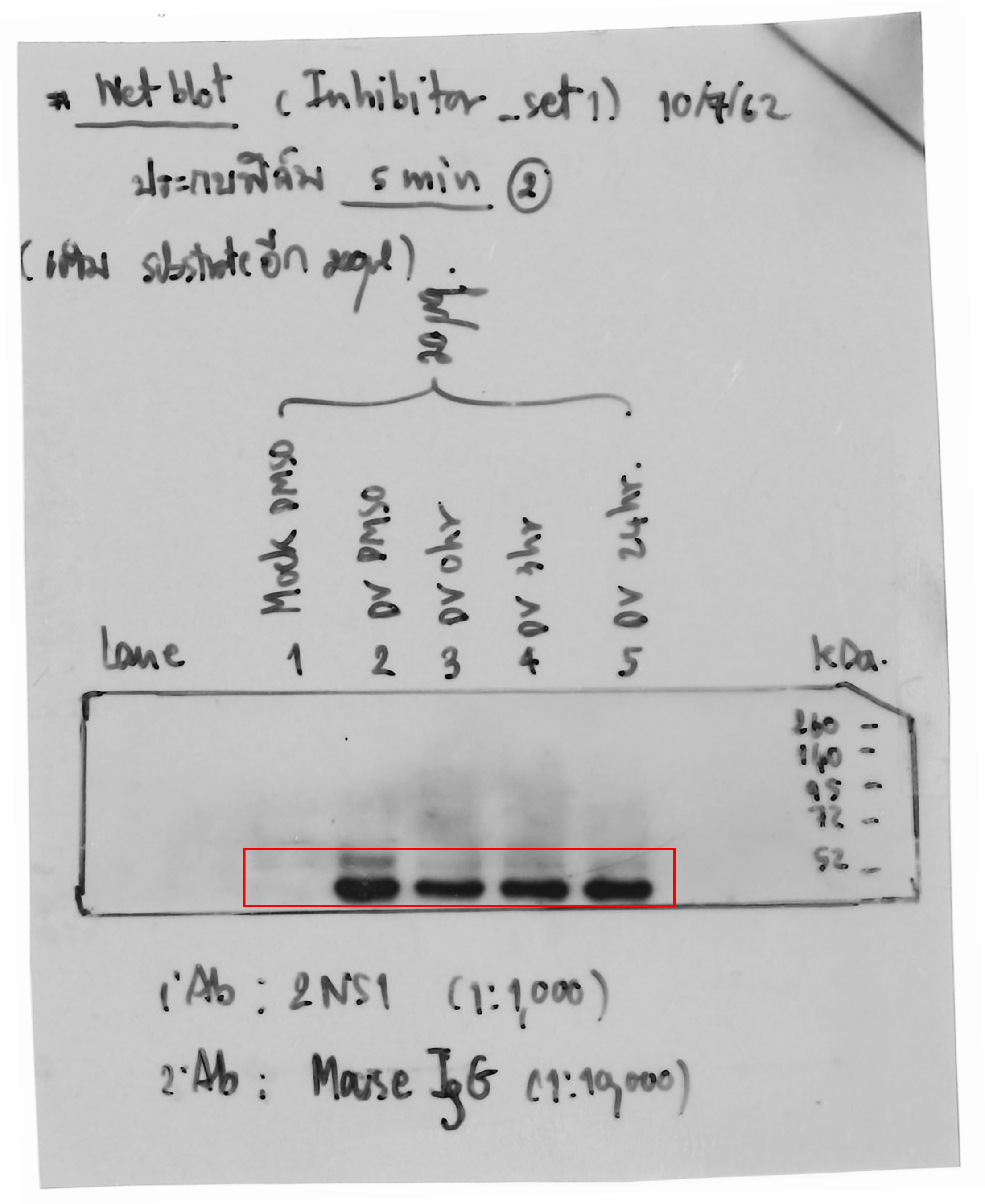

Red box represents the cropped image used in manuscript figure 5A.

Full uncropped western blot image for figure 5
E protein: set 1 (used in manuscript figure 5A)

Immunoblot: E protein

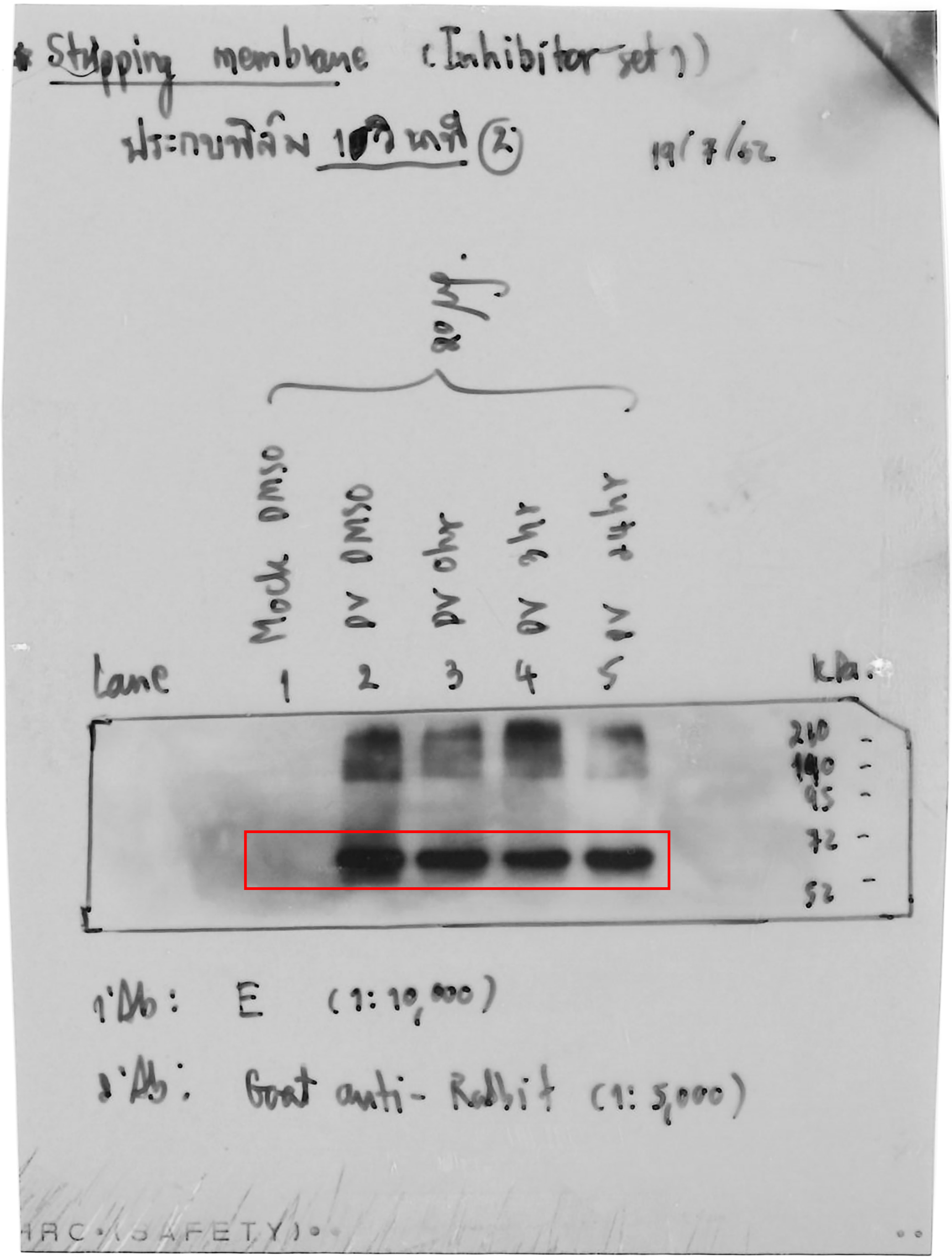

Red box represents the cropped image used in manuscript figure 5A.

Full uncropped western blot image for figure 5
GAPDH protein: set 1 (used in manuscript figure 5A)

Immunoblot: GAPDH

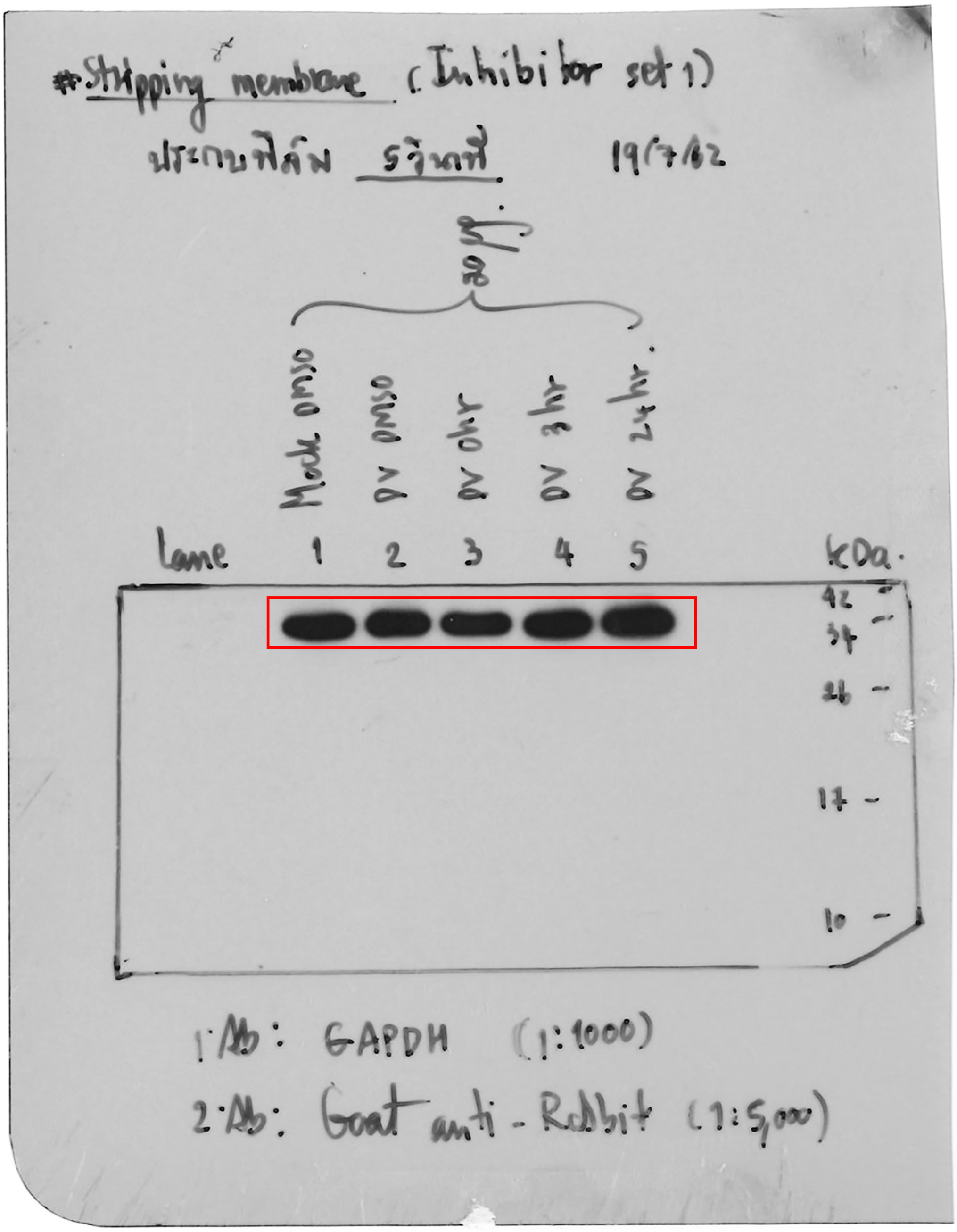

Red box represents the cropped image used in manuscript figure 5A.

Full uncropped western blot image for figure 5
NS1 protein: set 2

Immunoblot: NS1 protein

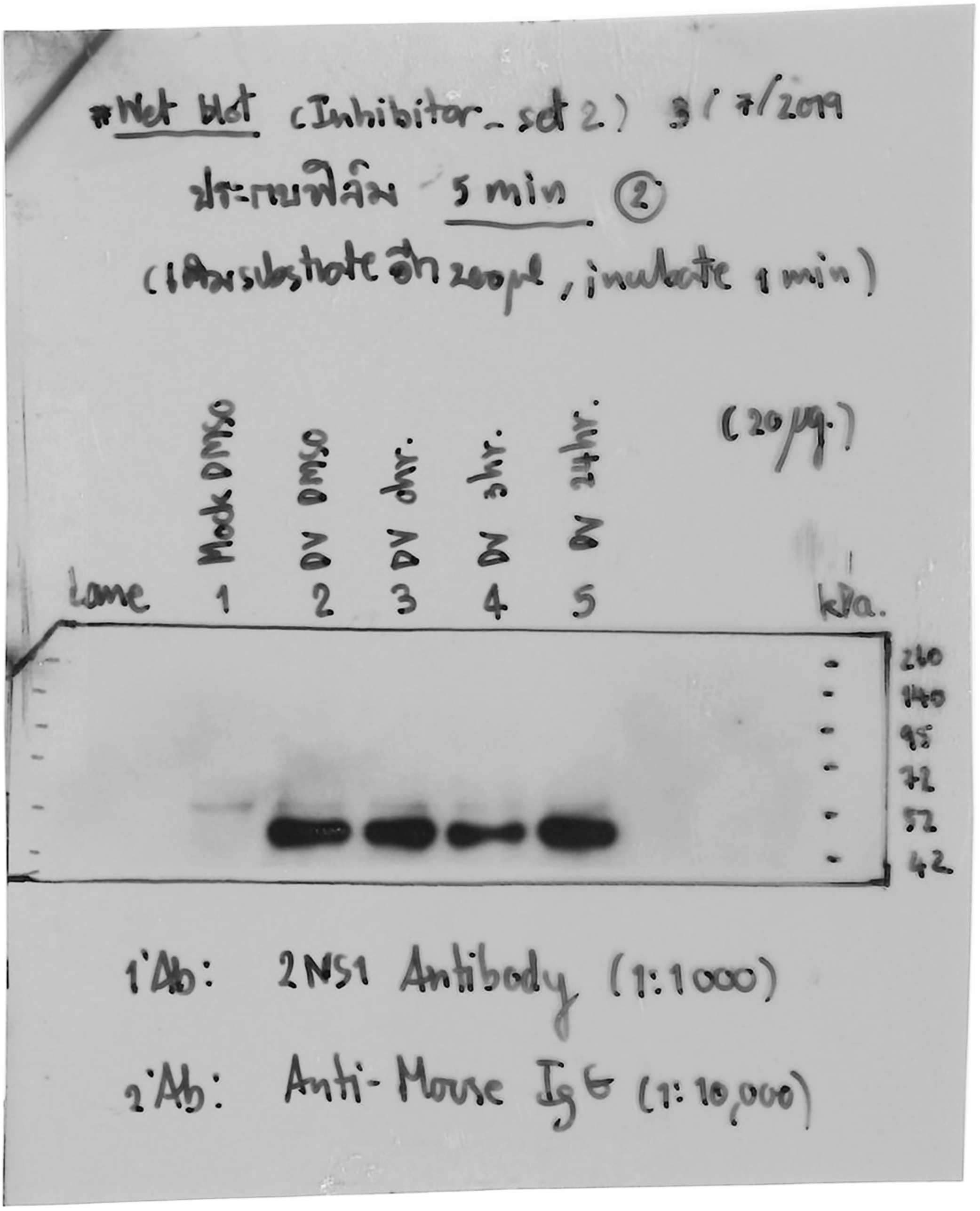

Full uncropped western blot image for figure 5
E protein: set 2

Immunoblot: E protein

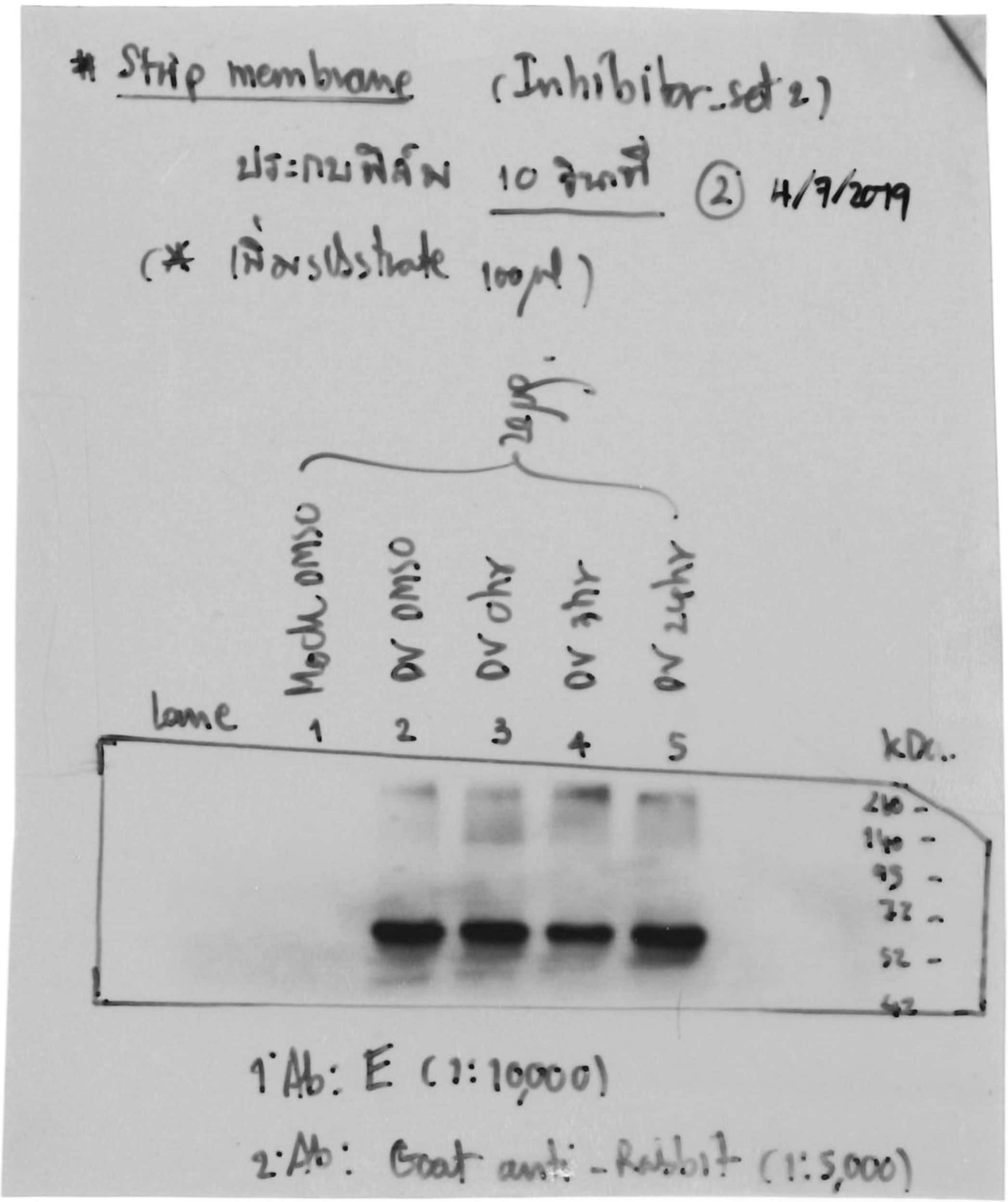

Full uncropped western blot image for figure 5
GAPDH protein: set 2

Immunoblot: GAPDH

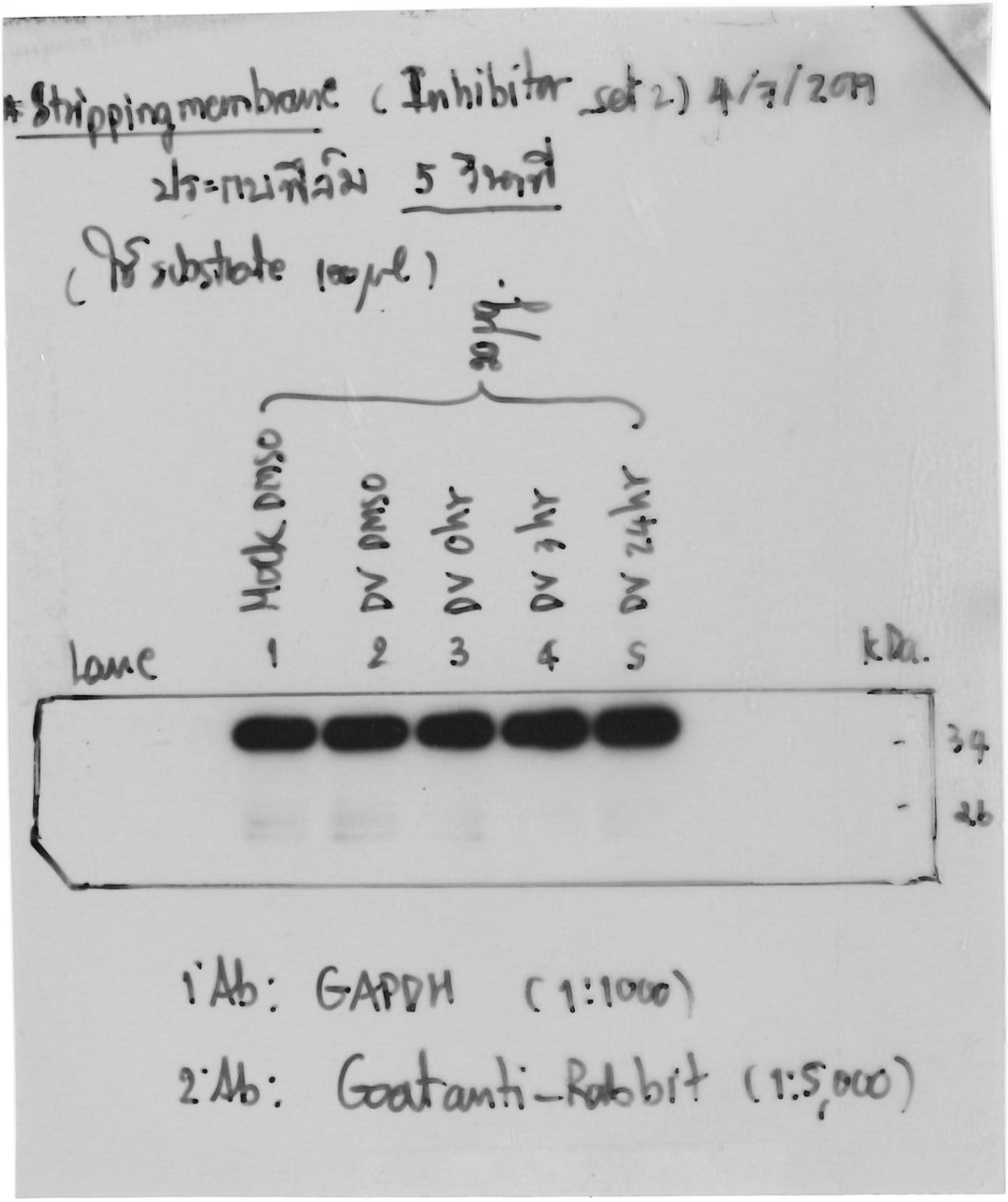

Full uncropped western blot image for figure 5
NS1 protein: set 3

Immunoblot: NS1 protein

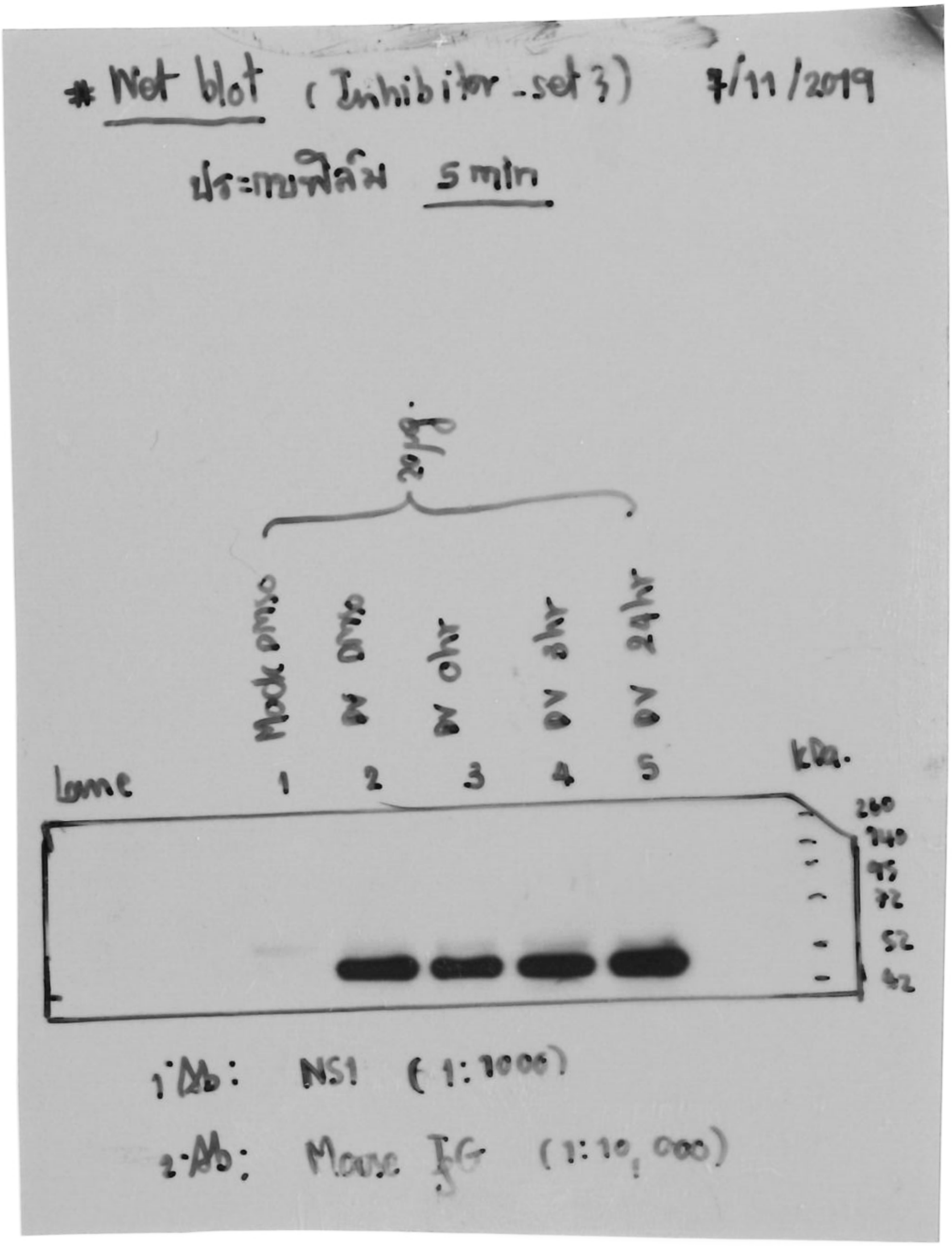

Full uncropped western blot image for figure 5
E protein: set 3

Immunoblot: E protein

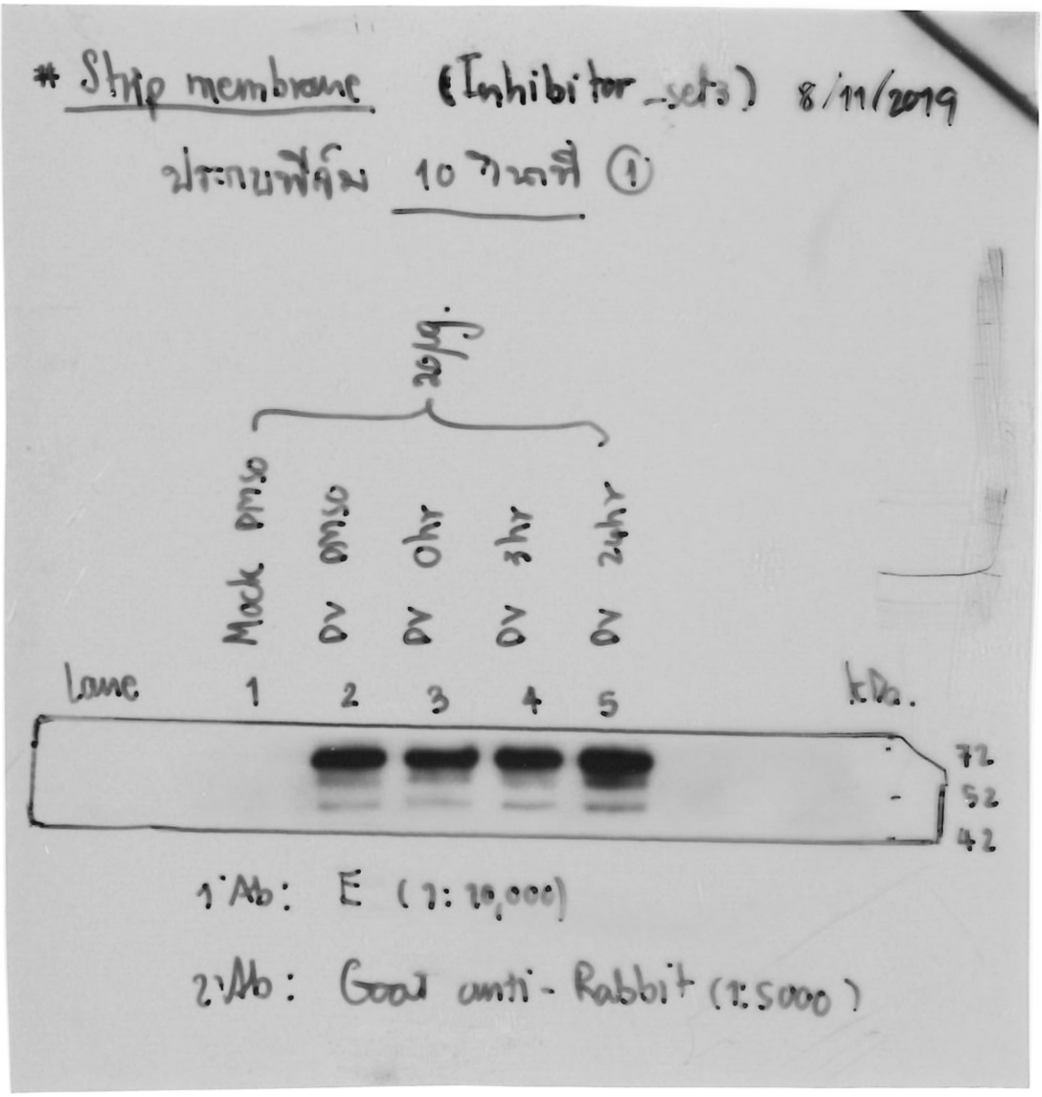

Full uncropped western blot image for figure 5
GAPDH protein: set 3

Immunoblot: GAPDH

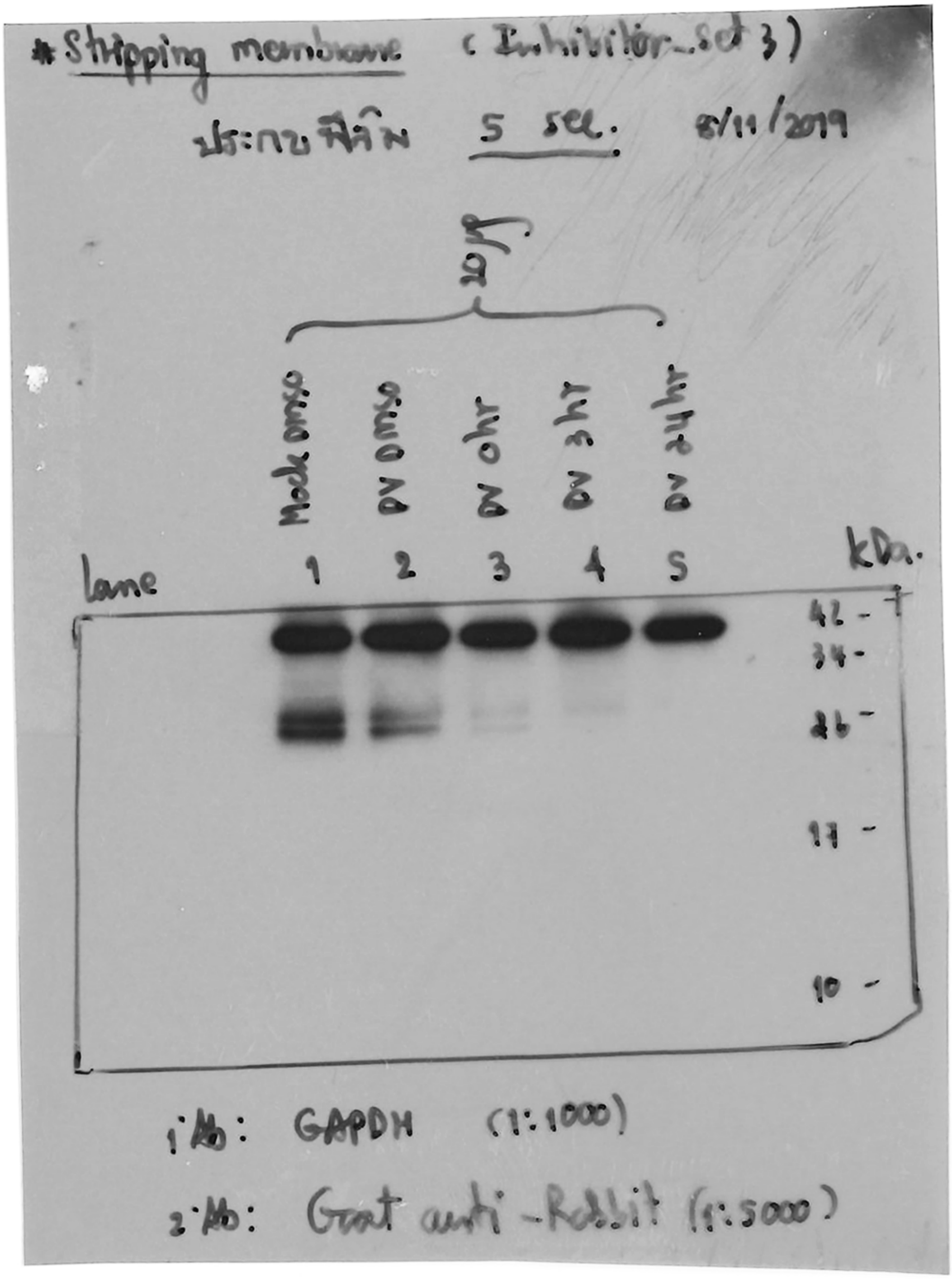

Full uncropped western blot image for figure 5
NS5 protein: set 1 (used in manuscript figure 5B)

Immunoblot: NS5 protein

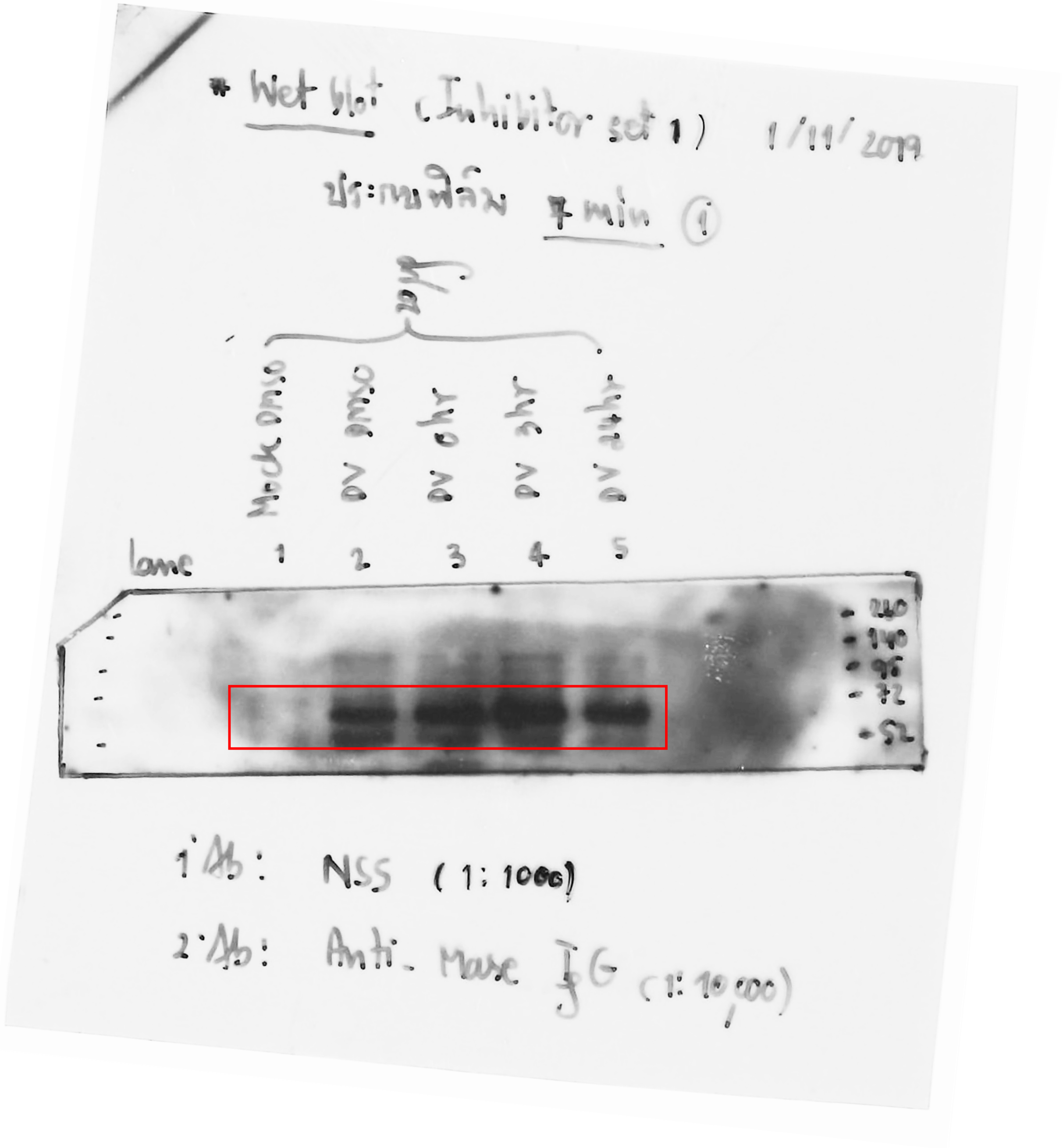

Red box represents the cropped image used in manuscript figure 5B.

Full uncropped western blot image for figure 5
GAPDH protein: set 1 (used in manuscript figure 5B)

Immunoblot: GAPDH

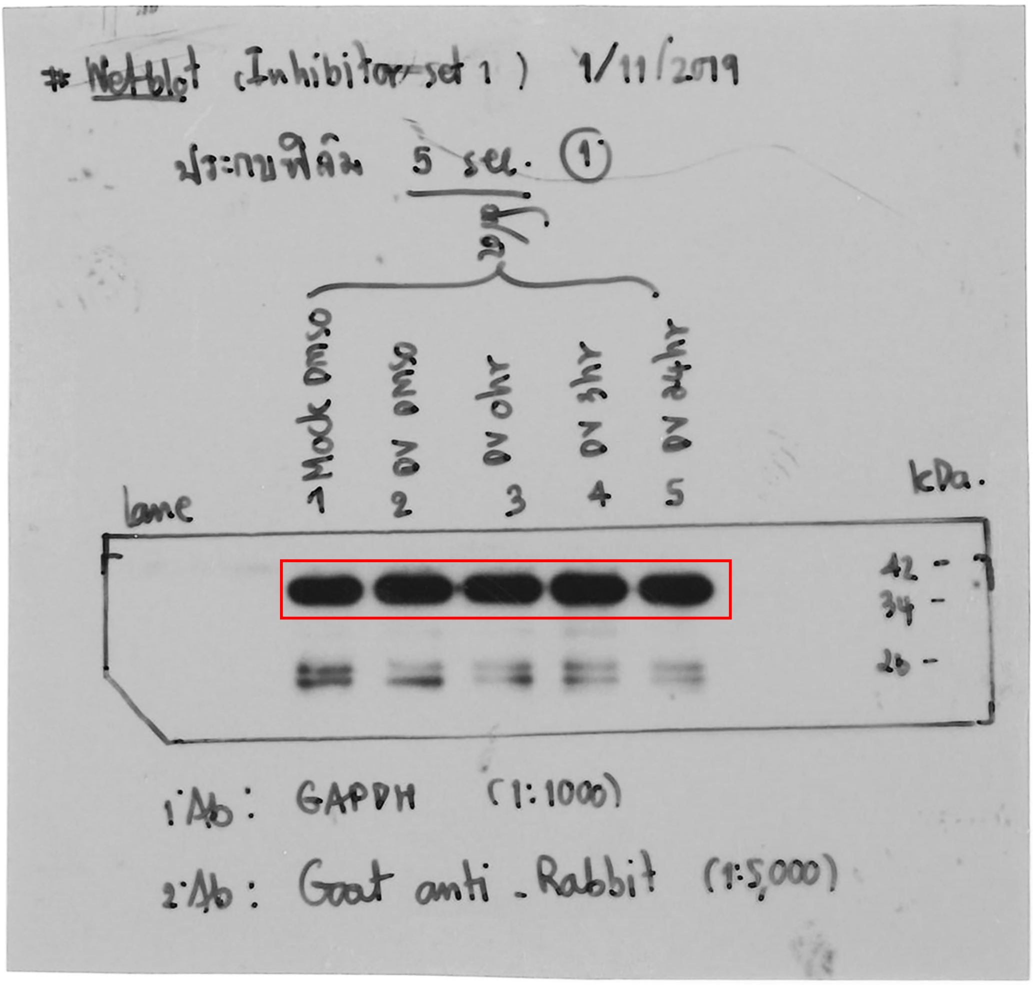

Red box represents the cropped image used in manuscript figure 5B.

Full uncropped western blot image for figure 5
NS5 protein: set 2

Immunoblot: NS5 protein

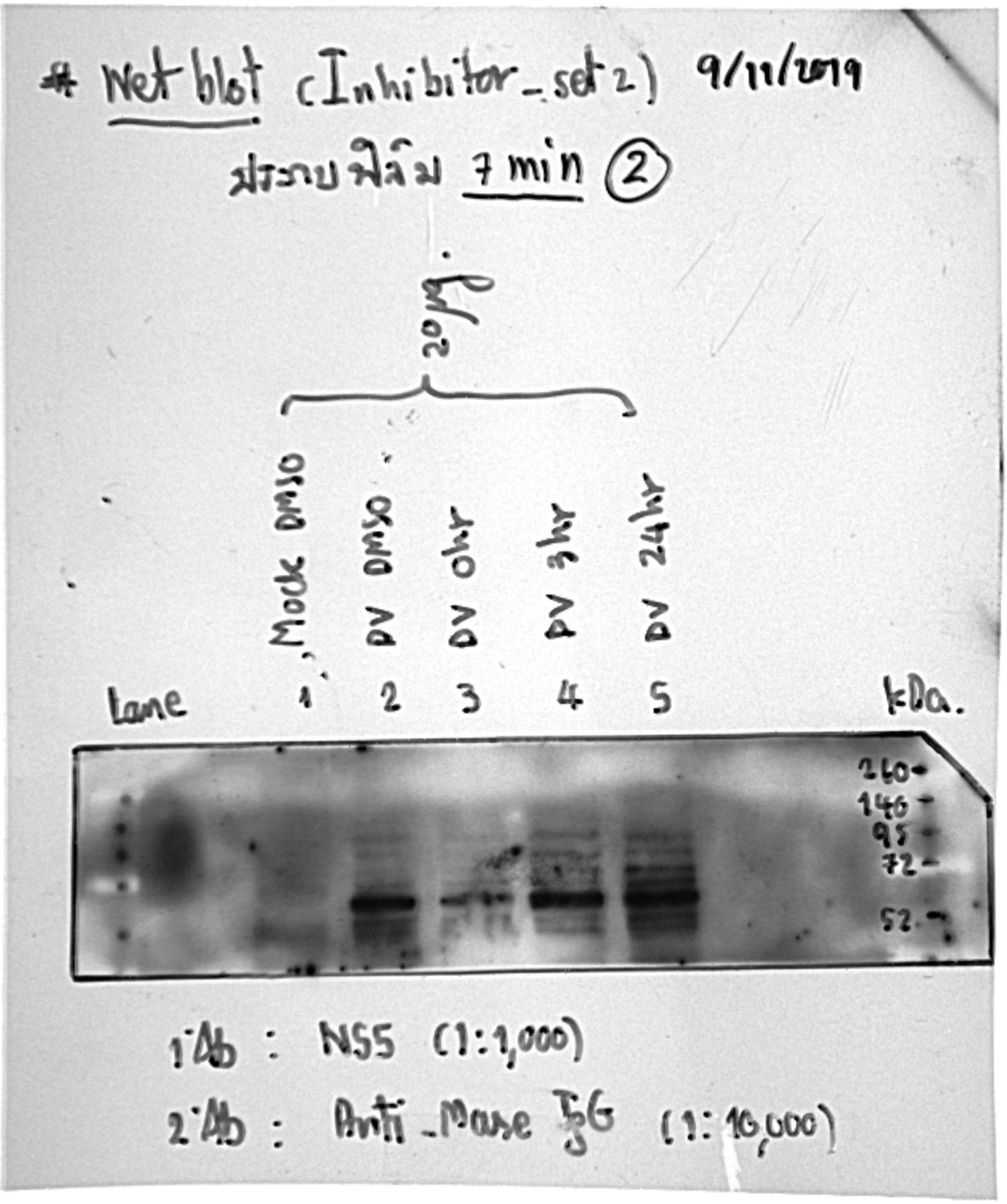

Full uncropped western blot image for figure 5
GAPDH protein: set 2

Immunoblot: GAPDH

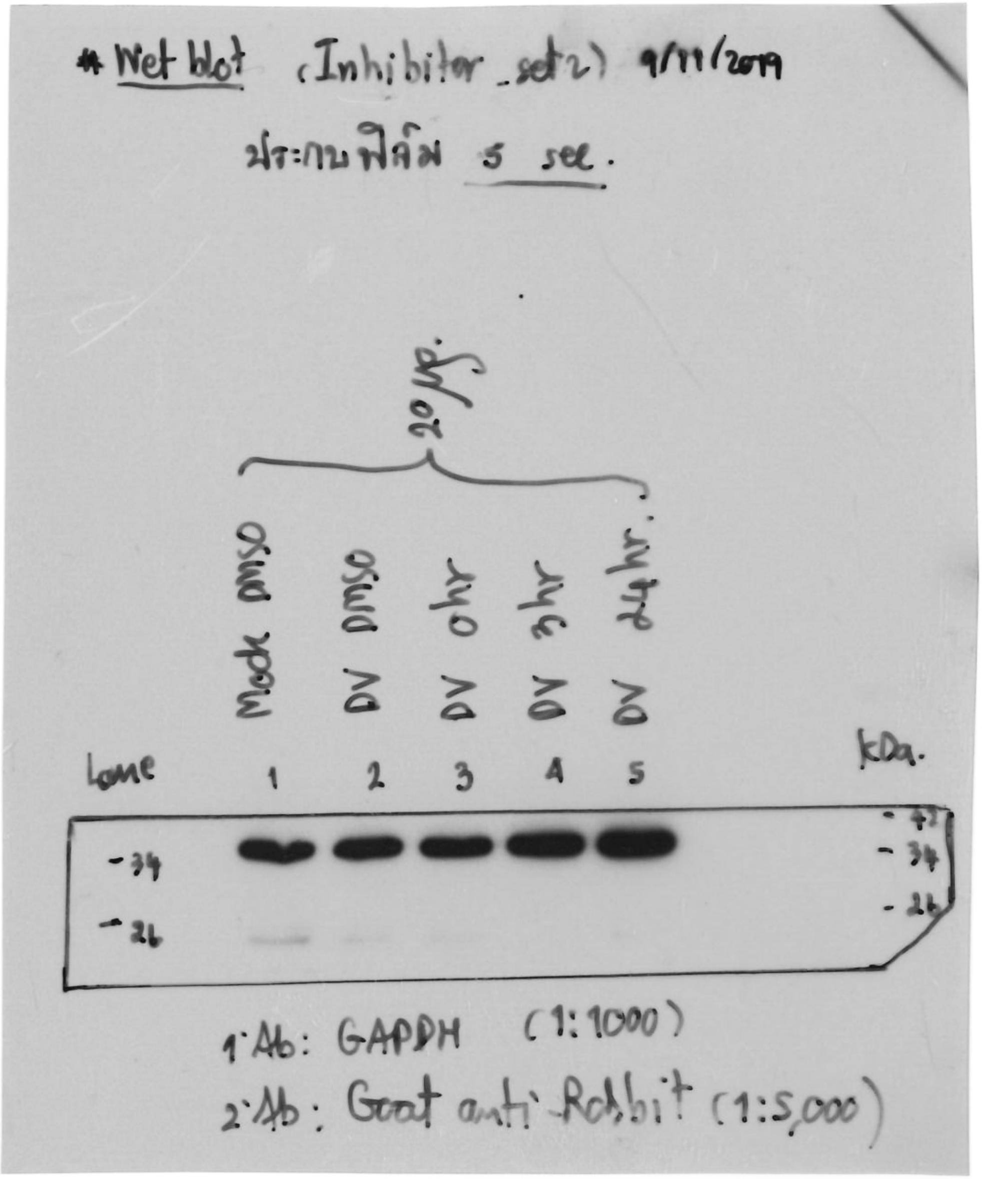

Full uncropped western blot image for figure 5
NS5 protein: set 3

Immunoblot: NS5 protein

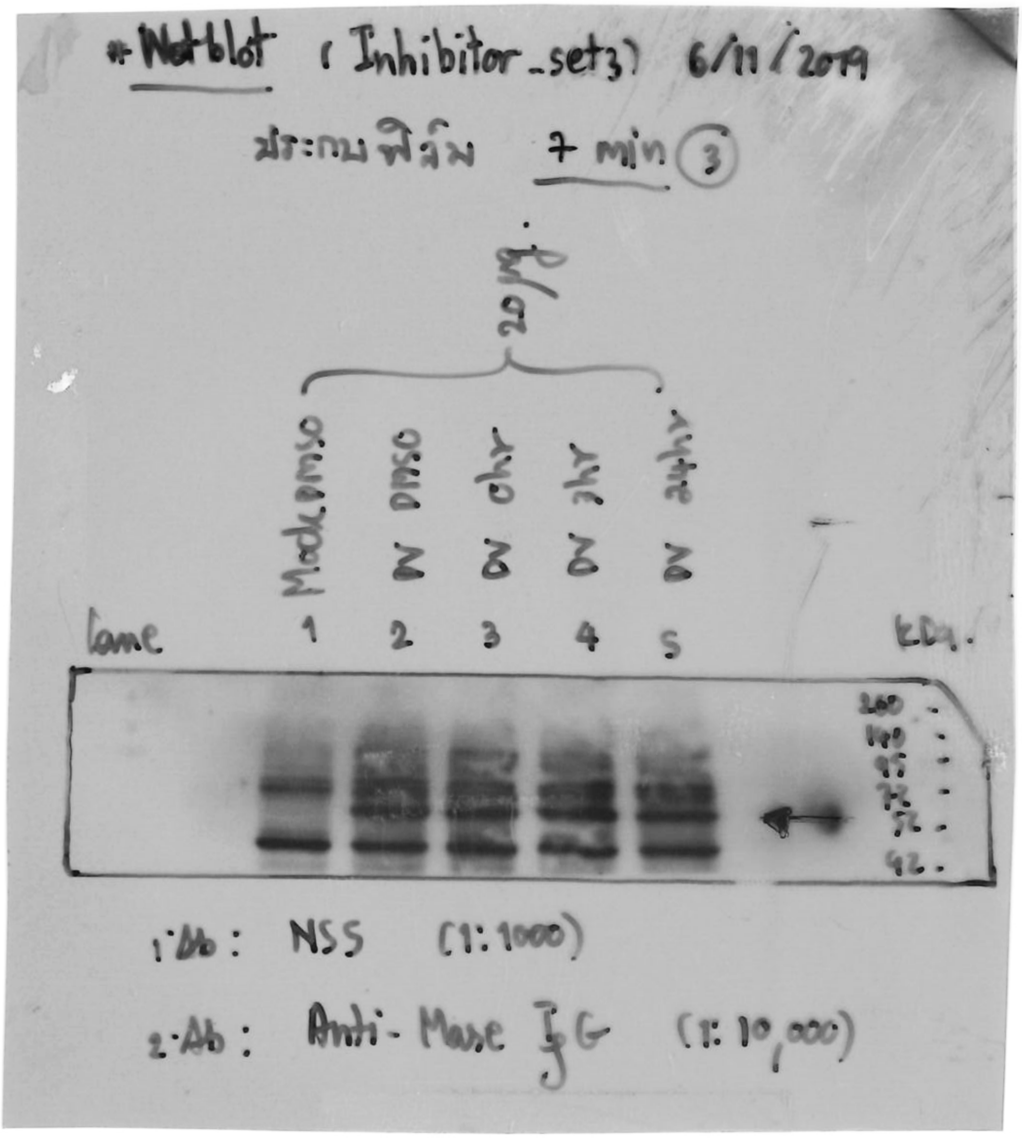

Full uncropped western blot image for figure 5
GAPDH protein: set 3

Immunoblot: GAPDH

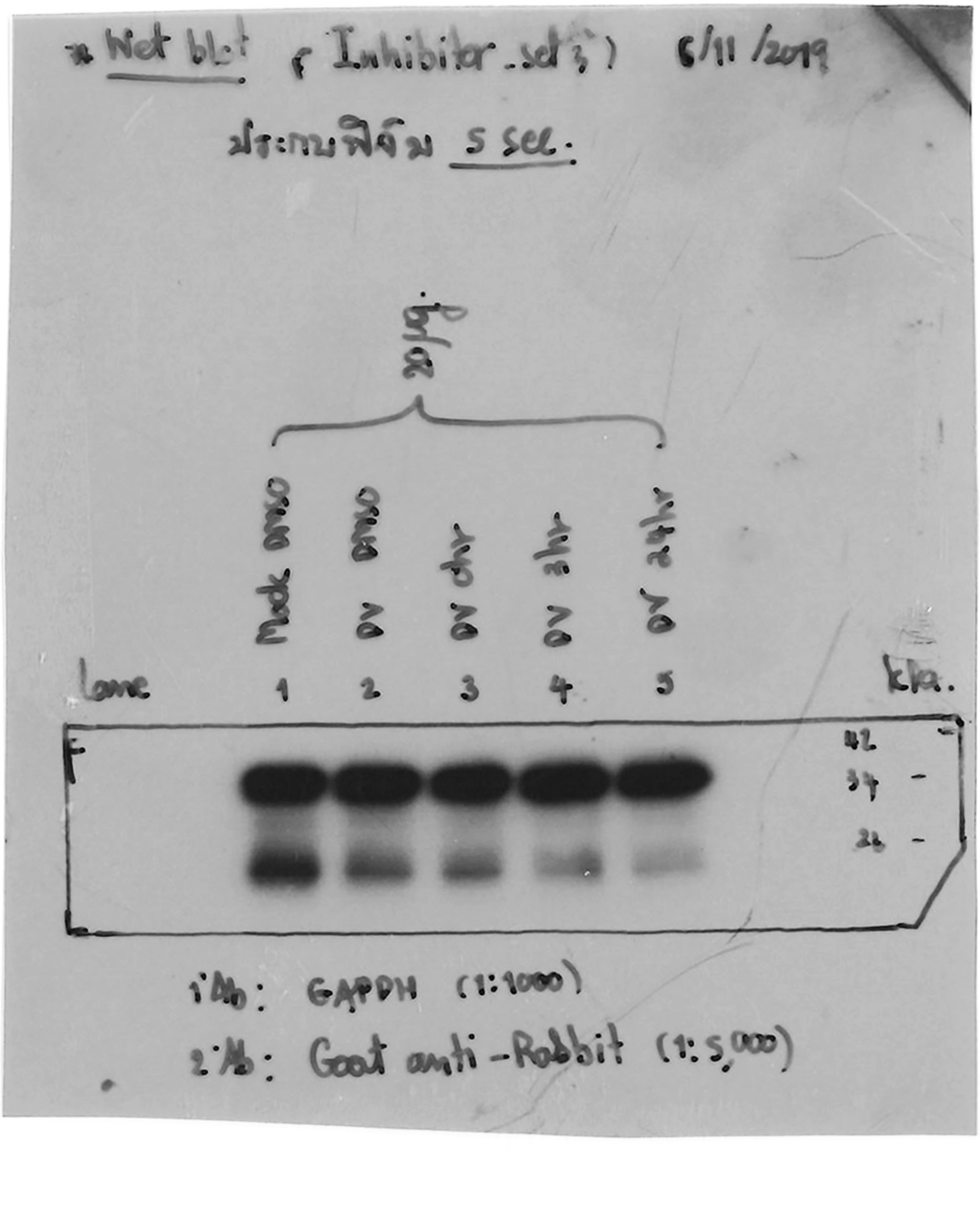

Full uncropped western blot image for figure 6
NS1 protein: set 1 (used in manuscript figure 6A)

Immunoblot: NS1 protein

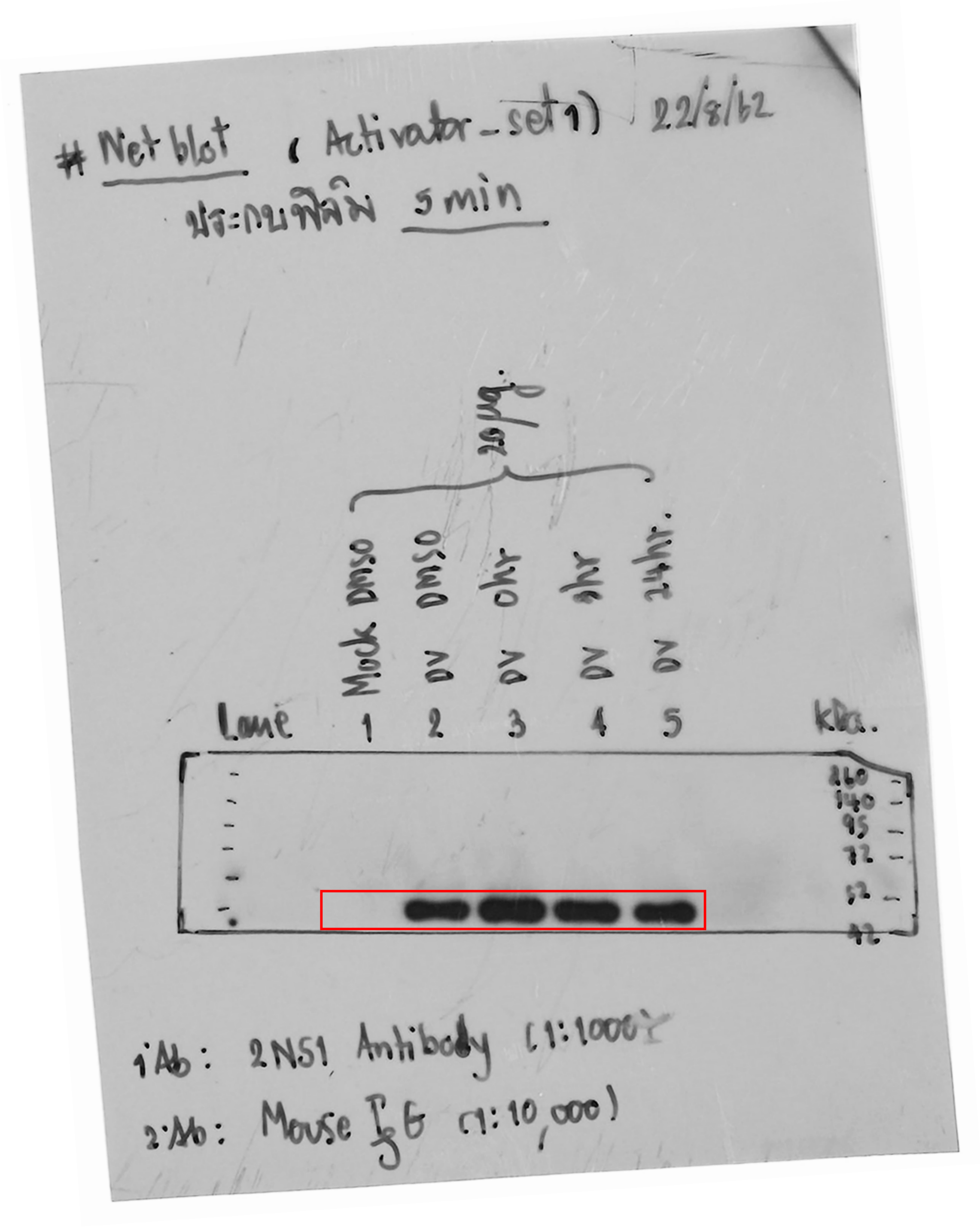

Red box represents the cropped image used in manuscript figure 6A.

Full uncropped western blot image for figure 6
E protein: set 1 (used in manuscript figure 6A)

Immunoblot: E protein

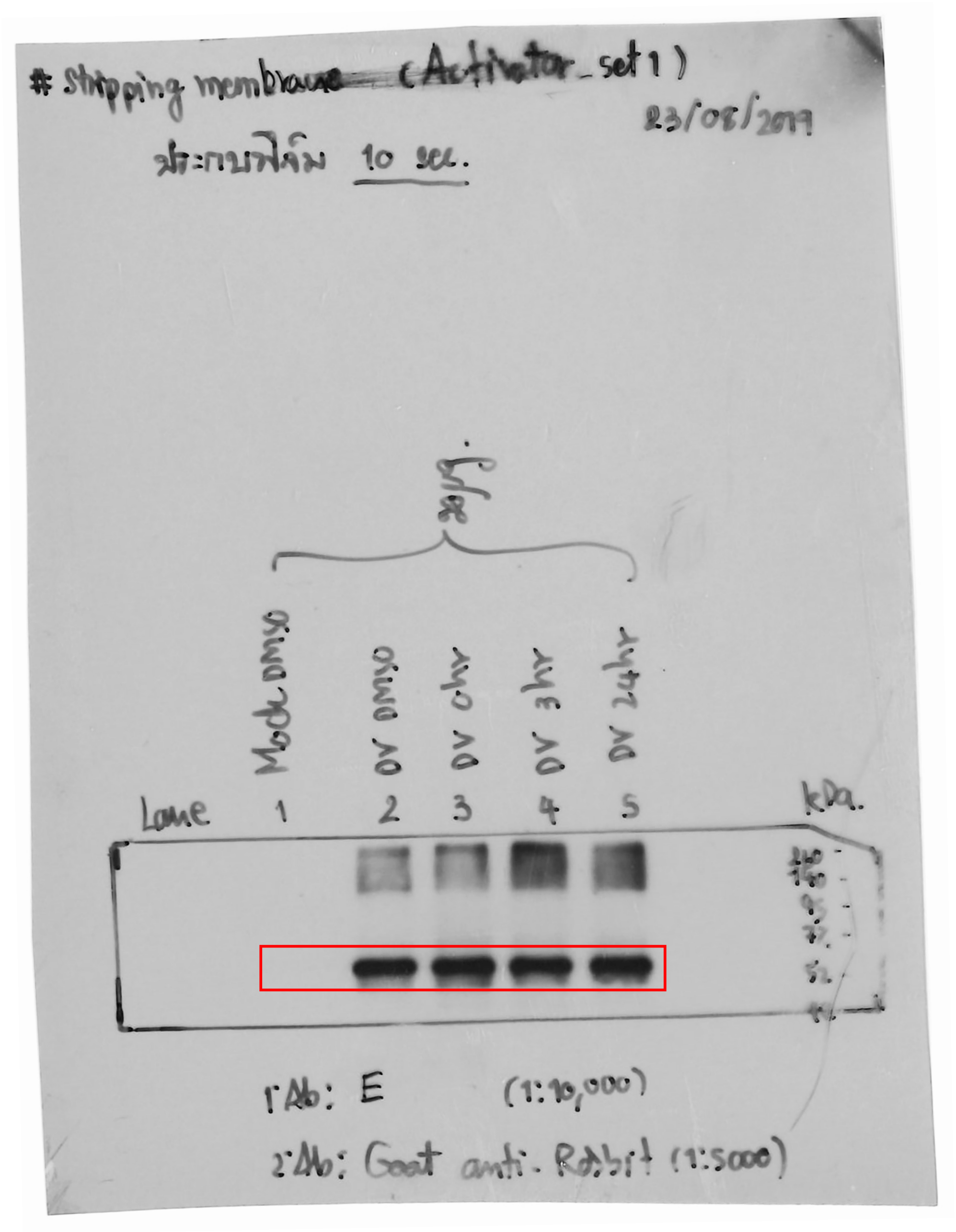

Red box represents the cropped image used in manuscript figure 6A.

Full uncropped western blot image for figure 6
GAPDH protein: set 1 (used in manuscript figure 6A)

Immunoblot: GAPDH

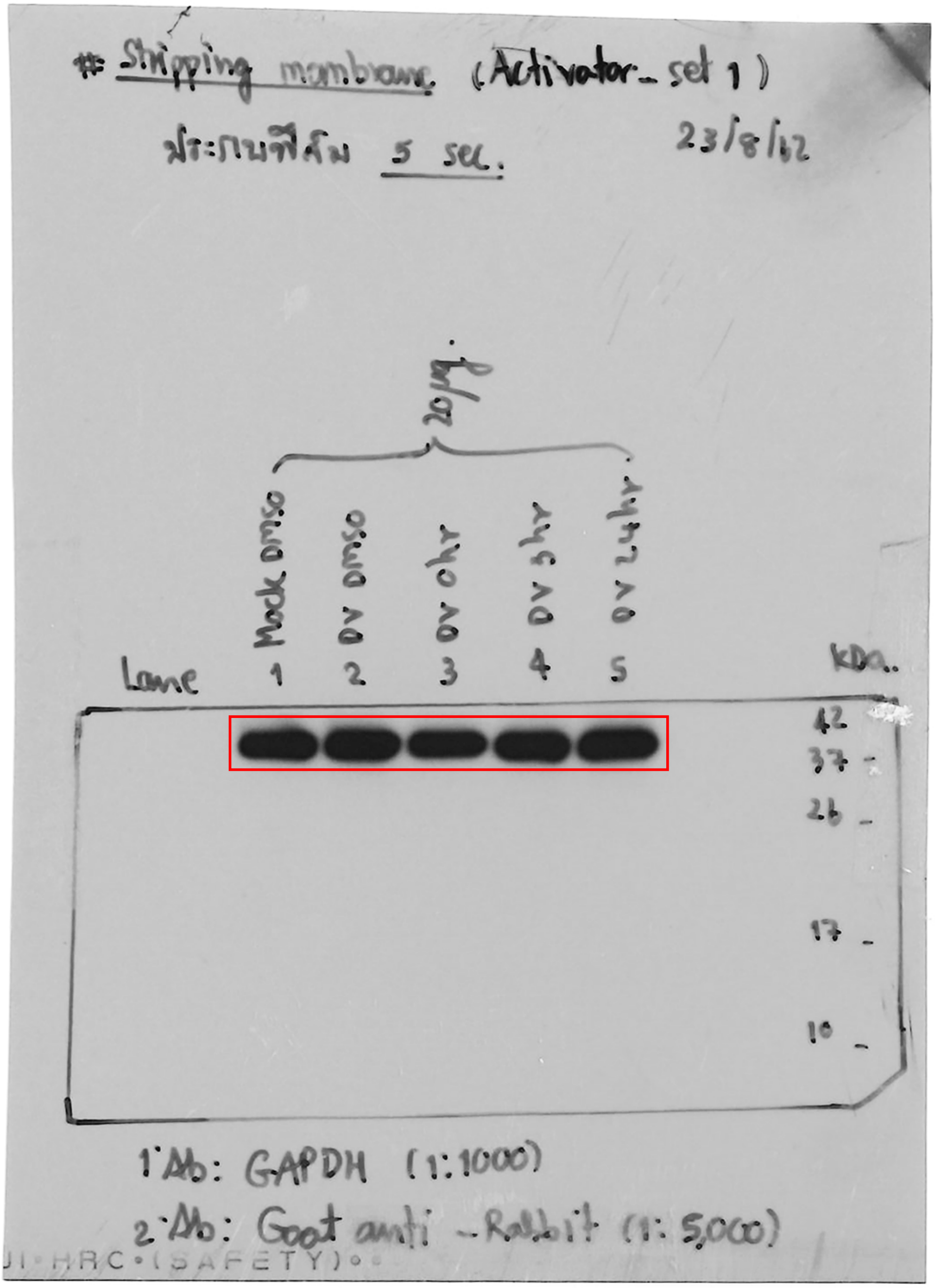

Red box represents the cropped image used in manuscript figure 6A.

Full uncropped western blot image for figure 6
NS1 protein: set 2

Immunoblot: NS1 protein

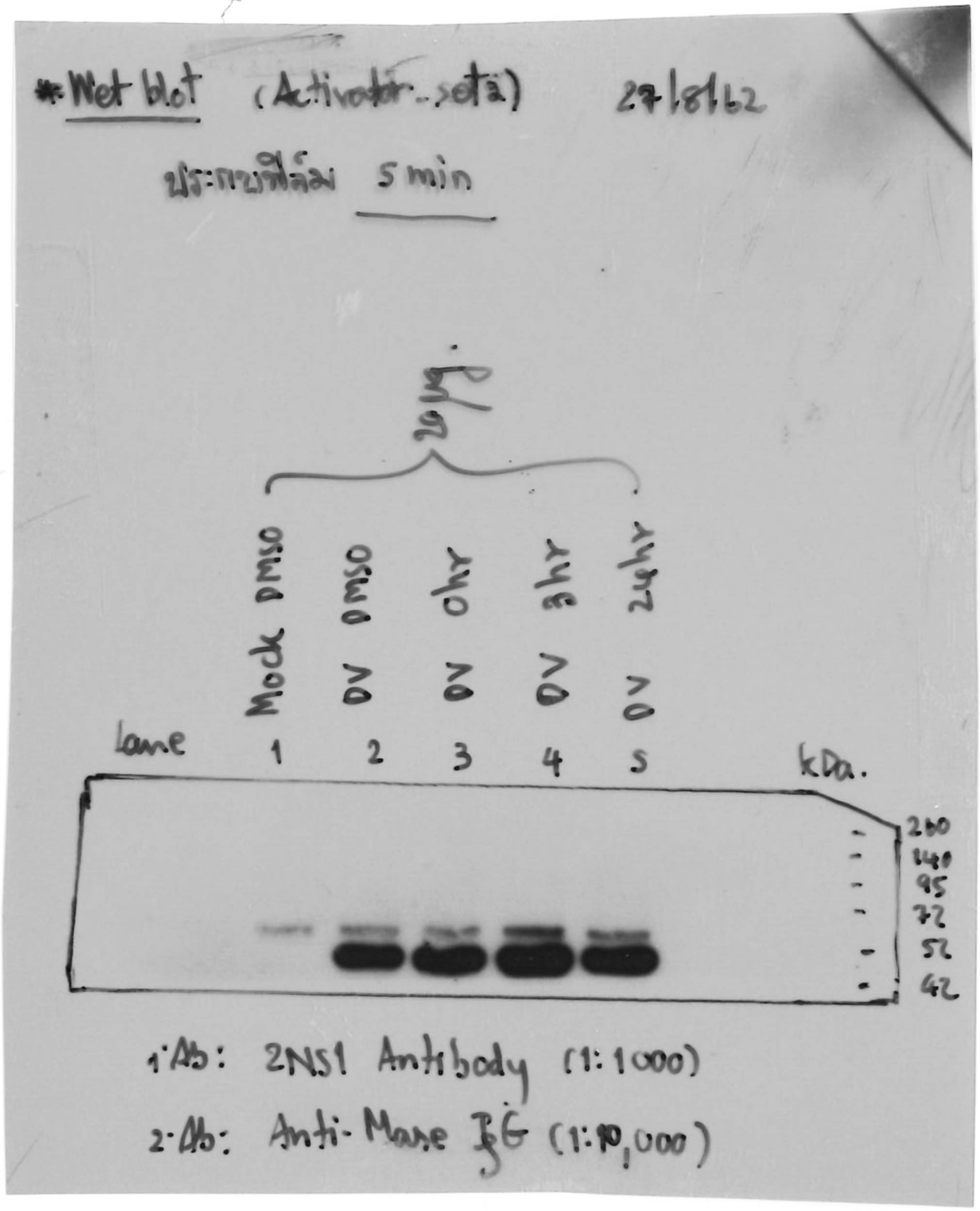

Full uncropped western blot image for figure 6
E protein: set 2

Immunoblot: E protein

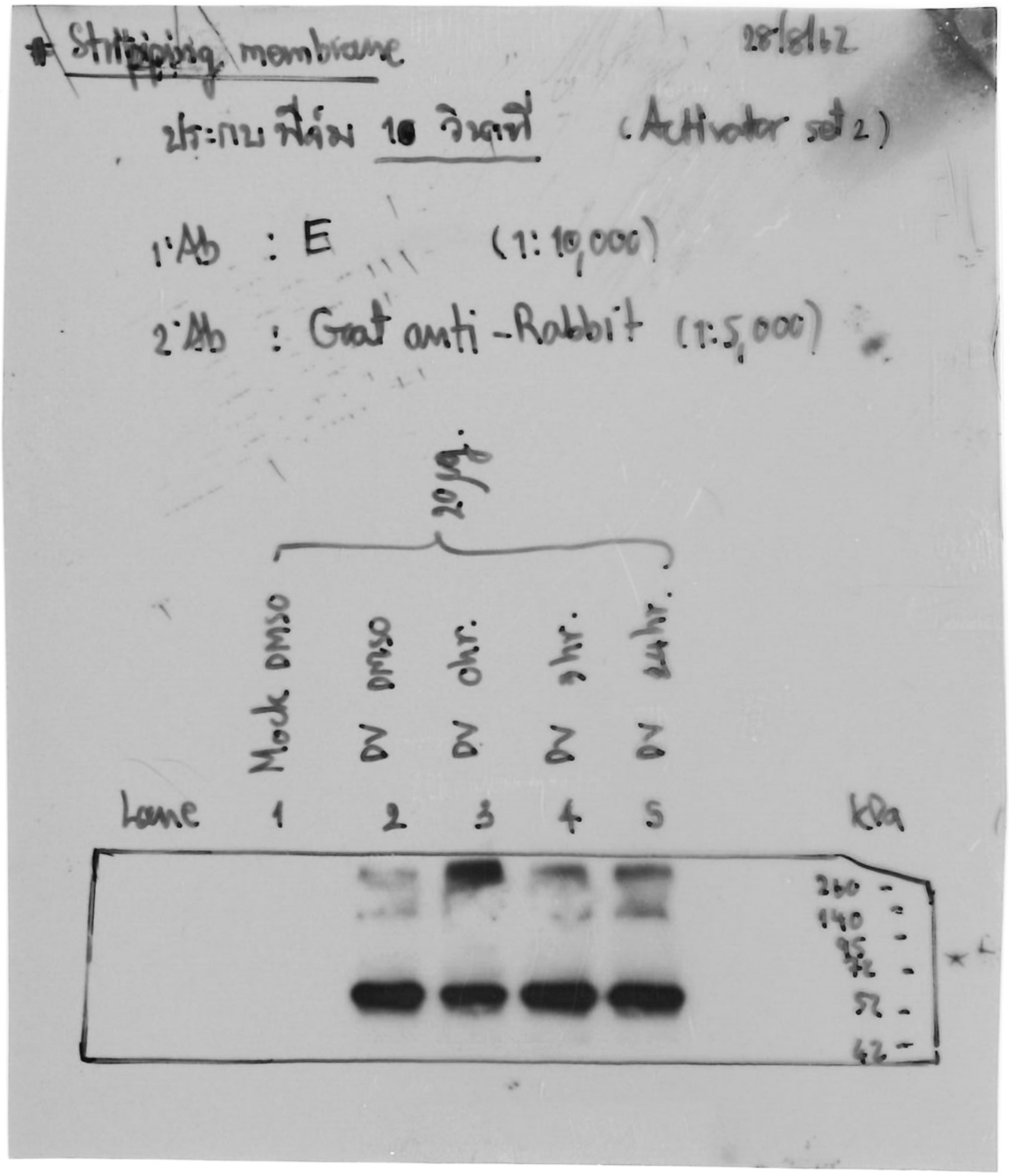

Full uncropped western blot image for figure 6
GAPDH protein: set 2

Immunoblot: GAPDH

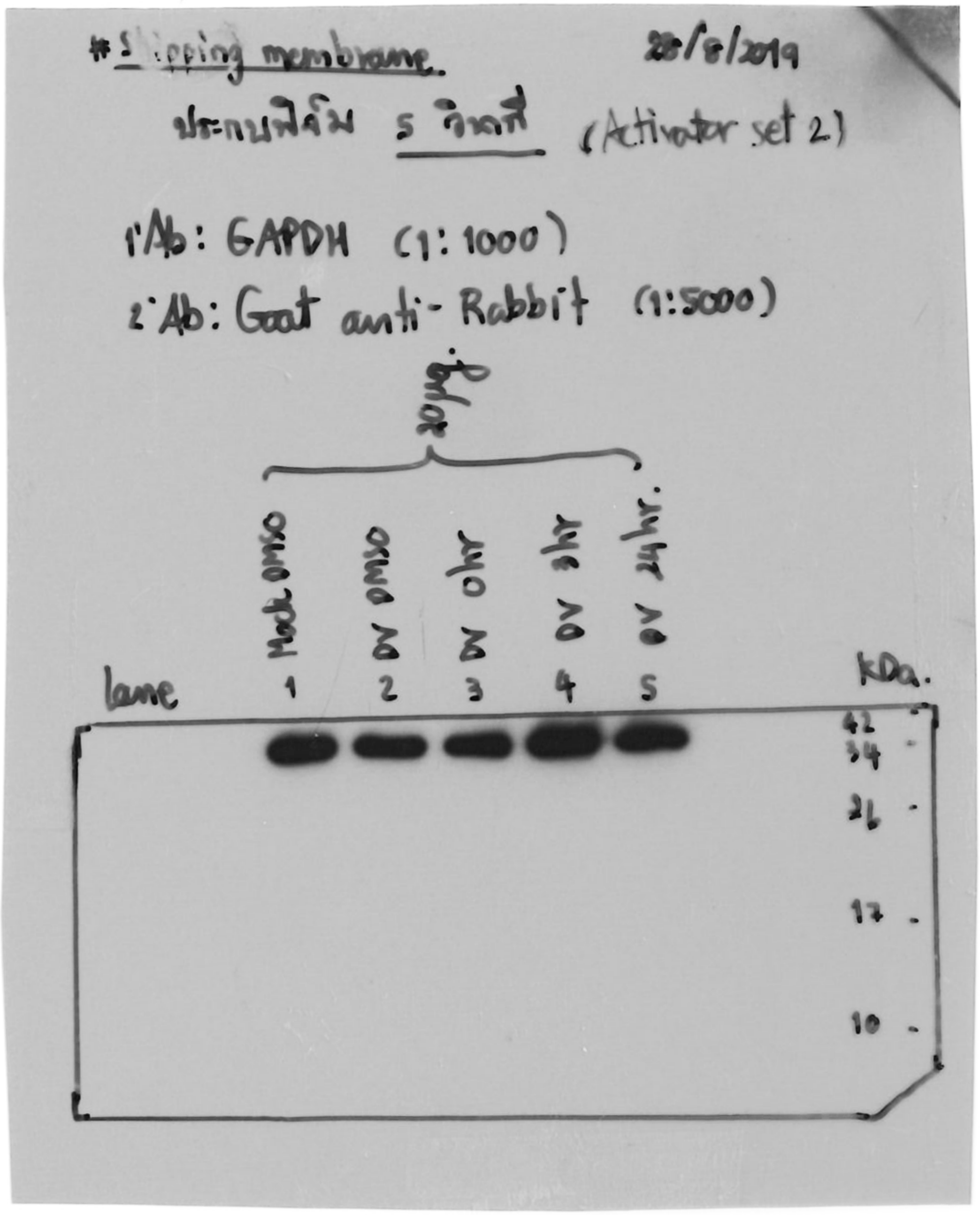

Full uncropped western blot image for figure 6
NS1 protein: set 3

Immunoblot: NS1 protein

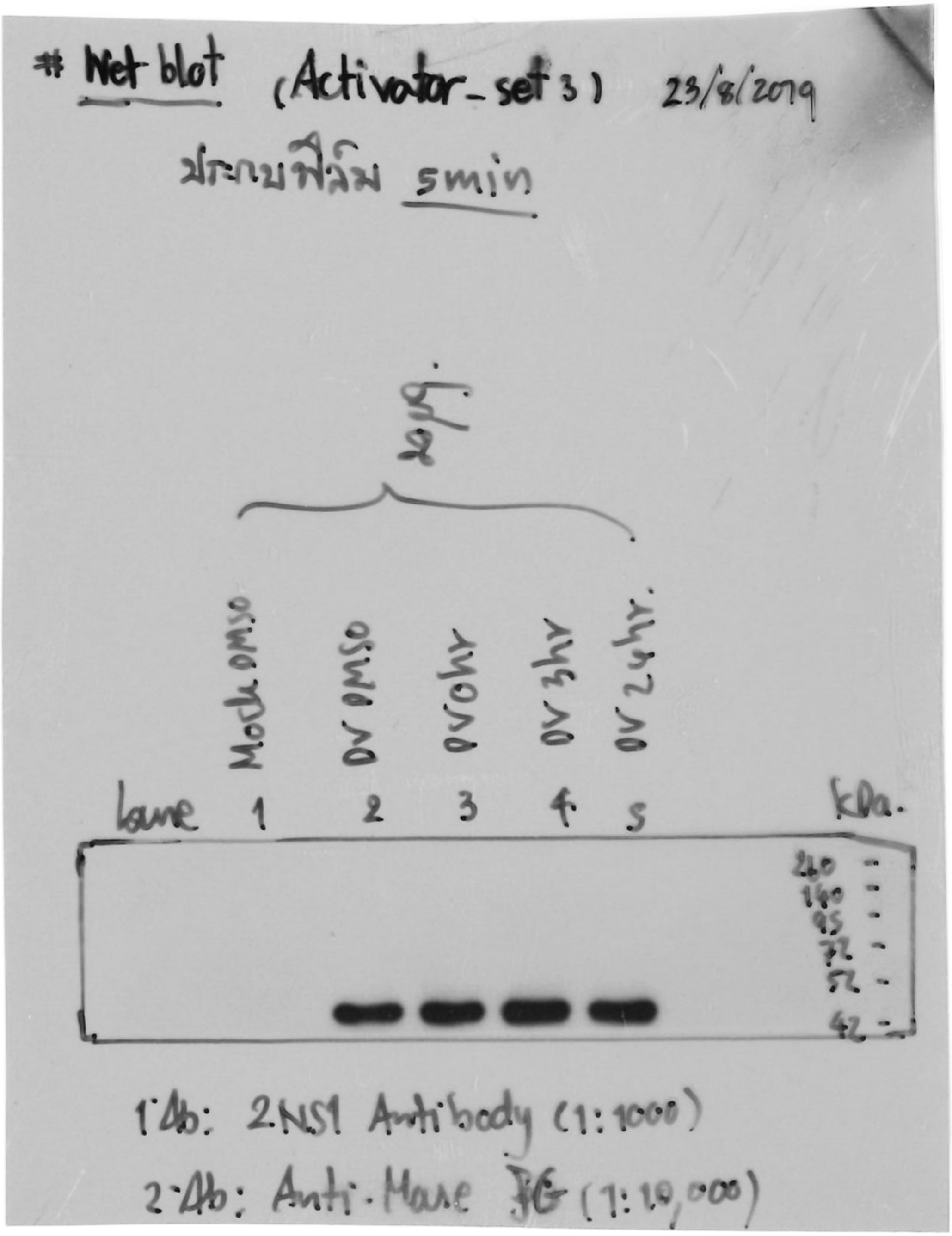

Full uncropped western blot image for figure 6
E protein: set 3

Immunoblot: E protein

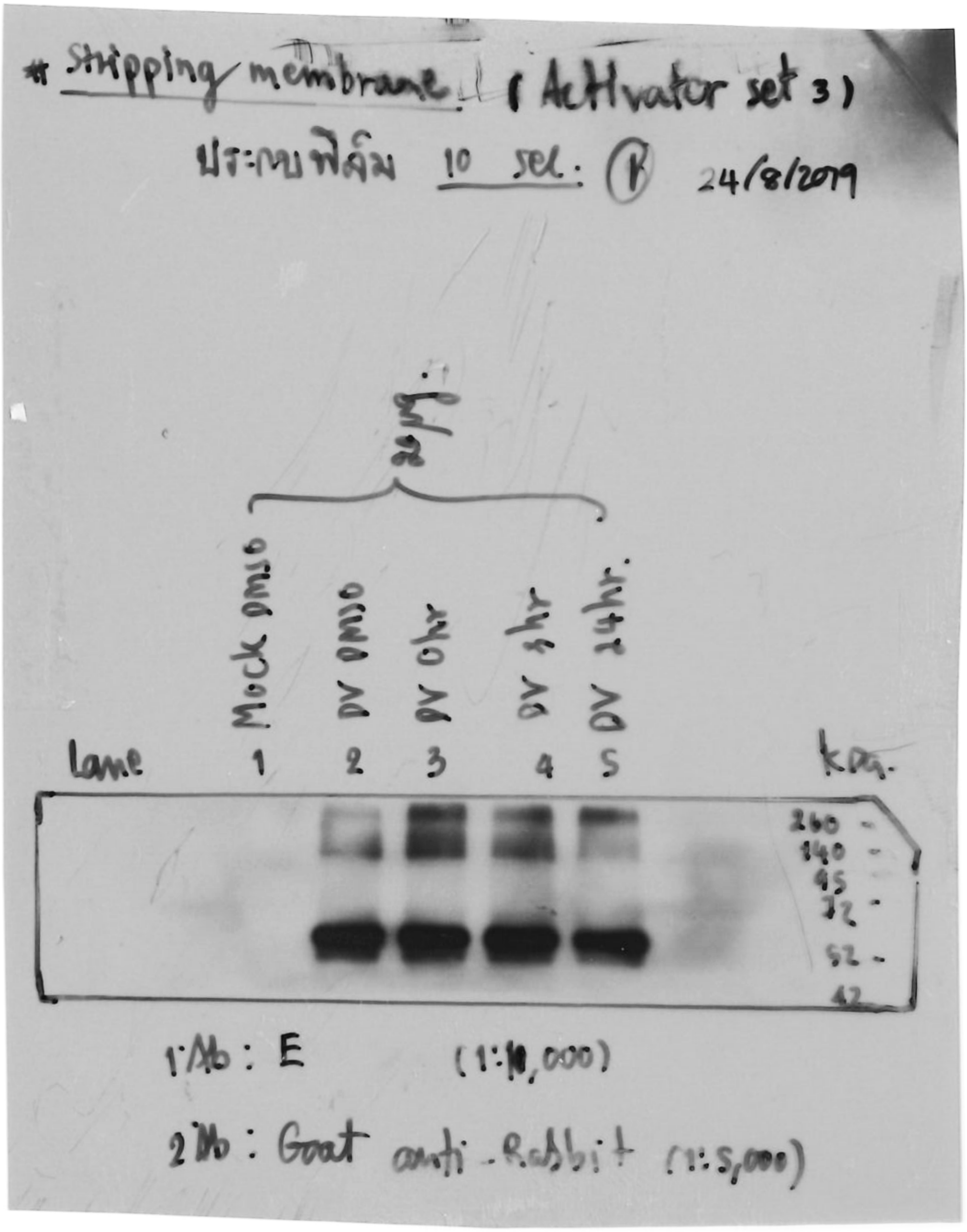

Full uncropped western blot image for figure 6
GAPDH protein: set 3

Immunoblot: GAPDH

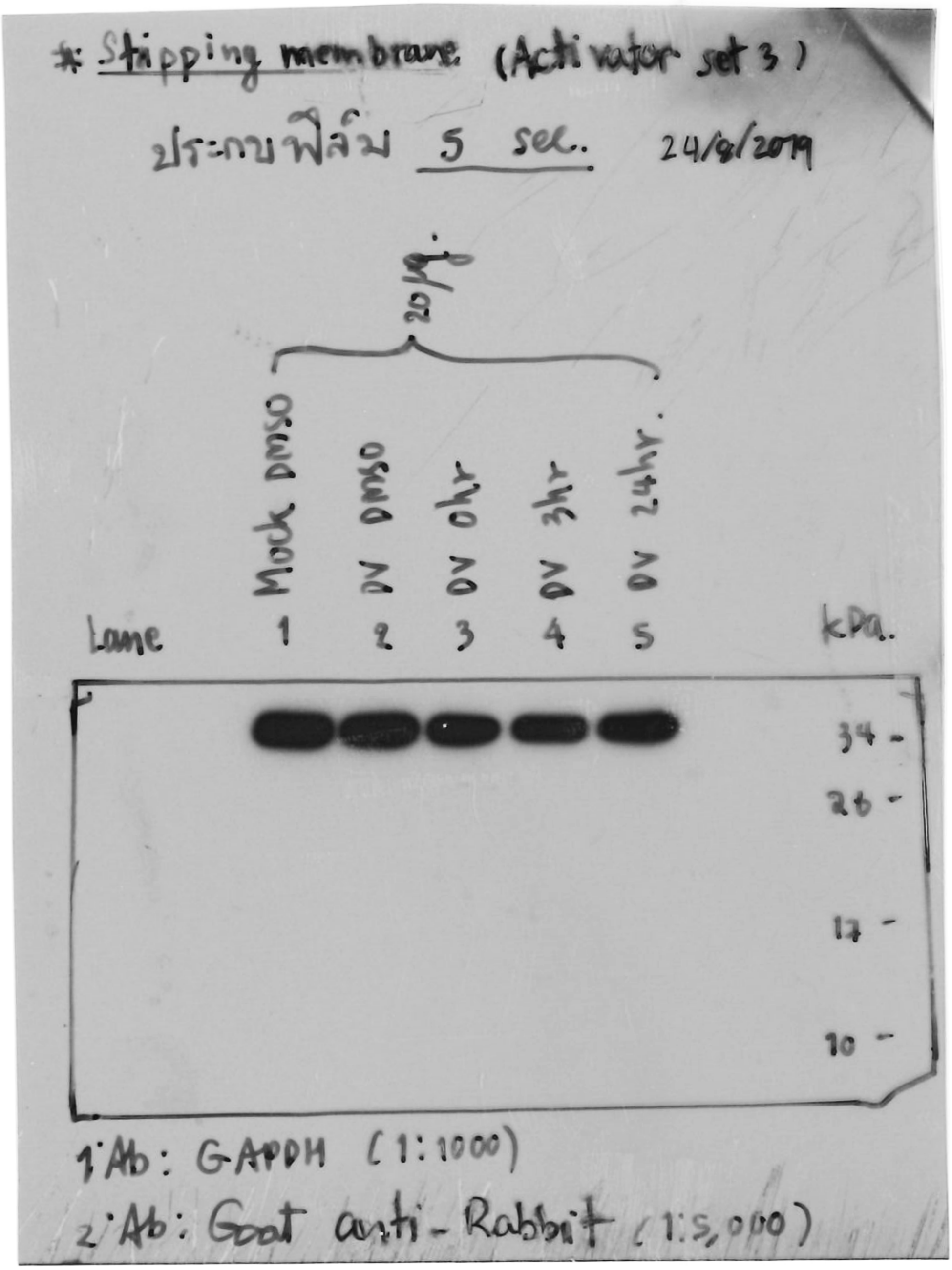

Full uncropped western blot image for figure 6
NS5 protein: set 1 (used in manuscript figure 6B)

Immunoblot: NS5 protein

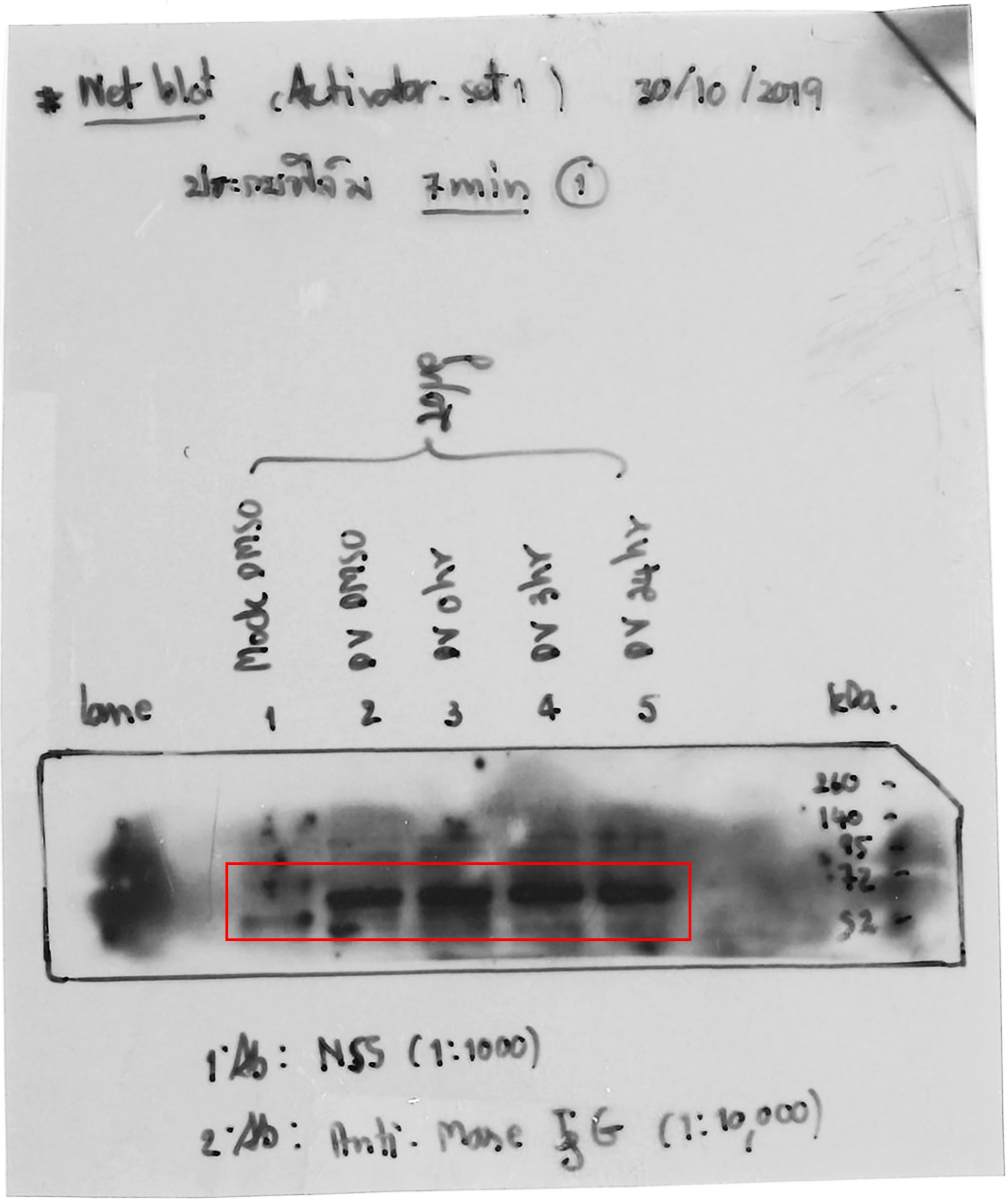

Red box represents the cropped image used in manuscript figure 6B.

Full uncropped western blot image for figure 6
GAPDH protein: set 1 (used in manuscript figure 6B)

Immunoblot: GAPDH

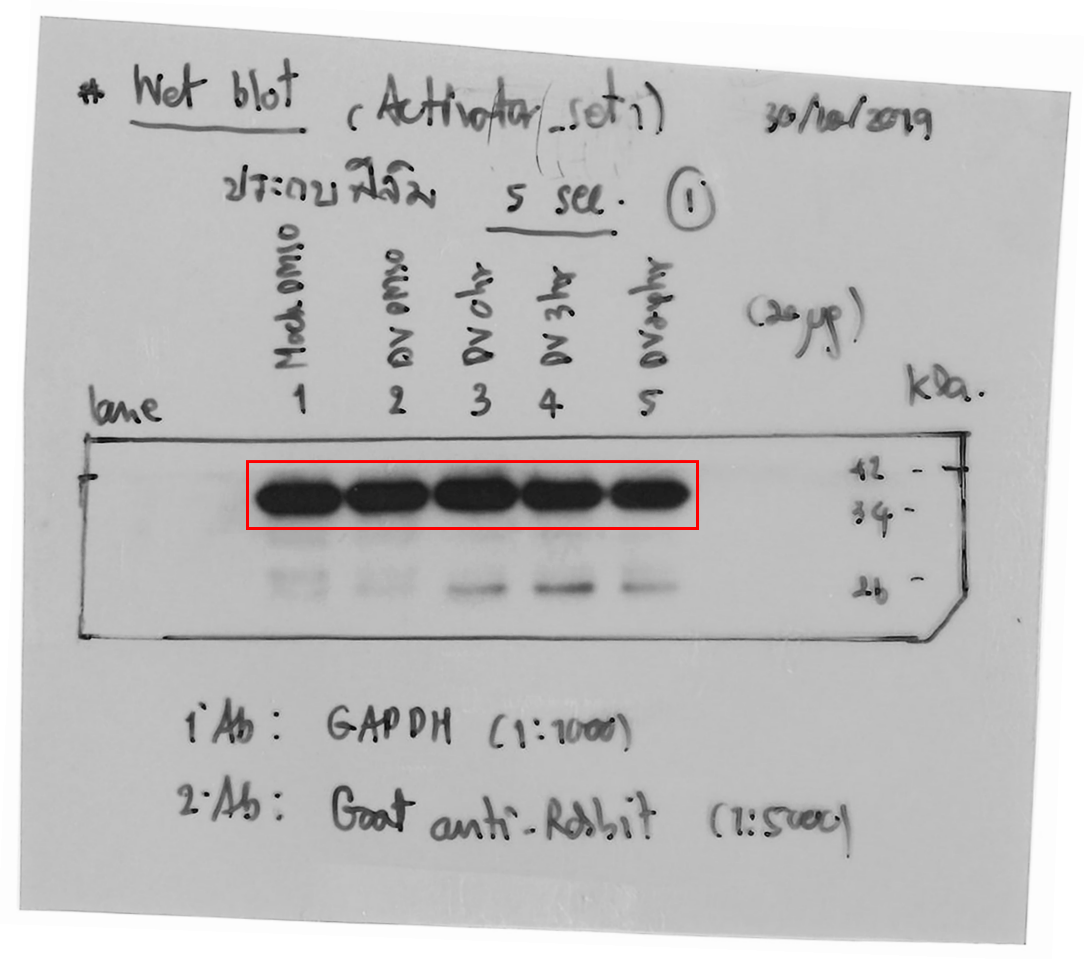

Red box represents the cropped image used in manuscript figure 6B.

Full uncropped western blot image for figure 6
NS5 protein: set 2

Immunoblot: NS5 protein

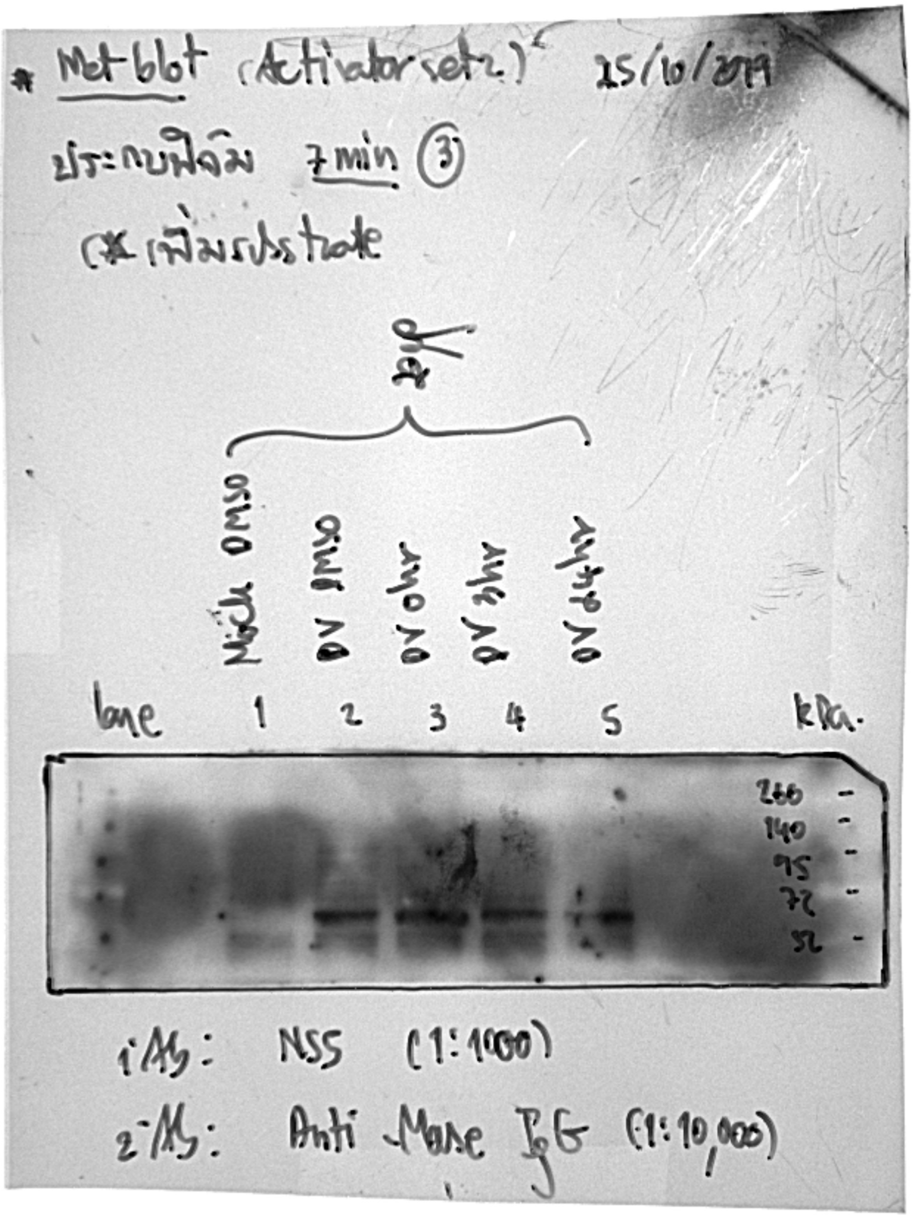

Full uncropped western blot image for figure 6
GAPDH protein: set 2

Immunoblot: GAPDH

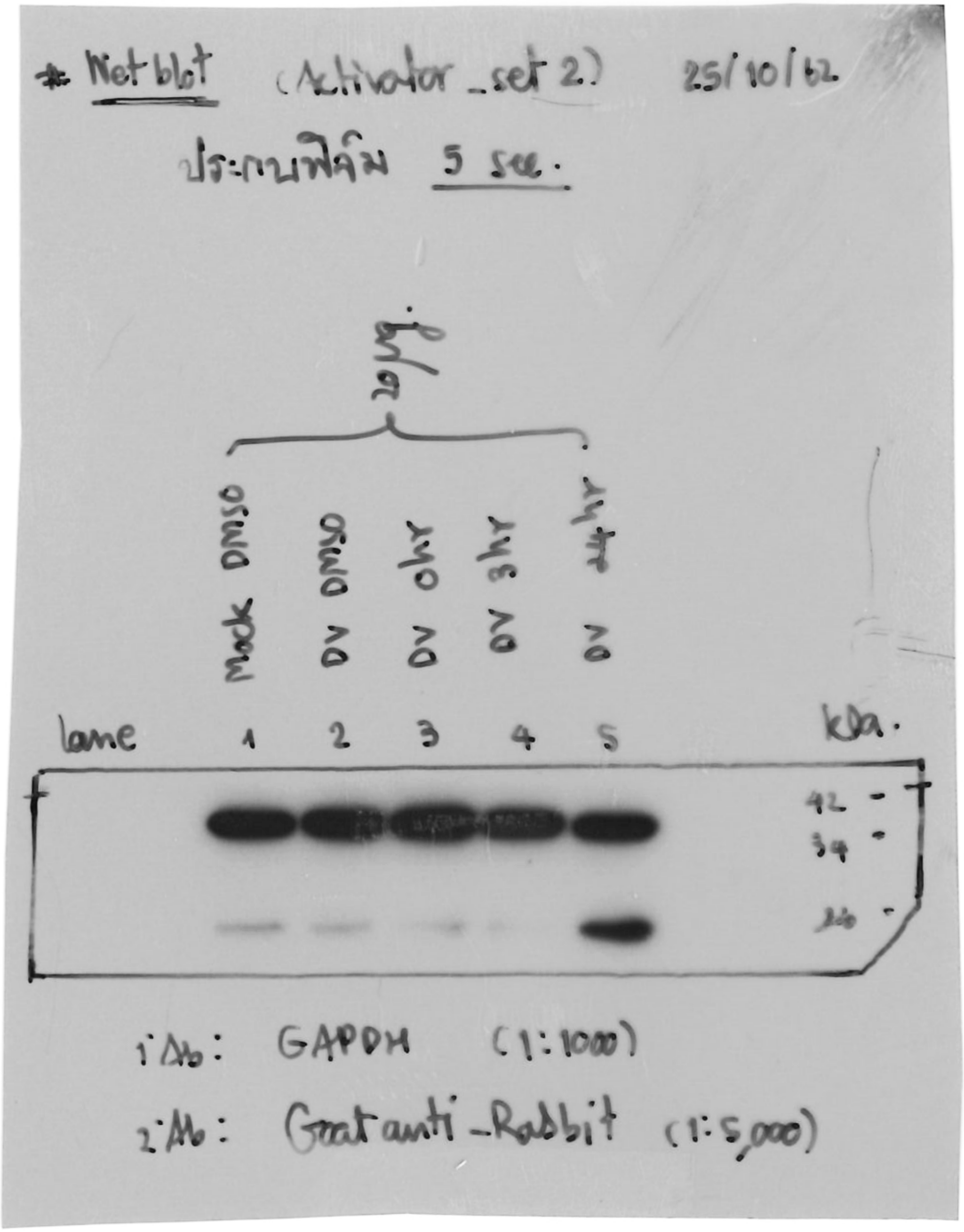

Full uncropped western blot image for figure 6
NS5 protein: set 3

Immunoblot: NS5 protein

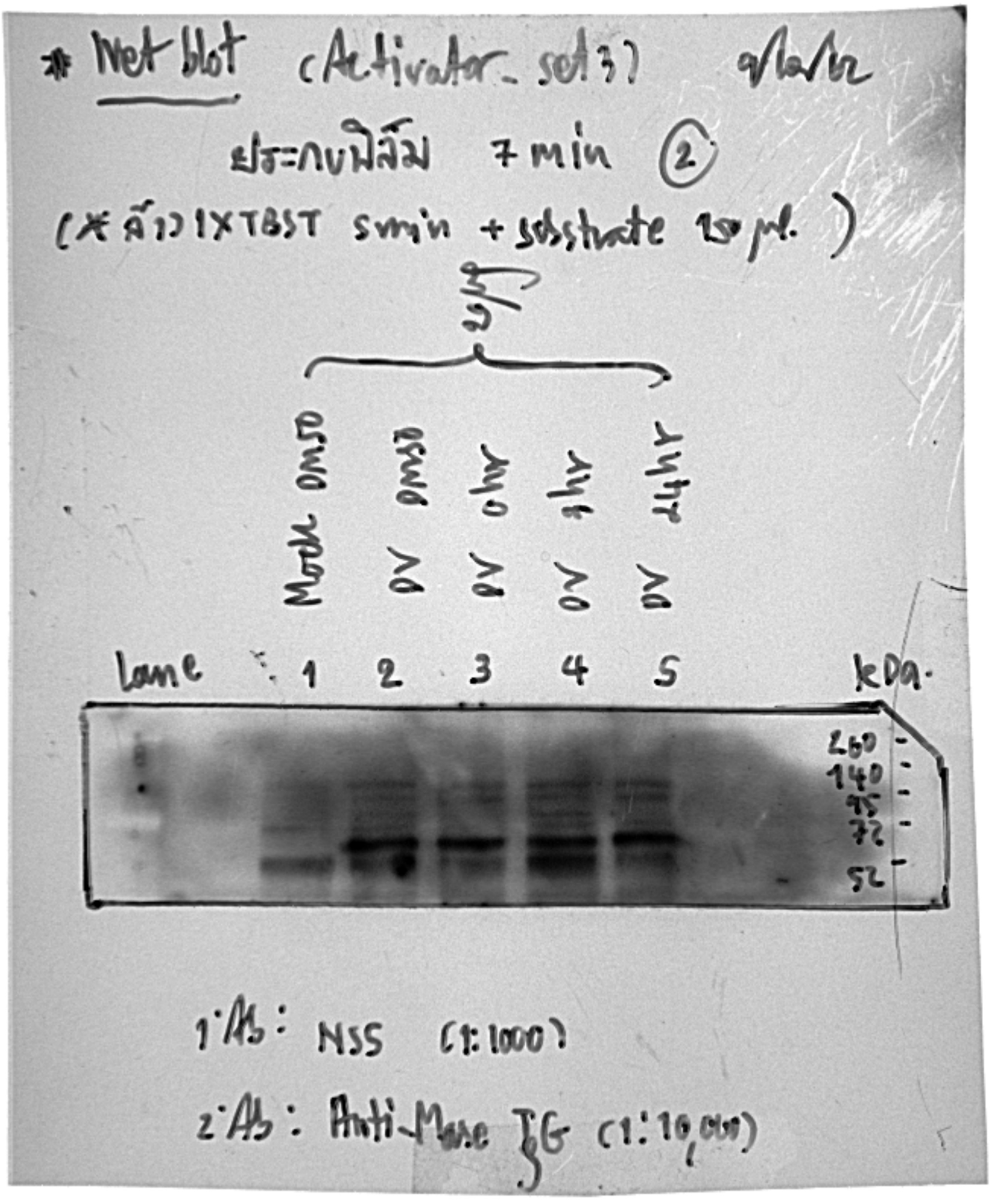

Full uncropped western blot image for figure 6
GAPDH protein: set 3

Immunoblot: GAPDH

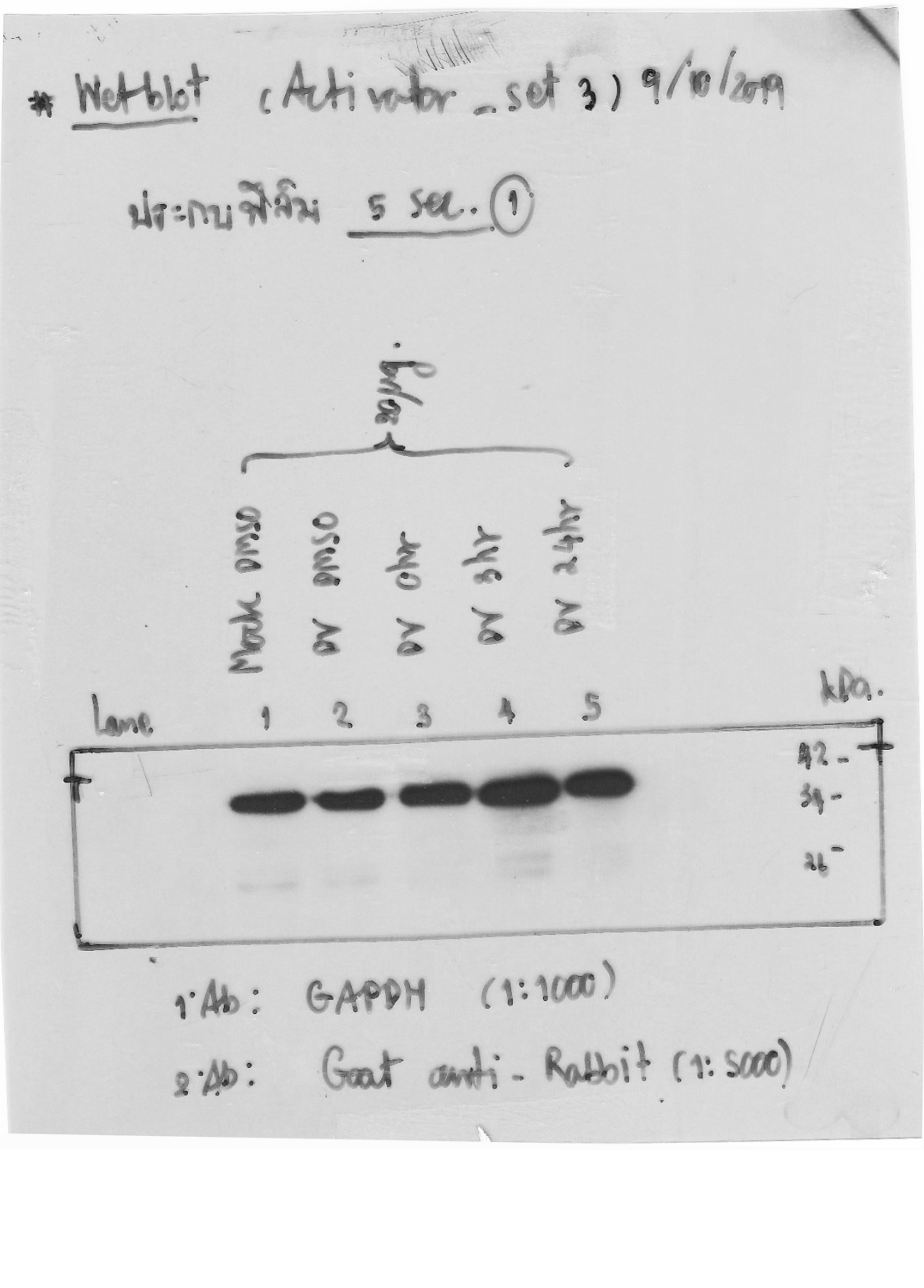

Supplement: Supplementary file 1 — Supplementary file1. [file 41598_2020_71407_MOESM1_ESM.pdf]
